# Genomics reveals the history of a complex plant invasion and improves the management of a biological invasion from the South African–Australian biotic exchange

**DOI:** 10.1002/ece3.9179

**Published:** 2022-08-23

**Authors:** Dennis Byrne, Armin Scheben, John K. Scott, Bruce L. Webber, Kathryn L. Batchelor, Anita A. Severn‐Ellis, Ben Gooden, Karen L. Bell

**Affiliations:** ^1^ CSIRO Health & Biosecurity Floreat Western Australia Australia; ^2^ School of Biological Sciences University of Western Australia Crawley Western Australia Australia; ^3^ Western Australian Biodiversity Science Institute Perth Western Australia Australia; ^4^ CSIRO Health and Biosecurity Canberra Australian Capital Territory Australia; ^5^ Centre for Sustainable Ecosystem Solutions School of Earth, Atmospheric and Life Sciences, University of Wollongong Wollongong New South Wales Australia; ^6^ Simons Center for Quantitative Biology, Cold Spring Harbor Laboratory Cold Spring, Harbor New York USA

**Keywords:** alien, biosecurity, *Chrysanthemoides*, introduction history, invasion genetics, single nucleotide polymorphism

## Abstract

Many plants exchanged in the global redistribution of species in the last 200 years, particularly between South Africa and Australia, have become threatening invasive species in their introduced range. Refining our understanding of the genetic diversity and population structure of native and alien populations, introduction pathways, propagule pressure, naturalization, and initial spread, can transform the effectiveness of management and prevention of further introductions. We used 20,221 single nucleotide polymorphisms to reconstruct the invasion of a coastal shrub, *Chrysanthemoides monilifera* ssp. *rotundata* (bitou bush) from South Africa, into eastern Australia (EAU), and Western Australia (WAU). We determined genetic diversity and population structure across the native and introduced ranges and compared hypothesized invasion scenarios using Bayesian modeling. We detected considerable genetic structure in the native range, as well as differentiation between populations in the native and introduced range. Phylogenetic analysis showed the introduced samples to be most closely related to the southern‐most native populations, although Bayesian analysis inferred introduction from a ghost population. We detected strong genetic bottlenecks during the founding of both the EAU and WAU populations. It is likely that the WAU population was introduced from EAU, possibly involving an unsampled ghost population. The number of private alleles and polymorphic SNPs successively decreased from South Africa to EAU to WAU, although heterozygosity remained high. That bitou bush remains an invasion threat in EAU, despite reduced genetic diversity, provides a cautionary biosecurity message regarding the risk of introduction of potentially invasive species via shipping routes.

## Graphical abstract

Bitou bush is a coastal shrub, native to Southern Africa, and invasive in Australia. We used single nucleotide polymorphisms to reconstruct the invasion history of bitou bush into eastern and Western Australia. We found strong genetic structure in the native range, and detected bottlenecks through the introduction process. Our results highlight the risk of introduction of potentially invasive species via shipping routes, even from a small number of propagules.

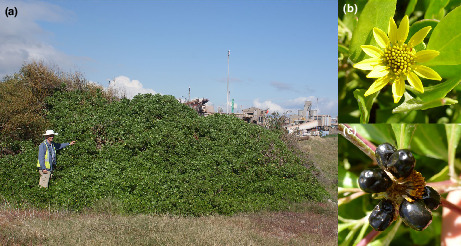

## 1 INTRODUCTION

The extensive intercontinental movement of people and products associated with globalization has facilitated biological invasions into managed and unmanaged environments and significantly threatens native biodiversity, economic and social values (Hulme, 2009; Mollot et al., 2017; Pyšek, Hulme, et al., 2020; Westphal et al., 2008). The risk of invasion and spread varies with introduction pathway (Riera et al., 2021; Wilson et al., 2009), propagule pressure (Blackburn et al., 2015; Simberloff et al., 2013), and genetic diversity of the founding population (Estoup et al., 2016). Knowing the origin of introduced material also has implications for the success of classical biological control (Gaskin et al., 2011; Roderick & Navajas, 2003). Consequently, there is a strong incentive to better understand the ecological context of the invasion to improve the efficiency and effectiveness of evidence‐based management strategies (DiTomaso, 2000; Pheloung et al., 1999).

A critical component of improving management is understanding early invasion stages, including introduction pathways, naturalization, and initial spread (Estoup & Guillemaud, 2010; Faulkner et al., 2020; Richardson et al., 2003). Detailed longitudinal studies of introduced pests and weeds are uncommon, particularly those that capture the introduction pathway and that include insight soon after their arrival into a new region. However, a history of the introduction can be inferred using approaches combining molecular genetics with traditional biogeographical theory. In particular, such studies can provide valuable insight to determine the origin of and reconstruct historical demographics for introduced populations (Cristescu, 2015; Kamenova et al., 2017). The understanding of historical invasion context achieved through molecular methods can help to predict, assess, and guide the effectiveness of management strategies (Chown et al., 2015; Sherpa & Despres, 2021).

Reconstruction of the invasion history and identification of source populations can highlight introduction pathways in order to prevent future introductions. This insight is particularly informative for target taxa with strong genetic structure or cryptic speciation in the native range, because divergent genotypes or cryptic species may differ in their susceptibility to biological control agents (Goolsby et al., 2005; Manrique et al., 2008; Paterson et al., 2019; Smith et al., 2018) or their suitability to the range of climatic niches available in the introduced range (Zenni et al., 2014). Molecular methods can also inform management by helping understand population connectivity via current and historical dispersal patterns (Nobarinezhad et al., 2020), delimiting populations for management or local extirpation (Hampton et al., 2004), and understanding the rate of invasion (i.e., expansion) for poorly documented introductions (Kamenova et al., 2017; Novak & Mack, 2001).

Insights into the genetic diversity of invasive species that can be gained through molecular methods can also provide insight into the propagule pressure of the invasion (Fitzpatrick et al., 2011). Propagule pressure is an important factor in the successful establishment of invasive populations, through lessening effects of demographic stochasticity, Allee effects, environmental heterogeneity, genetic drift, and inbreeding depression (Blackburn et al., 2015; Simberloff et al., 2013). Mechanisms such as population admixture, introgressive hybridization, ongoing gene flow, and rapid population growth following bottlenecks of short duration can enable introduced populations to overcome large genetic loads (Bock et al., 2015; Colautti et al., 2017; Dlugosch & Parker, 2008; Kolbe et al., 2004; Prentis et al., 2008; Rius & Darling, 2014; Smith et al., 2020). In other cases, polyploidy provides a range of mechanisms for facilitating plant invasions, such as preadaptation to conditions in the introduced range, restoring sexual reproduction following hybridization, and conversely, overcoming Allee effects by enabling asexual reproduction (te Beest et al., 2012). Molecular analysis of introduced populations can determine propagule pressure and determine whether advantages gained by introduced species through evolutionary and demographic processes may hinder control attempts.

In the past decade, new genomics methods allowing researchers to examine thousands of loci at the population level have increased our power for understanding complex introduction pathways and evolution postintroduction. For example, studies of the invasive weed *Centaurea solstitialis* (Asteraceae) revealed a stepwise invasion history, starting with an early introduction into Western Europe from two source populations, which then served as a genetic bridgehead for invasions into Chile and then California, followed by evolution of increased plant size in the Californian population (Barker et al., 2017; Eriksen et al., 2014). Other studies have found that introduced populations have overcome bottlenecks and maintained genetic diversity through complex introduction histories involving multiple source populations (e.g., *Frangula alnus*; De Kort et al., 2016). Furthermore, by analyzing a large number of loci, it may be possible to determine population genetic diversity and differentiation using much smaller sample sizes than needed for traditional methods such as microsatellites (Qu et al., 2020). This insight is of particular relevance for studies of invasion history where many populations need to be analyzed.

There are few study systems where the value of this improved insight is more relevant to transforming our understanding of biotic invasions to enhance management outcomes than for the biotic exchange between Australia and South Africa (Pyšek, Pergl, et al., 2020). The exchange of invasive plants between Australia and South Africa contributes disproportionately to the most threatening of invasive species globally (Pyšek, Pergl, et al., 2020). These include both deliberate introductions for agricultural, ornamental, and environmental (e.g. dune stabilization) reasons (e.g., *Asparagus asparagoides*; Morin et al., 2009) and numerous accidental introductions as contaminants of livestock, machinery, and ship ballast (e.g., *Senecio madagascariensis*; Wijayabandara et al., 2022). Many South Africa taxa, in particular the Iridaceae, appear to be on the verge of becoming invasive in Australia (Pyšek, Pergl, et al., 2020) and thus increasing the scale of the problem.

With multiple pathways of and rationales for introduction, the complexity of these invasion histories has made subsequent weed management particularly challenging. Considerable effort has been spent on managing the worst of the exchanged weeds in both countries, including eradication attempts (e.g., *Chrysanthemoides monilifera* ssp. *rotundata*; Scott, Batchelor, & Webber, 2019) and classical biological control programs (e.g., *Asparagus asparagoides*; Morin et al., 2022). However, both countries have ecosystems spanning a wide range of climates and many of the most problematic weeds occupy a broad realized niche (e.g., *Lycium ferrocissium*; McCulloch et al., 2020). This situation has made managing these species particularly difficult. For example, biological control programs have failed due to an apparent mismatch between agent and target biotypes, the most notable case is of the misidentification of *Salvinia molesta* (Julien, 2012), while multiple introductions of *Acacia saligna* in a range of global locations did not show a consistent pattern, indicating that invasion history can be very local (Thompson et al., 2015). Applying new genomic techniques to the complex invasion management scenarios that characterize the biotic exchange between South Africa and Australia is likely to help solve management quandaries for multiple species, while also providing broader insight for mitigating future invasion risks.

To provide broader insight on the exchange of invasive weeds between South Africa and Australia and to focus in particular on a problematic study system, we investigated the invasion history of *Chrysanthemoides monilifera* ssp. *rotundata* (DC.) T.Norl. (bitou bush). Bitou bush is a shrub native to coastal subtropical regions in the Eastern Cape and KwaZulu Natal provinces of South Africa. The plant was first recorded in Australia in 1908 near the port city of Newcastle, New South Wales (NSW), hypothesized to have been inadvertently introduced in dry ship's ballast (Weiss et al., 2008). The subsequent naturalization and invasion were accelerated between 1946 and 1968 when bitou bush was deliberately planted as a dune stabilizing species in coastal areas of NSW (Weiss et al., 2008). No evidence exists regarding the source of the seed used for these plantings (i.e., locally collected or single/multiple introductions from the native range). Bitou bush can now be found invading 44,000 hectares of coastal landscapes in eastern Australia (Hamilton et al., 2012). Due to its impact on the environment and high invasiveness, bitou bush is listed as a Weed of National Significance in Australia and has been subject to containment and localized extirpation efforts since 1982 (Cherry et al., 2008).

Until recently, it was thought that the Australian invasion was restricted to the east coast, largely within the state of New South Wales. However, a recent introduction leading to a naturalized population of about 1700 plants was discovered in 2012 in the coastal suburb of Kwinana, Western Australia (Scott et al., 2016). This introduction was dated using aerial photography to 1995 and has been hypothesized to have originated from eastern Australia via shipping activity, given that a nearby port has links to the east coast of Australia (Scott & Batchelor, 2014; Scott, Batchelor, & Webber, 2019). However, as for the hypotheses for the east coast introduction, there is no published evidence for or against a shipping‐mediated introduction pathway.

In an effort to better understand the introduction and invasion history as well as the genetic diversity of bitou bush in its non‐native range, we applied de novo double digest restriction associated DNA sequencing (ddRADseq; Peterson et al., 2012) to generate SNPs from populations sampled in the native range of South Africa and the introduced range in Australia, and carried out flow cytometry analysis of the more recently introduced Western Australian population to assess the genome size and infer ploidy. Specifically, we aimed to (1) assess bitou bush population diversity, genetic structure, admixture, and ploidy; (2) compare hypothesized introduction scenarios for bitou bush using Bayesian modeling; and (3) using this insight, assess the likely effectiveness of current management strategies for bitou bush in Australia. These findings are placed into the broader context of how new genomic methods can be used to improve invasion management outcomes in general, and for the South African–Australian exchange of weeds in particular.

## 2 MATERIALS AND METHODS

### 2.1 Study species

**FIGURE 1:**
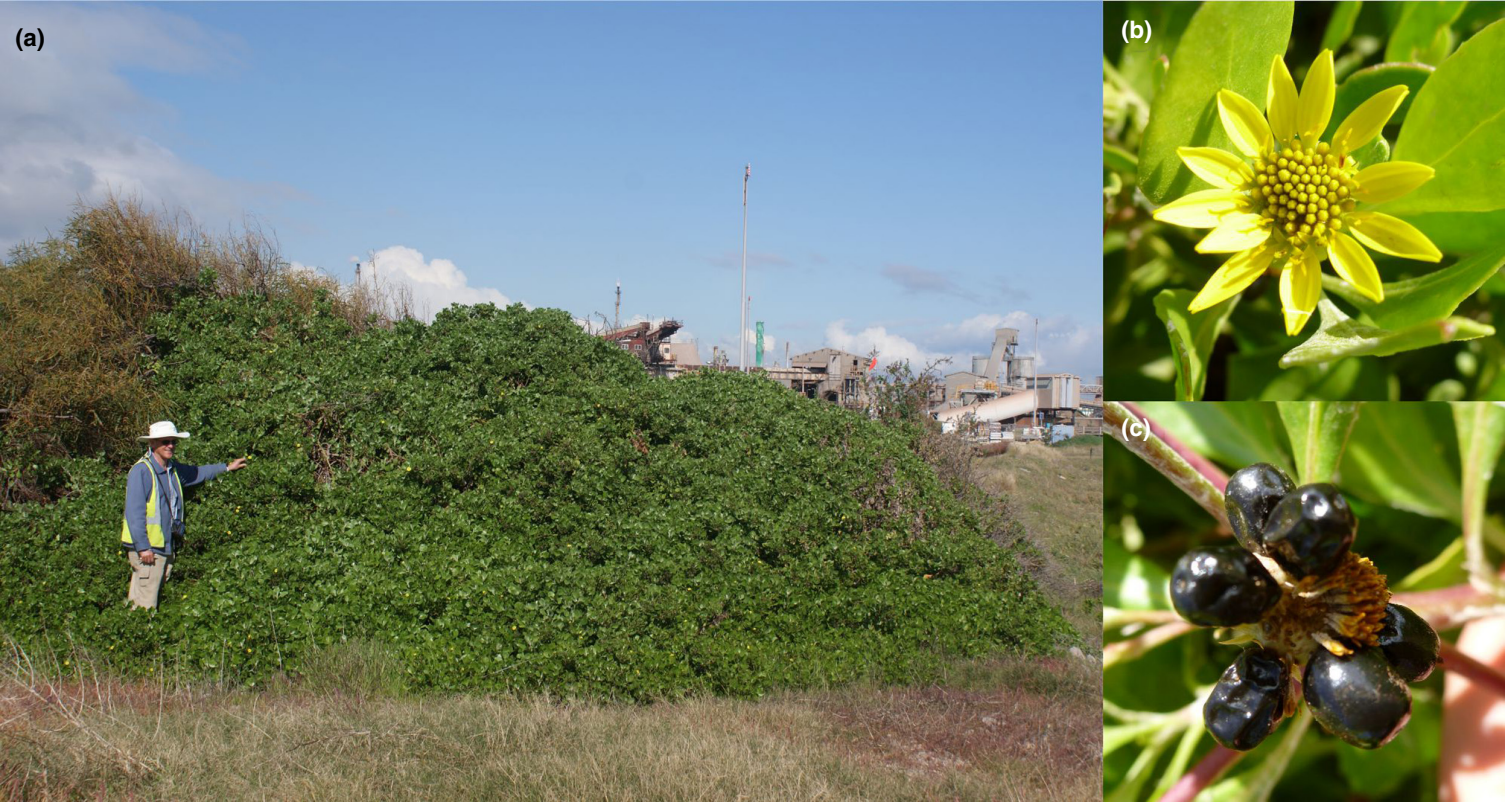
*Chrysanthemoides monilifera* ssp. *rotundata* (bitou bush) at Kwinana Industrial Estate in Western Australia. (a) shows a large adult bush, possibly one of the initial colonizers. In the background, there is a mineral processing plant. On the left, above the person, is an acacia shrub evidently being displaced by the bitou bush. (b) composite flower, and (c) ripe fruits that each contain one seed. Photos: a: John K. Scott, CSIRO; b and c: Kathryn L. Batchelor, CSIRO.

The genus *Chrysanthemoides* belongs in the tribe Calenduleae of the Asteraceae family (Bayer & Starr, 1998; Norlindh, 1977). The genus comprises two species native to southern Africa with one, *C. monilifera*, divided into a variable number of infraspecific taxa (Barker et al., 2015) among which two (boneseed: *C. monilifera* ssp. *monilifera* and bitou bush: *C. monilifera* ssp. *rotundata*) have been introduced into Australia (Weiss et al., 2008). Bitou bush is found in coastal sand dunes and adjacent areas in a native range from subtropical regions near Cape St Francis to tropical regions near the South Africa‐Mozambique border (Hamilton et al., 2012; Scott, 1996; Weiss et al., 2008). The closely related boneseed occupies coastal areas and further inland into the adjacent mountains in drier more Mediterranean climates of the south‐western and south‐eastern parts of South Africa (Weiss et al., 2008). Mature bitou bush shrubs vary in size from 0.5 to 2 m^2^ canopy area in its native range (Scott, 1996), and up to 2–3 m high and wide in Australia (Scott, Batchelor, Jucker, & Webber, 2019; Figure 1). Reproduction is primarily by seed but can also include stem layering (Weiss, 1984). Unusually for members of Asteraceae, a bitou bush seed develops inside a fleshy fruit (i.e. drupe), which is consumed and dispersed by frugivorous species, chiefly birds (Gosper, 2004). This trait is expected to lead to a high capacity for dispersal and spread. Pollination has not been studied in detail, but appears to be by generalist insects (Weiss et al., 2008). Evidence from glasshouse experiments (Gross et al., 2017) and field observation (Scott, Batchelor, Jucker, & Webber, 2019) points to bitou bush being an obligate outcrossing taxon. Vegetative reproduction is rare (Scott, Batchelor, & Webber, 2019), but when it does occur, it is mainly from plants in mobile dunes or as a consequence of stem sections being covered by soil. The impact of bitou bush on ecosystems is via competitive displacement of native vegetation, production of allelopathic compounds, and alteration of soil biogeochemical cycling (Ens et al., 2009; Lindsay & French, 2004; Mason & French, 2008).

Management goals in the introduced Australian range vary according to local context and include eradication, containment, and protection of sensitive environments. Management in eastern Australia is focussed on decreasing the density of bitou bush, with reasonable success, although it continues to spread to new locations (Hamilton et al., 2012). Regional extirpation attempts (i.e., containment) are being made at the northern and southern limits of the distribution in eastern Australia. At the northern limit, eradication is being attempted on K'gari‐Fraser Island (Behrendorff et al., 2019). Populations have been substantially reduced, but there is a risk of reintroduction from uncontrolled populations on the nearby mainland (Behrendorff et al., 2019). Regional extirpation is also being attempted at the southern limit of the range in Victoria, but this may be complicated by hybridization with boneseed (Adair & Butler, 2010). In Western Australia, eradication from the entire state is being attempted and may be more feasible, although the current management program still has years to run (Scott, Batchelor, & Webber, 2019).

### 2.2 Sampling

**FIGURE 2:**
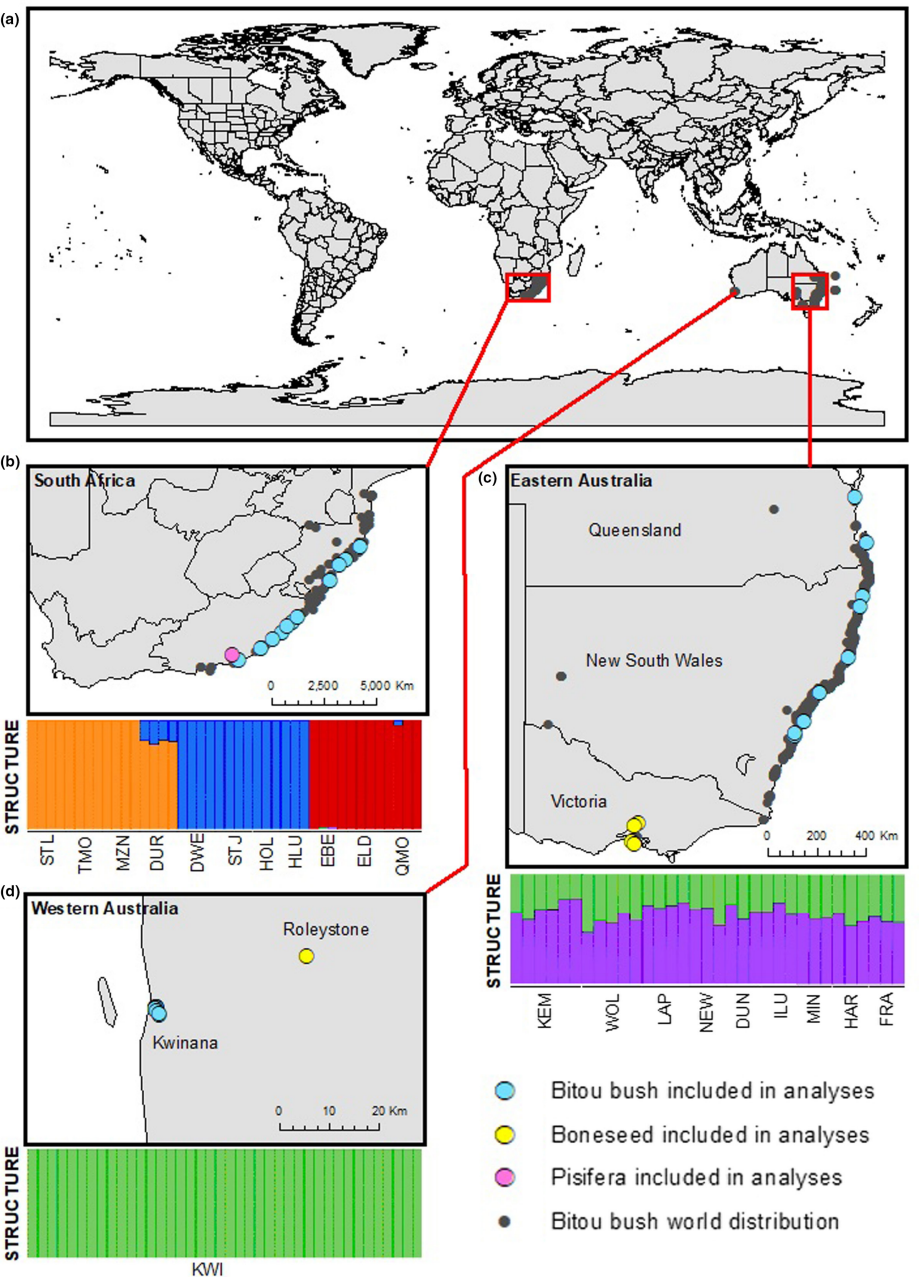
(a) World map with, inserts for (b) southern Africa, (c) eastern Australia and (d) Western Australia (Kwinana Industrial Estate) showing distribution of bitou bush (based on records from GBIF for southern Africa and eastern Australia; and Scott, Batchelor, & Webber (2019) for Western Australia. Larger dots show sample locations of *Chrysanthemoides monilifera* used in this study (listed in Table S1). FASTSTRUCTURE analysis is for *K* = 5 based on ssp. *rotundata* (bitou bush) only. South African localities listed from north to south: DUR, Durban; DWE, Dwesa; EBE, East Beach; ELD, East London; HLU, Hluleka; HOL, Hole in the Wall; MZN, Mtunzini; STJ, Port St. John; STL, St Lucia; TMO, Tugela Mouth; QMO, Qolora Mouth. Eastern Australian localities listed from south to north: KEM, Port Kembla; DUN, Dunbogan; FRA, Fraser Island; HAR, Harvey Bay; ILU, Iluka; LAP, LaPerouse; MIN, Minnie Waters; NEW, Newcastle; WOL, Wollongong.

We sampled 119 bitou bush plants from populations across the native range in South Africa (43 individuals, 11 populations; Figure 2, Table 1, Table S1), the full extent of its introduced range in eastern Australia (EAU: 36 individuals, 9 populations), and the single population in Western Australia (WAU: 40 individuals). Sampled populations were separated by at least 10 km. Within each population, we sampled plants at least 5 m apart to ensure they were different individuals. Populations that were likely to include hybrids between bitou bush and boneseed were avoided. The majority of sampling was carried out between 2017 and 2018 for all populations, with additional sampling in 2012 from the Kwinana population in WAU (Scott & Batchelor, 2014). Australian boneseed (20 individuals from 5 populations in Victoria, EAU, and 6 individuals from one population in WAU) and *C. monilifera* ssp. *pisifera* (pisifera) from South Africa (4 individuals from 1 population from Grahamstown, Eastern Cape Province) were sampled during 2017–2018 and used as outgroups in genetic analyses. All leaf material was desiccated immediately after sampling with silica gel beads (Chase & Hills, 1991), samples were stored and transported at ambient temperature, and approximately 100 mg desiccated leaf from each sample was used in DNA extractions. Whole plants were taken from the Kwinana population and grown in a quarantine glasshouse in Floreat, Western Australia, for flow cytometry.

**TABLE 1:** Collection regions and localities of samples of *Chrysanthemoides monilifera* (bitou bush and relatives) used in this study, and acronyms used for source regions in subsequent analyses

Region	Locality	Latitude	Longitude	Subspecies	Number of samples
Western Australia (WAU)	Kwinana	32.211° S	115.767° E	Bitou bush	35
	Roleystone	32.126° S	116.061° E	Boneseed	6
Eastern Australia (EAU)	Dunbogan, New South Wales (NSW)	31.648° S	152.834° E	Bitou bush	3
	Iluka, NSW	29.420° S	153.362° E	Bitou bush	3
	La Perouse, NSW	33.988° S	151.234° E	Bitou bush	4
	Minnie Water, NSW	29.782° S	153.296° E	Bitou bush	3
	Newcastle, NSW	32.920° S	151.780° E	Bitou bush	3
	Port Kembla, NSW	34.470° S	150.920° E	Bitou bush	6
	Wollongong, NSW	34.470° S	150.900° E	Bitou bush	7
	Fraser Island, Queensland (QLD)	25.751° S	153.087° E	Bitou bush	3
	Harvey Bay, QLD	27.436° S	153.539° E	Bitou bush	3
	Arthur's Seat, Victoria (VIC)	37.695° S	145.172° E	Boneseed	6
	Eltham Aqueduct, VIC	37.694° S	145.172° E	Boneseed	2
	Fairfield Park, VIC	37.790° S	145.016° E	Boneseed	3
	Flinders coastline, VIC	38.480° S	145.009° E	Boneseed	3
South Africa – native range south (NRS)	East Beach	33.602° S	26.899° E	Bitou bush	3
	East London	33.033° S	27.911° E	Bitou bush	4
	Qolora Mouth	32.647° S	28.428° E	Bitou bush	4
	Fairewood	33.327° S	26.553° E	Pisifera	4
South Africa– native range central (NRC)	Dwesa	32.305° S	28.832° E	Bitou bush	4
	Hluleka	31.828° S	29.303° E	Bitou bush	3
	Hole in the Wall	32.039° S	29.106° E	Bitou bush	4
	Port St Johns	31.624° S	29.548° E	Bitou bush	3
South Africa– native range north (NRN)	Durban	29.902° S	31.040° E	Bitou bush	4
	Mtunzini	28.957° S	31.763° E	Bitou bush	4
	St Lucia	28.363° S	32.433° E	Bitou bush	4
	Tugela Mouth	29.221° S	31.501° E	Bitou bush	4

### 2.3 DNA isolations and ddRADseq library preparation

Genomic DNA was extracted from desiccated leaf material using a DNeasy Plant Mini Kit (QIAGEN, Hilden, Germany) with minor modifications for semi‐succulent leaves (Pettigrew et al., 2012). To maximize final DNA yields, three replicates of each sample were processed in parallel up to step 13 of the manufacturer's protocol, and then the cleared lysate of the three replicates was passed through a single spin column and the captured DNA eluted in 100 μl of EB buffer. Where DNA yields remained low (<12 ng/μl), samples were concentrated into 12 μl using a DNA Clean and Concentrator Kit (Zymo Research, Irvine, USA). Final genomic DNA concentrations were measured using a High Sensitivity (HS) Qubit 3.0 Fluorometer assay (Invitrogen, Carlsbad, USA) and DNA quality assessed using a LabChip GX Touch 24 (PerkinElmer, Hopkinton, Massachusetts).

A modified version of the ddRADseq protocol (Peterson et al., 2012) was used to construct libraries from the isolated genomic DNA (Severn‐Ellis et al., 2020). Each sample was digested for 4 h at 37°C. Digestion reactions contained 200 ng of genomic DNA, 2 μl of NEB CutSmart Buffer (10×), 5 units (0.5 μl) each of the restriction enzymes HpyCH4IV and Hinfl (New England Biolabs, Ipswich, USA) and nuclease free water to a volume of 20 μl. Barcoded and common adapters designed to complement the restriction enzyme pair's overhangs were prepared as described by Peterson et al. (2012). The digested DNA of each sample was ligated to the unique barcoded sequence in a master mix containing the barcoded adapter (0.23 μM) and common adapter (0.5 μM) using T4 DNA ligase (Invitrogen, Carlsbad, USA). The entire ligated DNA product was purified and size selected in two steps to enable enrichment of fragments between 250 and 800 bp. The first size selection step was carried out by increasing the volume of the ligated sample to 100 μl with nuclease free water. Fragments >800 bp were then removed by adding 50 μl of a 1:4 mixture of AMPure XP Beads (Beckman Coulter, Brea, USA) to PEG buffer (20% PEG w/v, 2.5 M NaCl). The resulting supernatant was collected and added to 20 μl of a 1:1 AMPure XP Beads to PEG buffer mixture in the second size selection step to retain fragments >250 bp. The beads were washed using 80% ethanol and the size‐selected DNA eluted in 30 μl nuclease free water. A 10 μl aliquot of the size‐selected DNA was enriched using Phusion Hot‐Start High‐Fidelity Polymerase Master Mix (Thermo Fisher Scientific, Waltham, USA), Indexed PCR2 primer (0.5 μM), and PCR1 (0.5 μM; primer described by Peterson et al., 2012). Amplified libraries were cleaned using 1.50× reaction volume of AMPure XP Beads and the DNA concentrations determined by HS Qubit assay. Equimolar amounts of the prepared libraries were pooled and loaded on a 1.5% agarose gel to enrich and select fragments between 300 and 700 bp. The DNA was recovered using the QIAquick Gel Extraction Kit (QIAGEN, Hilden, Germany). The final library quality, size distribution, and concentration were assessed on the LabChip GX Touch and Qubit HS assay, followed by dilution to 20 nM/μl in 10 nM Tris Buffer (pH 8.5, 0.1% Tween 20, 10 nM). The final ddRADseq libraries were sent to Australian Genome Research Facility (AGRF; Melbourne, Australia) for sequencing of 100 bp reads across three Illumina HiSeq 2500 lanes. Genomic data used for this project are available at NCBI under bioproject PRJNA525912 (https://www.ncbi.nlm.nih.gov/sra/PRJNA525912).

### 2.4 Assembly of RAD loci and SNP calling

All scripts used for data analysis are available at https://github.com/ascheben/bitou_analysis. Single end reads were demultiplexed using stacks 2.1 *process_radtags*, (Rochette et al., 2019) with barcode rescue (−r), quality filtering (−q, −c) and read length trimming to 95 bp (−t 95). The number of reads generated per individual was even (median 6.73 M). A single individual with less than 1 M reads was removed. Reads containing adapters were discarded using trimmomatic 0.36 (Bolger et al., 2014), and all reads without the enzyme recognition site (CGT) were also discarded. Quality checking of the filtered reads per individual (median 5.72 M) was conducted with fastqc 0.1.11 (Andrews, 2010) and multiqc 1.0 (Ewels et al., 2016). The mean per base quality (Phred +33 score) across all individuals was 36.4. Read processing results are summarized in Table S2.

For further assembly and SNP calling, individuals were split into two levels of inclusion: “bitou‐pisifera” and “bitou‐pisifera‐boneseed.” The bitou‐pisifera group excluded boneseed individuals, which are genetically more distant from the other subspecies. De novo assembly of RAD loci and SNP calling was conducted with stacks 2.1 by manually executing all steps of the *denovo_map* pipeline. After exploring the parameter space (Table S3), different parameters were selected for the bitou‐pisifera and the bitou‐pisifera‐boneseed group. For bitou‐pisifera‐boneseed, a minimum distance of three nucleotides was chosen to identify a stack (−m) and a maximum distance of three nucleotides was permitted between stacks in a locus (−M). A total of three mismatches were allowed between orthologous loci of different individuals during catalogue construction. For bitou‐pisifera, these parameters (−m, −M, −n) were all adjusted to a value of two to address the lower genetic distance within this group. All SNPs were exported in VCF format using stacks *populations*.

SNPs identified in the bitou‐pisifera and bitou‐pisifera‐boneseed groups were filtered using vcftools (Danecek et al., 2011). First, all individuals with >90% missing genotypes were removed. Then, genotype calls with depth <5 were removed. Biallelic SNPs with <20% missing genotypes and minor allele frequency >0.05 were retained. To reduce the influence of linked SNPs from the same RAD locus on population genetic analyses, a single SNP was randomly selected from each locus and the other SNPs discarded. Finally, in a postfiltering check for missingness, individuals with >50% missing genotypes were removed. By maximizing the shared SNP sites between all individuals, we aimed to increase the accuracy of our population genetic analyses (Bohling et al., 2013). Population summary statistics including pairwise *F*_ST_ and AMOVA‐based statistics (*Φ*_ST_ and $F_{\mathrm{ST}}^{\prime }$) were calculated using stacks *populations*. Heterozygosity was calculated using vcftools.

### 2.5 Phylogenetic and population structure analyses

Genetic analysis was carried out to identify relationships and structure within the bitou bush and outgroup populations. All analyses were conducted using both the bitou‐pisifera‐boneseed and the bitou‐pisifera SNP datasets. SNPs were converted to phylip format using a Python script (Ortiz, 2019). As all sites in the multiple sequence alignment were variable SNP sites, a model with ascertainment bias correction (ASC_GTRGAMMA) was used to infer a maximum likelihood phylogeny with raxml 8.2.11 (Stamatakis, 2014) using rapid bootstrapping and 1000 bootstraps. Before the analysis, a custom Python script was used to remove all SNPs without homozygous alternate allele genotypes, as these sites are incompatible with the RAxML parameters. The tree was visualized using ggtree (Yu et al., 2017). A network analysis was also carried out to illustrate the relationships between the admixed populations. To do this, an identity‐by‐state (IBS) distance matrix was calculated from the SNPs using tassel 5.2.50 (Bradbury et al., 2007) with default settings. The IBS matrix was then used to carry out a NeighborNet analysis with splitstree 4.14.8 (Huson, 1998) using default settings. The resulting phylogenetic network was visualized using phangorn (Schliep, 2011). Population genetic structure was analyzed using faststructure 1.0 (Pritchard et al., 2000; Raj et al., 2014). faststructure was run for 1–10 populations (*K*) using the default simple prior and convergence criterion. We selected the optimal value of *K* using faststructure *chooseK* and the method of Puechmaille (2016), which is robust for uneven sampling, implemented in structureselector (Li & Liu, 2018). As a further test of the influence of uneven population sampling, we repeated the analyses with only four individuals from WAU, and with no individuals from WAU. To assess whether or not there was finer scale genetic structure within the Australian populations, we analyzed the EAU samples, with and without a subset of four individuals from WAU using faststructure, under the same conditions as described above. A nested faststructure analysis was also carried out for *K* values of 1–6 on the identified South African populations. Population membership proportions were visualized using pophelper 2.2.7 (Francis, 2017). Principal component analysis (PCA) was conducted with snprelate 1.24.0 (Zheng et al., 2012).

### 2.6 Bayesian modeling of introduction scenarios

We conducted ABC analyses to make inferences about the introduction history of bitou bush from South Africa to EAU, and to WAU. We defined five genetic groups of bitou bush based on the faststructure results, which were supported by the phylogenetic analysis and the geographical information. These groups consisted of three from the native range in South Africa: (1) native range south (NRS: Qholora Mouth, East Beach, and East London), (2) native range central (NRC: Dwesa, Hluleka, Hole in the Wall, and Port St John), (3) native range north (NRN: Durban, Mtunzini, St Lucia, and Tugela Mouth), and two from Australia: (4) eastern Australia (EAU: Dunbogan, Iluka, La Perouse, Minnie Water, Newcastle, Port Kembla, Wollongong, Fraser Island, and Harvey Bay) and (5) Western Australia (WAU: Kwinana; Table 1). We also carried out a secondary analysis using more fine‐scale population structure based on the nested faststructure analysis, which split the individuals into 10 populations based on location, except for Mtunzini and Tugela Mouth, which were clustered together. The prior values for the time between sampling and the invasion of EAU and WAU were drawn from uniform distributions bounded between 38 and 45 generations and 8 and 10 generations, respectively. We assumed an average generation time of 3 years, based on observations of phenology (Scott, 1996; Scott, Batchelor, Jucker, & Webber, 2019), so these prior settings correspond conservatively to the first observations of bitou bush in EAU in 1908 (Weiss et al., 2008) and WAU in 1995 (Scott, Batchelor, Jucker, & Webber, 2019). We included unsampled “ghost” populations (Slatkin, 2005) in our scenarios to address the uncertainty about whether we had sampled the source population(s) of the Australian populations. Further details of parameter settings are provided in Table S4. Loci potentially under selection were removed (see “Supplementary outlier analysis” in Supporting Information).

We used the software DIYABC 2.1.0 (Cornuet et al., 2014) to generate reference tables with summary statistics based on simulated datasets. The whole set of summary statistics available in diyabc were applied, in addition to the linear discriminant analysis (LDA) axes. It was not necessary to use a held out set of test summary statistics, because we used an ABC random forest approach implemented in the R package abcrf 1.8 (Pudlo et al., 2016; Raynal et al., 2019) to select the best‐fitting invasion scenario. This method uses an *out‐of‐bag* error estimate to determine an error rate, which is as accurate as using a test set of the same size as the training set (Pudlo et al., 2016). Following Pudlo et al. (2016), a total of 10,000 simulated datasets per scenario were used. To ensure this number was sufficient, additional analyses with 5000 and 7000 datasets per scenario were carried out (Table S5). We estimated prior error rates for 100, 500, and 1000 trees in the random forest using the *out‐of‐bag* error calculation implemented in *err.abcrf*. Based on the stabilization of prior error rates at 1000 trees, we used this number for our analyses. A sequential approach was applied, comparing subsets of competing scenarios, then comparing the best‐supported scenarios from each subset to determine the best scenario. This approach reduces the number of scenarios that need to be compared, while ensuring that the best scenarios always compete directly. Two preliminary analyses excluding the WAU population were carried out to compare scenarios with a single origin or an admixed origin of the EAU population. The most highly supported scenarios, that is, those receiving more than the average number of votes (trees/number of scenarios), were selected to be tested in a final analysis of the invasion of EAU. The best scenario for the introduction into EAU was used in all further analyses, which included the WAU population. Two preliminary analyses conducted as above identified the best scenarios for the origin of the WAU population considering either an invasion out of South Africa, or an invasion involving the EAU population. The most highly supported scenarios were then selected as above and compared in a final analysis. For the best scenario identified in this final analysis of all populations, 1 million additional simulated datasets were generated with diyabc to use for model checking and parameter estimation. Model checking was conducted with diyabc using 10,000 sets of summary statistics (1%) that were compared with the observed values. To prevent overfitting, an additional model checking analysis was carried out using the set of summary statistics that had not been used for the additional scenario selection step for the final scenario. The posterior distributions of the parameters were estimated by using local linear regression on the 1% of the simulated data closest to our observed data set, with a logit transformation of the parameter values.

### 2.7 Ploidy‐level and genome size estimation

We used two methods to estimate the ploidy‐level and genome size of bitou bush. Sample ploidy was inferred based on allele frequencies at biallelic sites using nquire (Weiß et al., 2018) with a minimum allele coverage of 10 reads and a minor allele frequency of at least 0.2. Noise resulting from mismapping and other effects was scaled down using the *denoise* function and ploidy was inferred with *lrdmodel*, which uses maximum likelihood to identify the most likely fixed ploidy model (diploid, triploid, or tetraploid) compared to a free model that optimally fits the data.

We also inferred the ploidy level and genome size of bitou bush through flow cytometry of well‐watered fresh leaf tissue harvested on the same day as the analysis (Doležel et al., 1989). Nuclei suspensions of bitou bush and the reference plant *Solanum lycopersicum* L. cultivar “Stupické polní rané” (tomato, 2C DNA content = 1.96 pg DNA; Doležel et al., 1992) were prepared using the method of Galbraith et al. (1983). Approximately 2–3 cm^2^ of bitou bush and tomato leaf tissue were chopped separately (single stain, 3 replicates) and together (co‐stain, 5 replicates) with a razor blade in 400 μl of chilled LB‐01 extraction buffer (Doležel et al., 1989) and filtered through a 40 μM nylon filter to remove debris. The filtered suspension was stained using the Cystain® PI Absolute P Kit (Partec GmbH, Münster, Germany) by adding 1600 μl of staining buffer, 5 μl of 3.3 mg/L RNaseA (provided with kit), and adding 1 mg/ml of propidium iodide (PI) to a final concentration of 50 μg/ml (single stain) or 65 μg/ml (co‐stain). Unstained controls were prepared by substituting the PI volume for staining buffer. The stained samples were incubated at 4°C for 2 h in the dark. After incubation, the stained samples were run through a flow cytometer (BD Accuri C6 Plus, BD Bioscience, San Jose, CA, USA) using a 488 nm excitation wavelength with the FL2 detector. To exclude debris signals, the FSC‐H threshold was set to 80,000 and the FL2‐H threshold to 600. Measurements were taken on a fast flow rate (66 μl/min) until 10,000 PI signals were recorded. The data were analyzed in Flowjo™ v10.6.1 (Becton, Dickinson and Company, Ashland, OR, USA) to calculate geometric means and coefficient of variances (CV). Sample 2C DNA content and genome size were calculated as per Doležel and Bartoš (2005).

## 3 RESULTS

### 3.1 Sequencing statistics

Over 1.3 million SNPs were called from a total pool of 143 samples. De novo assembly of the ddRAD loci returned a mean 175,219 loci per sample prior to filtering. The effective per sample mean locus coverage was 22.4× for the bitou‐pisifera‐boneseed data set (SD = 9.6 ×) and 22.3 × (SD = 9.5 ×) for the bitou‐pisifera dataset. After filtering, the number of samples was reduced to 138 and the number of SNPs to 20,221 (average MAF = 0.192).

### 3.2 Population genetic structure

The faststructure analysis of the entire bitou‐boneseed‐pisifera dataset reached an optimal marginal likelihood based on *chooseK* and the method of Puechmaille (2016) at *K* = 4, successfully separating the three subspecies, as well as separating bitou bush individuals from the introduced range in Australia and from the native range in South Africa (Figure S1). When including only bitou bush in the faststructure analysis, the marginal likelihood value reached an optimum at *K* = 5 (Figure S2), indicating that there were five genetic clusters of bitou bush in the analyses (Figure 2, Figure S3). Within Australia, the WAU samples were all assigned to the same cluster with no admixture (green bars, Figure 2). The EAU samples all showed admixture between this cluster and a second cluster (purple bars, Figure 2) that was only present in EAU. The South African bitou bush individuals were separated into three geographically distinct genetic clusters in the faststructure analysis: northern, central, and southern (Figure 2). A nested faststructure analysis of these three South African clusters revealed substructure that was almost completely consistent with the geographical sampling locations (Figures S4–S7). The only exception was that Tugela Mouth and Mtunzini were clustered as a single population. When analyses were conducted with only four individuals from WAU, and with no individuals from WAU to prevent biases due to uneven population sampling, the optimal marginal likelihood was reached at *K* = 3 in both instances. However, the results were similar to the analysis with the full dataset for any given value of *K*. When only EAU populations were included, the optimal marginal likelihood was reached at *K* = 1, although some geographic structure could be detected at higher values of *K* (Figure S8). When WAU individuals were added, the optimal marginal likelihood was reached at *K* = 2, and some similarities could be detected between the individuals from Harvey Bay, Queensland, and the individuals from WAU (Figure S9).

Results from PCA, neighbor‐joining networks, and cladograms are consistent with the results of the faststructure analysis, but also reveal relationships between the genetic clusters. The PCA of bitou bush samples identified five clusters broadly congruent with the faststructure analysis at *K* = 5 (Figure S10). The first axis explained 22.34% of the variance and differentiated Australian samples from South African samples. The second axis explained 6.13% of the variance and separated WAU samples from EAU samples, and separated the South African samples into three clusters. These clusters group samples according to latitude as in the faststructure analysis, although they differ in that the Durban samples cluster separately from the other northern native range populations.

**FIGURE 3:**
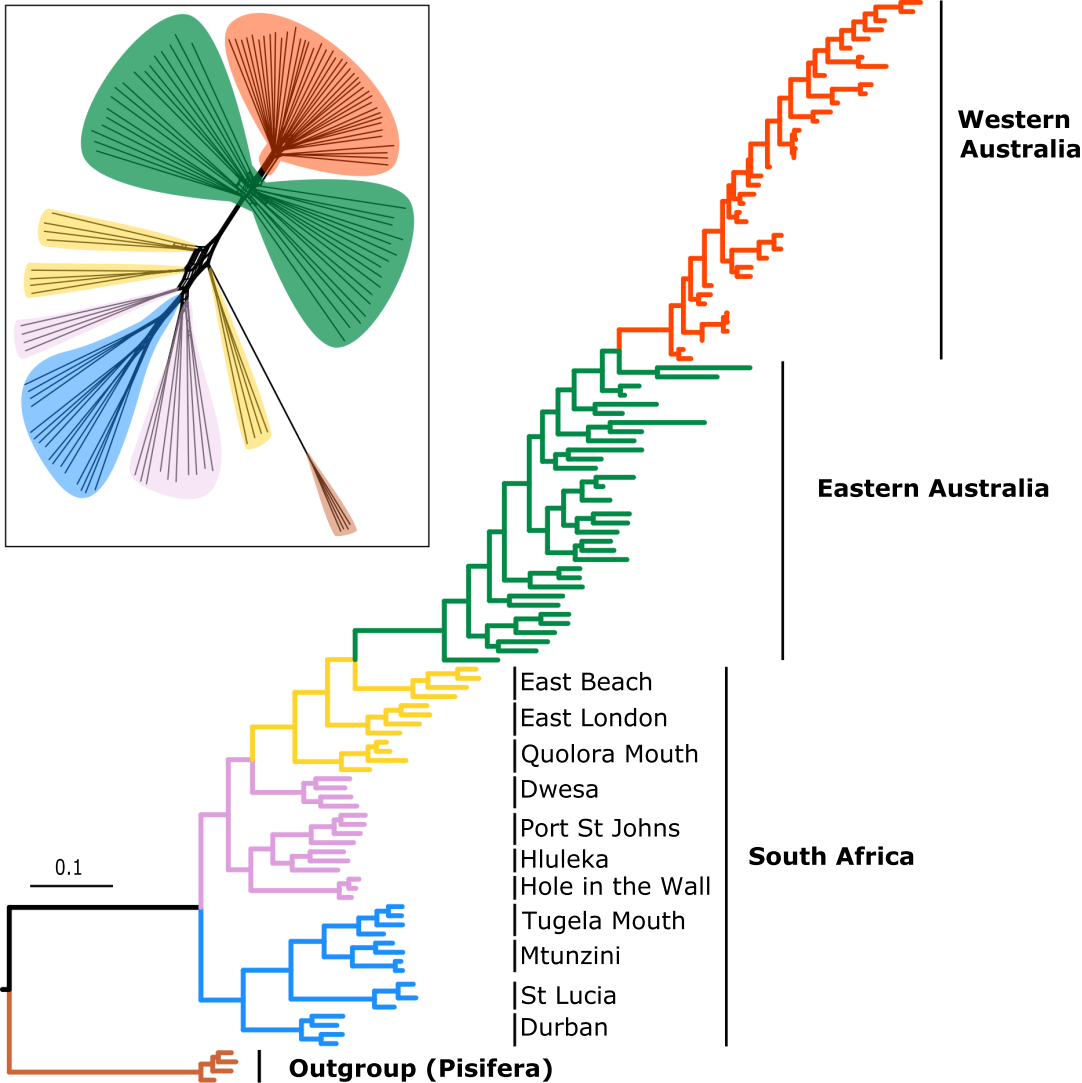
Maximum likelihood phylogeny of *Chrysanthemoides monilifera* ssp. *rotundata* (bitou bush) from South Africa and Australia showing the genetic relationship between all sampled individuals of bitou bush. Branch lengths are scaled to genetic distance. The outgroup is *Chrysanthemoides monilifera* ssp. *pisifera*. The top‐left box shows a supporting splitstree neighbor‐net network analysis based on an identity‐by‐state distance matrix.

The southernmost cluster from South Africa is the most similar to the Australian samples. Neighbor‐net network analysis supported a distinct cluster of the WAU individuals nested within a cluster of the EAU individuals (Figure S11). Based on bootstrap values >70%, the phylogenetic analysis confidently delineated the WAU cluster (Figure 3; Figure S12). Within South Africa, we detected a similar pattern of geographic structure to that observed in the faststructure analysis, but with increased levels of population sub‐division, supported by bootstrap values >70%. Of the South African bitou bush populations, those from the southern‐most populations were the most closely related to the Australian populations.

The results from the faststructure and neighbor‐net network were supported by *Ф*_ST_ population statistics (Table 2). High differentiation was inferred between the South African populations and the WAU (*Ф*_ST_ = 0.256) and EAU (*Ф*_ST_ = 0.189) populations. The WAU population maintained a high level of population differentiation from EAU (*Ф*_ST_ = 0.131), indicating limited, if any, gene flow between populations. The genetic structuring between South Africa and Australia, in addition to the structuring within Australia, was supported by fine scale population *Ф*_ST_ comparisons (Table S6).

**TABLE 2:** *Ф*_ST_ pairwise matrix for the core dataset showing the levels of broad‐scale population differentiation for *Chrysanthemoides monilifera* ssp. *rotundata* (Bitou bush) between Western Australia and eastern Australia (introduced range), and South Africa (native range).

	Eastern Australia	South Africa
Western Australia	0.131	0.256
Eastern Australia		0.189

### 3.3 Population genetic diversity

The South African populations collectively contained the highest number of private alleles, with large reductions of private alleles observed in the Australian populations (Table 3). Of the Australian populations, those from EAU contained considerably more private alleles (160) than the WAU population (3). The number of polymorphic loci was greatest in South Africa (95.30%), with fewer observed in EAU (69.28%) and WAU (48.88%). Nucleotide diversity showed the same trend, decreasing from South Africa to EAU and then WAU. The differences in *H*_*o*_ between most populations were marginal with a range of 0.216–0.269. Inbreeding was negligible in the WAU (*F*_IS_ = −0.057) and EAU (*F*_IS_ = −0.023) populations, with a slight excess of heterozygotes over the expected number in both regions. Increased inbreeding was inferred in the South African populations (*F*_IS_ = 0.278), likely because we are pooling multiple genetically differentiated populations in this calculation. The reduction of private alleles in the Australian populations relative to the South African populations provides evidence of a genetic bottleneck occurring, although most measures of genetic diversity have not been affected.

**TABLE 3:** Population genetic diversity summary statistics and inbreeding coefficient (*F*_IS_) for the broad‐scale populations of *Chrysanthemoides monilifera* ssp. *rotundata* (Bitou bush). Standard error shown for heterozygosity and *F*_IS_ and nucleotide diversity (π).

Population	Private alleles	Polymorphic loci (%)	Observed heterozygosity	Expected heterozygosity	*F*_IS_	π
Western Australia	3	48.88	0.216 ± 0.002	0.183 ± 0.002	−0.057 ± 0.018	0.18513 ± 0.00165
Eastern Australia	160	69.28	0.269 ± 0.002	0.266 ± 0.002	−0.023 ± 0.021	0.27095 ± 0.00161
South Africa	4980	95.30	0.233 ± 0.002	0.318 ± 0.001	0.278 ± 0.040	0.32262 ± 0.00118

### 3.4 Bayesian modeling of introduction scenarios

The ABC random forest analysis supported the scenario that EAU bitou bush populations originated from an unsampled ghost population (posterior probability = 0.52; Tables S7 and S8). Further, the analysis suggested that the WAU population originated via admixture between this ghost population and the EAU population, which could have occurred postintroduction to EAU (posterior probability = 0.49; Tables 4 and S9). The outcome of the final scenario was supported by a re‐analysis using a larger dataset of 4000 SNPs (Table S10). Parameter estimation showed strong bottlenecks associated with the invasion of EAU (mean effective size of founder population = 8; 5%–95% quantiles = 5.0–13.4) and WAU (mean effective size of founder population = 9; 5%–95% quantiles = 5.0–11.1), with both estimations close to the minimum bound of 5 (Table S11). Narrow priors were set for the times of the invasion and the estimated parameters did not further narrow down the times, showing confidence intervals corresponding to the priors for both EAU (38–45 generations) and WAU (8–10 generations). Model checking showed a discrepancy between the observed and the simulated summary statistics for the final scenario (Figure S13), with 51 of 100 summary statistics significantly differing between the datasets and 11 of 25 significantly differing for the subset of summary statistics not used for model selection (Table S12). Relaxing the priors on population sizes or divergence times did not improve the model fit (not shown), suggesting that a complex demographic history led to the incompletely sampled observed populations analyzed in this study. To ensure that substructure in the South African populations did not impact the main results of the ABC modeling, the analysis was repeated after splitting the South African samples into 10 populations based on the nested faststructure analysis. The source of the EAU population was once again inferred as a ghost population (posterior probability = 0.55, Tables S13–S15). Unlike in the modeling based on three South African populations, the WAU population was recovered as originating from a second ghost population (posterior probability = 0.49). This inferred scenario received the second highest number of ABC random forest votes in the three‐population analysis. A total of 349 out of 576 simulated summary statistics for the final scenario showed significant deviations from the observed values.

**TABLE 4:** Results of model choice analysis using ABC random forest, for *Chrysanthemoides monilifera* ssp. *rotundata* (Bitou bush) introduction to Western Australia. Population acronyms are as defined in Table 1.

Source population	Admixed with	Bottleneck	RF votes (of 1000)^a^	Posterior probability
GHOST1	–	Y	207	
GHOST2	–	Y	66	
NRN	NRS	Y	89	
NRS	NRC	Y	65	
EAU	GHOST1	Y	379	0.49
EAU	NRS	Y	194	

^a^ Refers to the number of times each modeled scenario was selected in 1000 simulations.

### 3.5 Ploidy and genome size evolution

**FIGURE 4:**
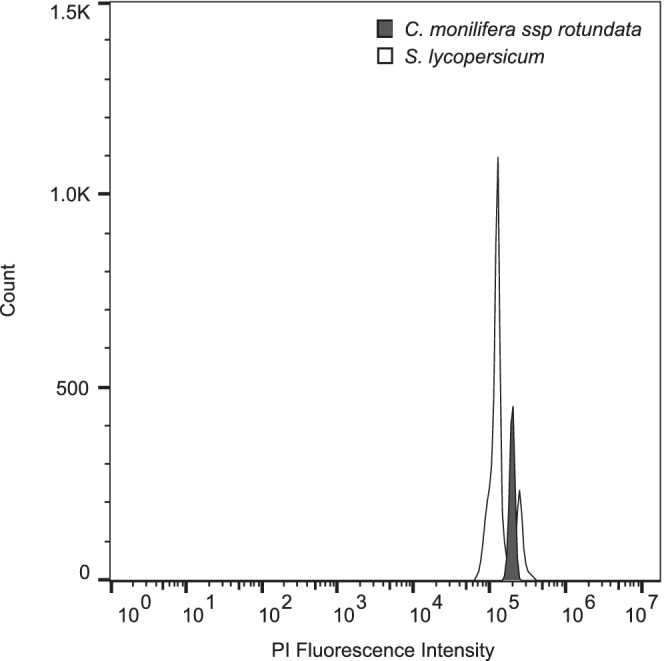
Smoothed fluorescence histograms showing the estimation of nuclear DNA content (2C) and ploidy for *Chrysanthemoides monilifera* ssp. *rotundata* (bitou bush) using *Solanum lycopersicum* (tomato) cultivar “Stupické polní rané” as a reference standard.

Ploidy‐level inference from nquire analyses found no significant deviations from expected base frequency distributions under diploidy for any samples, although ploidy of 27 samples was inferred as ambiguous (Table S16). The results of the flow cytometry indicate that the sampled individuals were DNA diploids with only a single G1 peak observed in each replicate (Figure 4; Figure S14). The 2C DNA content of bitou bush is estimated to be 3.11 ± 0.01 pg with a 1C genome size of 1519 ± 4 Mbp (Table S17).

## 4 DISCUSSION

In this study, we sought to determine the population genetic diversity and structure of the coastal shrub, bitou bush, and to reconstruct the species' introduction history and invasion dynamics in Australia using SNPs called from ddRADseq. Through these insights, we aimed to assess the likely effectiveness of current management strategies for bitou bush in Australia, and demonstrate how new genomics methods can improve invasion management outcomes for the South African‐Australian exchange of weeds, and biological invasions more broadly. We found genetic differentiation among populations across the native range in South Africa, and differentiation between South African and Australian populations. We detected a moderate reduction in genetic diversity following introduction, with a reduction in the number of private alleles following introduction into EAU and again into WAU, but with no reduction in heterozygosity and no inbreeding. Results of flow cytometry and population genetics are consistent with Australian bitou bush invasions comprising diploid, outcrossing populations.

None of the sampled populations were inferred to be the most likely source population for the Australian introduction. Instead, we hypothesized that an unsampled “ghost” population in the native range was the most likely source for the introduction of bitou bush into EAU. However, phylogenetic analysis shows that the introduced bitou bush are more closely related to populations toward the southern end of the range, rather than those toward the northern end of the range. The introduction was associated with a moderate genetic bottleneck, implying a limited number of founding individuals from the same native population. Subsequent introductions to WAU occurred with the EAU bitou bush serving as a “bridgehead” population, with evidence for a further genetic bottleneck. These results show that successful biological invasions can occur despite strong bottlenecks and reductions in allelic richness, and provide some support for the role of bridgehead populations as a mechanism for invasion success. These results have implications for the management of bitou bush and other South African Australian introductions in their non‐native ranges, and for the management of invasive species more broadly, and these will be discussed below.

### 4.1 Genetic structure and diversity

Genetic structure and diversity were consistent with recognized subspecies, and with previous phylogenetic analyses based on ISSR markers, ITS2 and DNA barcode sequencing, showing a closer relationship between bitou bush and pisifera, relative to boneseed (Barker et al., 2009; Barker et al., 2015). Within bitou bush, our SNP data showed further geographic structure within the South African range. The WAU and EAU populations of bitou bush were distinct from each other and from all South African populations. This was apparent in genetic clustering (faststructure and PCA) and phylogenetic analyses. Within South Africa, we detected three distinct genetic clusters in bitou bush of northern, central, and southern populations through faststructure analysis. The same clusters were detected with PCA, but with the northern cluster further subdivided into samples from Durban and samples from the populations further north. Each South African population formed a clade in phylogenetic analysis, with groupings consistent with the faststructure and PCA analyses. Within each of the Australian regions there was little, if any, geographic structure. A lack of genetic structure is likely the result of relatively recent population expansion from a single introduction and/or high ongoing gene flow. Both explanations would be consistent with the intentional planting of material sourced from a limited number of plants for dune stabilization across a broad geographic range (Weiss et al., 2008), with subsequent further spread through seeds from these plants.

The Australian bitou bush populations exhibited a reduction in private alleles and polymorphic loci relative to the South African populations, which are symptomatic of a genetic bottleneck occurring during founding events (Greenbaum et al., 2014). Often non‐native species are found to have low genetic diversity at neutral loci relative to native populations, suggesting that high genetic diversity is not necessary for a successful invasion (e.g., Alexander et al., 2009; Hardesty et al., 2012; Hirsch et al., 2021). This can be the case even when multiple introductions have occurred (e.g., Zhu et al., 2017). Despite the decrease in bitou bush genetic diversity in the non‐native range relative to the native range, there was no evidence of heterozygosity loss or inbreeding in the WAU and EAU populations, with a slight excess heterozygosity detected in both regions. There are other examples where a genetic bottleneck during introduction has not led to an increase in inbreeding (e.g., *Miconia calvescens*, Hardesty et al., 2012; *Acacia dealbata*, Hirsch et al., 2019), and increased outcrossing in the introduced range can even increase heterozygosity through recombination of genotypes that are geographically separated in the native range (e.g., Alexander et al., 2009). In bitou bush, only a single introduction to Australia was inferred, so the excess heterozygosity cannot be explained by recombination of different native range genotypes. The excess heterozygosity may be the result of small effective population size in a species that is likely to be obligately outcrossed and self‐incompatible (Gross et al., 2017). The ability of non‐native species to thrive and adapt to new environments postgenetic bottleneck has been described as the genetic paradox of invasion (Estoup et al., 2016; Sax & Brown, 2000). We found that bitou bush has experienced a genetic bottleneck resulting in reduced genetic variation and it has survived this bottleneck without succumbing to problems associated with low genetic variation, although given the short timeframe of the WAU invasion, these effects may not have had time to arise. Our study has not tested whether bitou bush is adapting to its introduced range, and it is possible that the source population was already well‐suited to the conditions in the introduced range.

We did not find evidence of polyploidy in bitou bush or related subspecies from base frequencies at biallelic SNPs or flow cytometry, although other researchers have detected varying numbers of chromosomes (Table S18). Many invasive species are polyploid, with polyploidy providing a range of benefits in a colonizing population (Baker, 1974; te Beest et al., 2012). It is therefore essential to rule out the possibility of polyploidy before undertaking any genetic analysis of an invasive plant species to avoid erroneous results. No individuals of bitou bush or related subspecies from any population had within‐individual allele frequencies deviating from expectations under diploidy. Individuals within the WAU population were inferred to be DNA diploids using flow cytometry and are estimated to have a 2C DNA content of 3.11 ± 0.01 pg. In the closely related genus *Calendula*, flow cytometry and chromosome counts have detected diploid (2C DNA content of 1.75–3.47 pg) and tetraploid (2C DNA content of 2.97–5.41 pg) species (Nora et al., 2013). Our inference of ploidy and genome size should be further tested with chromosome counts to confirm that bitou bush is a true diploid (Doležel et al., 2007).

### 4.2 Introduction history

Bayesian modeling inferred introduction to Australia from an unsampled “ghost” population, likely with a strong bottleneck, albeit with only moderate posterior probability. We are not aware of any non‐native bitou bush populations outside of Australia, so we infer that the source population for Australian bitou bush invasions is either an unsampled population in the native range, or is not represented by any extant population. Bitou bush populations in South Africa do exist to the north and south west along the coastline beyond our sampling extent (Barker et al., 2015). It is therefore possible that they may be the source of the seeds for the first Australian introductions. Alternatively, it may be that the original population from which the Australian populations are descended is now extinct, or that bitou bush populations in Australia and/or South Africa have diverged since the introduction event, as a result of either natural selection or genetic drift. Ghost populations have also been inferred for the introduction of invasive *Acacia dealbata*, even with comprehensive sampling of the native range, with similar explanations for this result (Hirsch et al., 2019; Hirsch et al., 2021).

Further sampling in South Africa may reveal the source population, or at least further refine the close relationship between the Australian populations and the populations from the southern part of the native range. Of the sampled South African populations, the southwestern‐most sampled population, East Beach, near Port Alfred in the Eastern Cape province of South Africa, is the most closely related to the Australian populations. The introduction pathway has been assumed to be through dry ballast from shipping in the 19th and 20th centuries (Weiss et al., 2008). However, no conclusive historical evidence has been presented to support this contention. Ports in the bitou bush native range that were shipping internationally at that time included Durban, East London, and Gqeberha (formerly Port Elizabeth). The phylogenetic analysis and PCA from this work show that the Durban population is genetically divergent from the Australian populations and an unlikely source population. Prioritizing samples from other port areas in future molecular studies would help to test the hypothesis that dry ballast was the most likely introduction pathway to Australia.

Based on the strong bottleneck, decreased genetic diversity, and lack of genetic structure in the Australian range, we can infer that the bitou bush used for dune stabilization along the coast of New South Wales was most likely sourced from local plants, rather than from additional imports from South Africa, and that these plants were the source of further spread into Queensland. Our results also provide evidence that the EAU population served as a source population for the introduction into WAU, with genetic clustering of these populations supported by faststructure, pca and phylogenetic analysis. We note, however, that there was also support from Bayesian analysis for a scenario where the WAU population was derived from an unsampled “ghost” population. The EAU populations are so similar that introduction from a specific location is difficult to discern. It is possible that the source population for the WAU introduction was not sampled in this study, as our sampling in EAU was only able to access material from populations between Wollongong and southern Queensland. This sampled range left approximately 500 km of coastline unsampled between Wollongong and the southern limits of established bitou bush populations near Mallacoota, Victoria. While many of these populations have been locally extirpated, if herbarium specimens were taken before extirpation, they could play a role in future research to further refine the introduction history of bitou bush using methods such as genome skimming that are less sensitive to DNA degradation. It is likely that bitou bush was anthropogenically introduced to WAU, since the population is centered around an industrial port that had historical connections to EAU (Scott & Batchelor, 2014).

Similar secondary invasions have been documented in other non‐native plants, including *Centaurea solstitialis* L. (Barker et al., 2017; Eriksen et al., 2014) and *Ambrosia artemisiifolia* L. (van Boheemen et al., 2017). In these examples, the populations have evolved over centuries following the initial introduction, creating a bridgehead population that has adapted to local conditions, providing a fitter source population for secondary invasions. In the case of *C. solstitialis*, it was concluded that increased plant size has evolved following introduction (Barker et al., 2017). In contrast, bitou bush has been present in EAU only since the early 20th century (Weiss et al., 2008) and in WAU since ca. 1995 (Scott & Batchelor, 2014), representing a shorter period where the plant could adapt to local conditions prior to the secondary invasion. Rapid evolution of non‐native plants is known to occur in <20 generations, resulting in strong genetic differentiation and differences in phenotype between native and introduced populations (Prentis et al., 2008; Turner et al., 2014). Our study found that non‐native bitou bush populations had diverged from the native populations at neutral loci following introduction, but did not directly address whether adaptation to local conditions was occurring. The plants in WAU are exceptionally large compared to those in the native range, but this is likely the result of less pressure from specialist herbivores and diseases (i.e., enemy release hypothesis), rather than heritable trait changes (Scott, Batchelor, Jucker, & Webber, 2019). However, this is also a positive indicator for the potential to improve management by implementing classical biological control.

### 4.3 Invasion management implications

The increased understanding of introduction history, spread, and genetic diversity of bitou bush across its Australian range provides four insights into how to refine the different management strategies being applied to this weed across Australia. First, from an introduction perspective, we were able to confirm that the EAU invasion originated from a small founding population from the native range in South Africa, and that subsequent EAU dune stabilization plantings were most likely carried out using locally sourced propagules, rather than new introductions from one or more locations in the native range. We can also infer that the invasion process is unlikely to be exacerbated by ongoing gene flow to WAU and EAU populations from the native range. Given that the WAU population likely has its origins from an accidental EAU introduction, we caution that interstate dispersal remains a risk within Australia. Western Australia's high border quarantine standards therefore need to be maintained, particularly from areas where bitou bush propagules could be a contaminant on imported goods. In turn, prioritizing localized extirpation around larger EAU ports would be a priority to mitigate risk at the source.

Second, up to now searches for biological control agents for bitou bush in its native range have not been guided by genetic information on the origin of the introduction. Our data points to an Eastern Cape source for bitou bush. The initial biological control studies (Adair & Scott, 1989) sourced insects (the geometrid *Comostolopsis germana* Prout) from the Port of Durban toward the northern end of the native range, to meet climate matching criteria with bitou infested areas of EAU and on the assumption that Durban was the most likely port of origin (Scott unpublished observations). While *C. germana* is a successful agent, many others have failed (Adair et al., 2012; Adair & Scott, 1991), indicating that the nexus between agent, host species and source should be re‐examined with a focus on this newly identified region of origin.

Third, from a population invasion perspective, our findings help inform management in contrasting ways between WAU and EAU. In WAU, bitou bush is early in its invasion history, has a small population size, and studies on an EAU population show that it is an obligate outcrosser (Gross et al., 2017), which could make WAU bitou bush particularly susceptible to Allee effects. This situation also implies that invasion events must consist of at least two seeds that successfully germinate and grow to reproductive age, within cross pollination distance and are present as adult plants at the same time. All these factors are likely to combine to make reintroduction unlikely and eradication of bitou bush in WAU a realistic and feasible management goal (Scott, Batchelor, & Webber, 2019). New plants in the WAU population are likely to come from the seed bank, rather than dispersal from the EAU population. The seed bank of bitou bush is estimated to persist for no more than 8 years and will determine the time required for eradication (Scott, Batchelor, & Webber, 2019). In contrast in EAU, our insight reveals that genetics is not as useful for guiding optimal management choices. The low genetic structure observed across populations is most likely due to the extensive nature of past dune stabilization plantings locally sourced from the same population, and low genetic diversity within EAU suggests that the source material had limited genetic variation. Such relative uniformity makes it more challenging to use molecular insight to help inform dispersal and connectivity parameters, which in turn can guide where to position containment lines and target extirpation (Adair & Butler, 2010; Behrendorff et al., 2019; Cherry et al., 2008).

For informing the broader biotic exchange between South Africa and Australia, our findings on bitou bush support the application of new genomic tools to the most problematic of invasive species under management. There is the likelihood of identifying more profitable regions for targeting biological control agent searches, particularly for target species that are distributed over the broad climatic gradients that occur in these two countries. There is also a real chance of proactively mitigating future biosecurity risks by better characterizing the pathways and propagule pressure of past introductions, given that sea and air links between the countries remain particularly strong. Finally, there is merit in considering future work that would identify suites of species with similar traits that could be tackled together to take advantage of a greater efficiency for undertaking the primary research, as well as for implementing improved management plans. Two immediate examples include combining a further focus on bitou bush with boneseed, and to undertake a combined research effort on the suite of Australian acacias that have been introduced into South Africa (noting that substantial work has already been done on the latter; Jansen & Kumschick, 2021; Magona et al., 2018; Richardson et al., 2011).

### 4.4 Conclusions and future research directions

By utilizing a ddRADseq approach, we have traced the introduction history of the non‐native invasive coastal plant, bitou bush, from South Africa into EAU, then to WAU, and determined that successful invasion occurred despite strong bottlenecks. Our research has revealed new knowledge on the introduction history of bitou bush, which can be applied to optimizing management. Prior to conducting genetic analysis, the source population of Australian bitou bush invasions was expected to be near Durban, and hence, searches for biocontrol agents were focussed in this area (Adair & Scott, 1989, 1991). Our study was unable to identify a specific native range source population for Australian bitou bush invasions, although there was some evidence for a source population toward the southern limits of the distribution in South Africa. To further understand the introduction history of bitou bush, more intense sampling is required at the southern limit of its natural distribution. Sampling of herbarium specimens may help determine if the source population is extinct or if there has been genetic divergence postinvasion. Increased understanding of introduction history could improve future biocontrol efforts.

Finally, our research on bitou bush has highlighted the importance of genetic data for understanding invasion risk and fine‐tuning management. We have identified introduction pathways that were previously unknown, showing that bitou bush management may benefit from further research on biological control given these new findings. This demonstrates the importance of understanding invasion history and the role of genetic analysis as an essential tool in fully understanding biological invasions, and incorporating this information into evidence‐based management and prevention of biological invasions.

## AUTHOR CONTRIBUTIONS

**Dennis Byrne:** Conceptualization (supporting); formal analysis (supporting); investigation (lead); project administration (equal); writing – original draft (lead); writing – review and editing (supporting). **Armin Scheben:** Data curation (equal); formal analysis (lead); methodology (supporting); software (lead); writing – original draft (supporting); writing – review and editing (supporting). **John K. Scott:** Conceptualization (lead); funding acquisition (supporting); methodology (supporting); supervision (supporting); writing – original draft (supporting); writing – review and editing (supporting). **Bruce L. Webber:** Conceptualization (supporting); funding acquisition (lead); methodology (supporting); supervision (supporting); writing – original draft (supporting); writing – review and editing (supporting). **Kathryn L. Batchelor:** Investigation (supporting); project administration (supporting). **Anita Severn‐Ellis:** Investigation (supporting); methodology (supporting); writing – original draft (supporting). **Ben Gooden:** Conceptualization (supporting); investigation (supporting); writing – original draft (supporting); writing – review and editing (supporting). **Karen Bell:** Conceptualization (supporting); data curation (lead); funding acquisition (supporting); investigation (supporting); methodology (lead); project administration (supporting); supervision (lead); writing – original draft (supporting); writing – review and editing (lead).

## CONFLICT OF INTEREST

The authors declare no conflict of interest.

## Supporting information


Appendix S1

## Data Availability

Genomic data used for this project are available at NCBI under bioproject PRJNA525912. Scripts used in bioinformatics are available at https://github.com/ascheben/bitou_analysis.
