# Supplementary material for: Genomics reveals the history of a complex plant invasion and improves the management of a biological invasion from the South African–Australian biotic exchange
Source: Ecol Evol. 2022 Aug 23;12(8):e9179. doi: 10.1002/ece3.9179 (PMC9396708; doi:10.1002/ece3.9179)
Supplement: Supplementary file 1 — Appendix S1 [file ECE3-12-e9179-s001.docx]

Supplementary material for:

**Genomics reveals the history of a complex plant invasion and improves the management of a biological invasion from the South African-Australian biotic exchange**

Dennis Byrne^1,2,a^, Armin Scheben^2,a,b^, John K. Scott^1,2^, Bruce L. Webber^1,2,3^, Kathryn L. Batchelor^1^, Anita A. Severn-Ellis^2^, Ben Gooden^4,5^, Karen L. Bell^1,2,*^

^1^ CSIRO Health & Biosecurity, 147 Underwood Ave, Floreat, Western Australia 6014, Australia

^2^ School of Biological Sciences, University of Western Australia, 35 Stirling Highway, Crawley, Western Australia 6009, Australia

^3^ Western Australian Biodiversity Science Institute, 133 St Georges Terrace, Perth, Western Australia 6000, Australia

^4^ CSIRO Health and Biosecurity, GPO Box 1700, Canberra, ACT 2601, Australia

^5^ Centre for Sustainable Ecosystem Solutions, School of Earth, Atmospheric and Life Sciences, University of Wollongong, Wollongong, 2522, New South Wales, Australia

^a^ The first two authors contributed equally

^b^ Current address: Simons Center for Quantitative Biology, Cold Spring Harbor Laboratory, Cold Spring Harbor, NY 11724, USA

**^*^Correspondence.** Email [Karen.Bell@csiro.au](mailto:Karen.Bell@csiro.au)

**Table S1:** Sample names, identifiers, sampling co-ordinates, collection details, and herbarium voucher details for *Chrysanthemoides monilifera* (bitou bush and related subspecies) samples used in this study

| **Sample** | **Collector(s)** | **Collector’s sample number** | **Latitude** | **Longitude** | **Location description** | **Subspecies** | **Herbarium voucher** |
| --- | --- | --- | --- | --- | --- | --- | --- |
| WAU_KWI12_BB_107 | J. K. Scott, K. L. Batchelor, & D. Byrne | KWIN'12 107 | -32.21173 | 115.76888 | Kwinana, WA | bitou bush | NA |
| WAU_KWI12_BB_11 | J. K. Scott, K. L. Batchelor, & D. Byrne | KWIN'12 11 | -32.21173 | 115.76888 | Kwinana, WA | bitou bush | NA |
| WAU_KWI12_BB_12 | J. K. Scott, K. L. Batchelor, & D. Byrne | KWIN'12 12 | -32.21173 | 115.76888 | Kwinana, WA | bitou bush | NA |
| WAU_KWI12_BB_150 | J. K. Scott, K. L. Batchelor, & D. Byrne | KWIN'12 150 | -32.21173 | 115.76888 | Kwinana, WA | bitou bush | NA |
| WAU_KWI12_BB_200 | J. K. Scott, K. L. Batchelor, & D. Byrne | KWIN'12 200 | -32.21173 | 115.76888 | Kwinana, WA | bitou bush | NA |
| WAU_KWI12_BB_226 | J. K. Scott, K. L. Batchelor, & D. Byrne | KWIN'12 226 | -32.21173 | 115.76888 | Kwinana, WA | bitou bush | NA |
| WAU_KWI12_BB_249 | J. K. Scott, K. L. Batchelor, & D. Byrne | KWIN'12 249 | -32.21173 | 115.76888 | Kwinana, WA | bitou bush | NA |
| WAU_KWI12_BB_277 | J. K. Scott, K. L. Batchelor, & D. Byrne | KWIN'12 277 | -32.21173 | 115.76888 | Kwinana, WA | bitou bush | NA |
| WAU_KWI12_BB_348 | J. K. Scott, K. L. Batchelor, & D. Byrne | KWIN'12 348 | -32.21173 | 115.76888 | Kwinana, WA | bitou bush | NA |
| WAU_KWI12_BB_410 | J. K. Scott, K. L. Batchelor, & D. Byrne | KWIN'12 410 | -32.21173 | 115.76888 | Kwinana, WA | bitou bush | NA |
| WAU_KWI12_BB_414 | J. K. Scott, K. L. Batchelor, & D. Byrne | KWIN'12 414 | -32.21173 | 115.76888 | Kwinana, WA | bitou bush | NA |
| WAU_KWI12_BB_415 | J. K. Scott, K. L. Batchelor, & D. Byrne | KWIN'12 415 | -32.21173 | 115.76888 | Kwinana, WA | bitou bush | NA |
| WAU_KWI12_BB_416 | J. K. Scott, K. L. Batchelor, & D. Byrne | KWIN'12 416 | -32.21173 | 115.76888 | Kwinana, WA | bitou bush | NA |
| WAU_KWI12_BB_510 | J. K. Scott, K. L. Batchelor, & D. Byrne | KWIN'12 510 | -32.21173 | 115.76888 | Kwinana, WA | bitou bush | NA |
| WAU_KWI12_BB_512 | J. K. Scott, K. L. Batchelor, & D. Byrne | KWIN'12 512 | -32.21173 | 115.76888 | Kwinana, WA | bitou bush | NA |
| WAU_KWI12_BB_565 | J. K. Scott, K. L. Batchelor, & D. Byrne | KWIN'12 565 | -32.21173 | 115.76888 | Kwinana, WA | bitou bush | NA |
| WAU_KWI12_BB_620 | J. K. Scott, K. L. Batchelor, & D. Byrne | KWIN'12 620 | -32.21173 | 115.76888 | Kwinana, WA | bitou bush | NA |
| WAU_KWI12_BB_621 | J. K. Scott, K. L. Batchelor, & D. Byrne | KWIN'12 621 | -32.21173 | 115.76888 | Kwinana, WA | bitou bush | NA |
| WAU_KWI12_BB_625 | J. K. Scott, K. L. Batchelor, & D. Byrne | KWIN'12 625 | -32.21173 | 115.76888 | Kwinana, WA | bitou bush | NA |
| WAU_KWI12_BB_626 | J. K. Scott, K. L. Batchelor, & D. Byrne | KWIN'12 626 | -32.21173 | 115.76888 | Kwinana, WA | bitou bush | NA |
| WAU_KWI18_BB_01 | J. K. Scott, K. L. Batchelor, & D. Byrne | KWIN 1 | -32.21173 | 115.76888 | Kwinana, WA | bitou bush | NA |
| WAU_KWI18_BB_02 | J. K. Scott, K. L. Batchelor, & D. Byrne | KWIN 10 | -32.21173 | 115.76888 | Kwinana, WA | bitou bush | NA |
| WAU_KWI18_BB_03 | J. K. Scott, K. L. Batchelor, & D. Byrne | KWIN 14 | -32.21173 | 115.76888 | Kwinana, WA | bitou bush | NA |
| WAU_KWI18_BB_04 | J. K. Scott, K. L. Batchelor, & D. Byrne | KWIN 17 | -32.21173 | 115.76888 | Kwinana, WA | bitou bush | NA |
| WAU_KWI18_BB_05 | J. K. Scott, K. L. Batchelor, & D. Byrne | KWIN 19 | -32.21173 | 115.76888 | Kwinana, WA | bitou bush | NA |
| WAU_KWI18_BB_06 | J. K. Scott, K. L. Batchelor, & D. Byrne | KWIN 2 | -32.21173 | 115.76888 | Kwinana, WA | bitou bush | NA |
| WAU_KWI18_BB_07 | J. K. Scott, K. L. Batchelor, & D. Byrne | KWIN 22 | -32.21173 | 115.76888 | Kwinana, WA | bitou bush | NA |
| WAU_KWI18_BB_08 | J. K. Scott, K. L. Batchelor, & D. Byrne | KWIN 23 | -32.21173 | 115.76888 | Kwinana, WA | bitou bush | NA |
| WAU_KWI18_BB_09 | J. K. Scott, K. L. Batchelor, & D. Byrne | KWIN 24 | -32.21173 | 115.76888 | Kwinana, WA | bitou bush | NA |
| WAU_KWI18_BB_10 | J. K. Scott, K. L. Batchelor, & D. Byrne | KWIN 25 | -32.21173 | 115.76888 | Kwinana, WA | bitou bush | NA |
| WAU_KWI18_BB_11 | J. K. Scott, K. L. Batchelor, & D. Byrne | KWIN 3 | -32.21173 | 115.76888 | Kwinana, WA | bitou bush | NA |
| WAU_KWI18_BB_12 | J. K. Scott, K. L. Batchelor, & D. Byrne | KWIN 4 | -32.21173 | 115.76888 | Kwinana, WA | bitou bush | NA |
| WAU_KWI18_BB_13 | J. K. Scott, K. L. Batchelor, & D. Byrne | KWIN 6 | -32.21173 | 115.76888 | Kwinana, WA | bitou bush | NA |
| WAU_KWI18_BB_14 | J. K. Scott, K. L. Batchelor, & D. Byrne | KWIN 7 | -32.21173 | 115.76888 | Kwinana, WA | bitou bush | NA |
| WAU_KWI18_BB_15 | J. K. Scott & K. L. Batchelor | 2 | -32.21173 | 115.76888 | Kwinana, WA | bitou bush | NA |
| WAU_KWI18_BB_16 | J. K. Scott & K. L. Batchelor | 3 | -32.21173 | 115.76888 | Kwinana, WA | bitou bush | NA |
| WAU_KWI18_BB_17 | J. K. Scott & K. L. Batchelor | 31 | -32.21173 | 115.76888 | Kwinana, WA | bitou bush | NA |
| WAU_KWI18_BB_18 | J. K. Scott & K. L. Batchelor | 32 | -32.21173 | 115.76888 | Kwinana, WA | bitou bush | NA |
| WAU_KWI18_BB_19 | J. K. Scott & K. L. Batchelor | 57 | -32.21173 | 115.76888 | Kwinana, WA | bitou bush | NA |
| WAU_KWI18_BB_20 | J. K. Scott & K. L. Batchelor | 58 | -32.21173 | 115.76888 | Kwinana, WA | bitou bush | NA |
| WAU_ROL_BS_01 | J. K. Scott & K. L. Batchelor | BS 1 | -32.11828 | 116.09258 | Roleystone, WA | boneseed | NA |
| WAU_ROL_BS_02 | J. K. Scott & K. L. Batchelor | BS 2.0 | -32.11828 | 116.09258 | Roleystone, WA | boneseed | NA |
| WAU_ROL_BS_03 | J. K. Scott & K. L. Batchelor | BS 4.0 | -32.11828 | 116.09258 | Roleystone, WA | boneseed | NA |
| WAU_ROL_BS_04 | J. K. Scott & K. L. Batchelor | BS 5 | -32.11828 | 116.09258 | Roleystone, WA | boneseed | NA |
| WAU_ROL_BS_05 | J. K. Scott & K. L. Batchelor | BS 7 | -32.11828 | 116.09258 | Roleystone, WA | boneseed | NA |
| WAU_ROL_BS_06 | J. K. Scott & K. L. Batchelor | BS 9 | -32.11828 | 116.09258 | Roleystone, WA | boneseed | NA |
| EAU_NSW_DUN_BB_01 | P. Michael | PMHC 1 | -31.64785 | 152.83488 | Dunbogan, NSW | boneseed | NA |
| EAU_NSW_DUN_BB_02 | P. Michael | PMHC 2 | -31.64785 | 152.83488 | Dunbogan, NSW | bitou bush | NA |
| EAU_NSW_DUN_BB_03 | P. Michael | PMHC 3 | -31.64785 | 152.83488 | Dunbogan, NSW | bitou bush | NA |
| EAU_NSW_ILU_BB_01 | R. Luxton | RL-ILUKA 1 | -29.41826 | 153.36190 | Iluka, NSW | bitou bush | NA |
| EAU_NSW_ILU_BB_02 | R. Luxton | RL-ILUKA 2 | -29.41826 | 153.36190 | Iluka, NSW | bitou bush | NA |
| EAU_NSW_ILU_BB_03 | R. Luxton | RL-ILUKA 3 | -29.41826 | 153.36190 | Iluka, NSW | bitou bush | NA |
| EAU_NSW_KEM_BB_01 | B. Gooden | MAR1 | -34.48578 | 150.91676 | Port Kembla, NSW | bitou bush | NA |
| EAU_NSW_KEM_BB_02 | B. Gooden | MAR2 | -34.48578 | 150.91676 | Port Kembla, NSW | bitou bush | NA |
| EAU_NSW_KEM_BB_03 | B. Gooden | MAR3 | -34.48578 | 150.91676 | Port Kembla, NSW | bitou bush | NA |
| EAU_NSW_KEM_BB_04 | B. Gooden | OP1 | -34.48578 | 150.91676 | Port Kembla, NSW | bitou bush | NA |
| EAU_NSW_KEM_BB_05 | B. Gooden | OP2 | -34.48578 | 150.91676 | Port Kembla, NSW | bitou bush | NA |
| EAU_NSW_KEM_BB_06 | B. Gooden | OP3 | -34.48578 | 150.91676 | Port Kembla, NSW | bitou bush | NA |
| EAU_NSW_LAP_BB_01 | K. Waterhouse | KW 2 | -33.98877 | 151.23434 | La Perouse, NSW | bitou bush | NA |
| EAU_NSW_LAP_BB_02 | K. Waterhouse | KW 3 | -33.98877 | 151.23434 | La Perouse, NSW | bitou bush | NA |
| EAU_NSW_LAP_BB_03 | K. Waterhouse | KW1 | -33.98877 | 151.23434 | La Perouse, NSW | bitou bush | NA |
| EAU_NSW_LAP_BB_04 | K. Waterhouse | KW4 | -33.98877 | 151.23434 | La Perouse, NSW | bitou bush | NA |
| EAU_NSW_MIN_BB_01 | R. Luxton | RL-MINNIE 1 | -29.78263 | 153.29690 | Minnie Water, NSW | bitou bush | NA |
| EAU_NSW_MIN_BB_02 | R. Luxton | RL-MINNIE 2 | -29.78263 | 153.29690 | Minnie Water, NSW | bitou bush | NA |
| EAU_NSW_MIN_BB_03 | R. Luxton | RL-MINNIE 3 | -29.78263 | 153.29690 | Minnie Water, NSW | bitou bush | NA |
| EAU_NSW_NEW_BB_01 | R. Armstrong | RA1 | -32.91650 | 151.78449 | Newcastle, NSW | bitou bush | NA |
| EAU_NSW_NEW_BB_02 | R. Armstrong | RA2 | -32.91650 | 151.78449 | Newcastle, NSW | bitou bush | NA |
| EAU_NSW_NEW_BB_03 | R. Armstrong | RA3 | -32.91650 | 151.78449 | Newcastle, NSW | bitou bush | NA |
| EAU_NSW_WOL_BB_02 | B. Gooden | BGBATH 2 | -34.40844 | 150.90181 | Wollongong, NSW | bitou bush | NA |
| EAU_NSW_WOL_BB_03 | B. Gooden | BGBATH 3 | -34.40844 | 150.90181 | Wollongong, NSW | bitou bush | NA |
| EAU_NSW_WOL_BB_04 | B. Gooden | BGBATH4 | -34.40844 | 150.90181 | Wollongong, NSW | bitou bush | NA |
| EAU_NSW_WOL_BB_06 | B. Gooden | WG2 | -34.40844 | 150.90181 | Wollongong, NSW | bitou bush | NA |
| EAU_NSW_WOL_BB_07 | B. Gooden | WG3 | -34.40844 | 150.90181 | Wollongong, NSW | bitou bush | NA |
| EAU_QLD_FRA_BB_01 | H. Haapakoski & S. Harris | SH01 | -25.74976 | 153.08785 | Fraser Island, QLD | bitou bush | NA |
| EAU_QLD_FRA_BB_02 | H. Haapakoski & S. Harris | SH02 | -25.74976 | 153.08785 | Fraser Island, QLD | bitou bush | NA |
| EAU_QLD_FRA_BB_03 | H. Haapakoski & S. Harris | SH04 | -25.74976 | 153.08785 | Fraser Island, QLD | bitou bush | NA |
| EAU_QLD_HAR_BB_01 | H. Haapakoski & S. Harris | ENT1 | -27.43648 | 153.53917 | Harvey Bay, QLD | bitou bush | NA |
| EAU_QLD_HAR_BB_02 | H. Haapakoski & S. Harris | NOTH1 | -27.43648 | 153.53917 | Harvey Bay, QLD | bitou bush | NA |
| EAU_QLD_HAR_BB_03 | H. Haapakoski & S. Harris | NOTH2 | -27.43648 | 153.53917 | Harvey Bay, QLD | bitou bush | NA |
| EAU_VIC_ART_BS_01 | A. Johnson | AJ1 | -38.36753 | 144.93783 | Arthur's Seat, VIC | boneseed | NA |
| EAU_VIC_ART_BS_02 | A. Johnson | AJ2 | -38.36753 | 144.93783 | Arthur's Seat, VIC | boneseed | NA |
| EAU_VIC_ART_BS_03 | A. Johnson | AJ3 | -38.36753 | 144.93783 | Arthur's Seat, VIC | boneseed | NA |
| EAU_VIC_ART_BS_04 | R. Cousens | RC02 | -38.36753 | 144.93783 | Arthur's Seat, VIC | boneseed | NA |
| EAU_VIC_ART_BS_05 | R. Cousens | RC03 | -38.36753 | 144.93783 | Arthur's Seat, VIC | boneseed | NA |
| EAU_VIC_ELT_BS_01 | R. Cousens | RCO1 | -37.69457 | 145.17272 | Eltham Aqueduct, VIC | boneseed | NA |
| EAU_VIC_ELT_BS_02 | R. Cousens | RCO4 | -37.69457 | 145.17272 | Eltham Aqueduct, VIC | boneseed | NA |
| EAU_VIC_FAP_BS_01 | R. Cousens | RCO5 | -37.78782 | 145.01469 | Fairfield Park, VIC | boneseed | NA |
| EAU_VIC_FAP_BS_02 | R. Cousens | RCO7 | -37.78782 | 145.01469 | Fairfield Park, VIC | boneseed | NA |
| EAU_VIC_FAP_BS_03 | R. Cousens | RCO8 | -37.78782 | 145.01469 | Fairfield Park, VIC | boneseed | NA |
| EAU_VIC_FLI_BS_01 | A. Johnson | AJ4 | -38.48017 | 145.00919 | Flinders Coastline, VIC | boneseed | NA |
| EAU_VIC_FLI_BS_02 | A. Johnson | AJ5 | -38.48017 | 145.00919 | Flinders Coastline, VIC | boneseed | NA |
| EAU_VIC_FLI_BS_03 | A. Johnson | AJ6 | -38.48017 | 145.00919 | Flinders Coastline, VIC | boneseed | NA |
| RSA_DUR_BB_01 | V. R. Clark | DURB1 | -29.90195 | 31.040277 | Durban, South Africa | bitou bush | NA |
| RSA_DUR_BB_02 | V. R. Clark | DURB2 | -29.90195 | 31.040277 | Durban, South Africa | bitou bush | NA |
| RSA_DUR_BB_03 | V. R. Clark | DURB3 | -29.90195 | 31.040277 | Durban, South Africa | bitou bush | NA |
| RSA_DUR_BB_04 | V. R. Clark | DURB4 | -29.90195 | 31.040277 | Durban, South Africa | bitou bush | NA |
| RSA_DWE_BB_01 | V. R. Clark & T. Te Water Naude | DW1 | -32.30477 | 28.832007 | Dwesa, South Africa | bitou bush | NA |
| RSA_DWE_BB_02 | V. R. Clark & T. Te Water Naude | DW2 | -32.30477 | 28.832007 | Dwesa, South Africa | bitou bush | NA |
| RSA_DWE_BB_03 | V. R. Clark & T. Te Water Naude | DW3 | -32.30477 | 28.832007 | Dwesa, South Africa | bitou bush | NA |
| RSA_DWE_BB_04 | V. R. Clark & T. Te Water Naude | DW4 | -32.30477 | 28.832007 | Dwesa, South Africa | bitou bush | NA |
| RSA_EBE_BB_01 | V. R. Clark | EB1 | -33.59782 | 26.90060 | East Beach, South Africa | bitou bush | VR Clark East Beach 1 (GRA) |
| RSA_EBE_BB_02 | V. R. Clark | SA1 | -33.59782 | 26.90060 | East Beach, South Africa | bitou bush | VR Clark East Beach 2 (GRA) |
| RSA_EBE_BB_03 | V. R. Clark | SA2 | -33.59782 | 26.90060 | East Beach, South Africa | bitou bush | VR Clark East Beach 3 (GRA) |
| RSA_EBE_BB_04 | V. R. Clark | EB2 | -33.59782 | 26.90060 | East Beach, South Africa | bitou bush | VR Clark East Beach 4 (GRA) |
| RSA_ELD_BB_01 | V. R. Clark & T. Te Water Naude | EL1 | -33.03252 | 27.911188 | East London, South Africa | bitou bush | NA |
| RSA_ELD_BB_02 | V. R. Clark & T. Te Water Naude | EL2 | -33.03252 | 27.911188 | East London, South Africa | bitou bush | NA |
| RSA_ELD_BB_03 | V. R. Clark & T. Te Water Naude | EL3 | -33.03252 | 27.911188 | East London, South Africa | bitou bush | NA |
| RSA_ELD_BB_04 | V. R. Clark & T. Te Water Naude | EL4 | -33.03252 | 27.911188 | East London, South Africa | bitou bush | NA |
| RSA_FAI_PISI_01 | V. R. Clark | SA5 | -33.32689 | 26.55322 | Fairewood, South Africa | pisifera | VR Clark Fairewood 1 (GRA) |
| RSA_FAI_PISI_02 | V. R. Clark | SA6 | -33.32689 | 26.55322 | Fairewood, South Africa | pisifera | VR Clark Fairewood 2 (GRA) |
| RSA_FAI_PISI_03 | V. R. Clark | SA7 | -33.32689 | 26.55322 | Fairewood, South Africa | pisifera | VR Clark Fairewood 3 (GRA) |
| RSA_FAI_PISI_04 | V. R. Clark | SA8 | -33.32689 | 26.55322 | Fairewood, South Africa | pisifera | VR Clark Fairewood 4 (GRA) |
| RSA_HLU_BB_01 | V. R. Clark & T. Te Water Naude | HLU1 | -31.82777 | 29.302506 | Hluleka, South Africa | bitou bush | NA |
| RSA_HLU_BB_02 | V. R. Clark & T. Te Water Naude | HLU2 | -31.82777 | 29.302506 | Hluleka, South Africa | bitou bush | NA |
| RSA_HLU_BB_03 | V. R. Clark & T. Te Water Naude | HLU3 | -31.82777 | 29.302506 | Hluleka, South Africa | bitou bush | NA |
| RSA_HOL_BB_02 | V. R. Clark & T. Te Water Naude | HW2 | -32.03973 | 29.106107 | Hole in the wall, South Africa | bitou bush | NA |
| RSA_HOL_BB_03 | V. R. Clark & T. Te Water Naude | HW3 | -32.03973 | 29.106107 | Hole in the wall, South Africa | bitou bush | NA |
| RSA_HOL_BB_04 | V. R. Clark & T. Te Water Naude | HW4 | -32.03973 | 29.106107 | Hole in the wall, South Africa | bitou bush | NA |
| RSA_MZN_BB_01 | V. R. Clark | MZN1 | -28.95778 | 31.763052 | Mtunzini, South Africa | bitou bush | VR Clark KZN3 (NU0092464) |
| RSA_MZN_BB_02 | V. R. Clark | MZN2 | -28.95778 | 31.763052 | Mtunzini, South Africa | bitou bush | NA |
| RSA_MZN_BB_03 | V. R. Clark | MZN3 | -28.95778 | 31.763052 | Mtunzini, South Africa | bitou bush | NA |
| RSA_MZN_BB_04 | V. R. Clark | MZN4 | -28.95778 | 31.763052 | Mtunzini, South Africa | bitou bush | NA |
| RSA_QMO_BB_01 | V. R. Clark & T. Te Water Naude | WC2.1 | -32.6405 | 28.429565 | Qholora Mouth, South Africa | bitou bush | NA |
| RSA_QMO_BB_02 | V. R. Clark & T. Te Water Naude | WC2.2 | -32.6405 | 28.429565 | Qholora Mouth, South Africa | bitou bush | NA |
| RSA_QMO_BB_03 | V. R. Clark & T. Te Water Naude | WC2.3 | -32.6405 | 28.429565 | Qholora Mouth, South Africa | bitou bush | NA |
| RSA_QMO_BB_04 | V. R. Clark & T. Te Water Naude | WC2.4 | -32.6405 | 28.429565 | Qholora Mouth, South Africa | bitou bush | NA |
| RSA_STJ_BB_01 | V. R. Clark & T. Te Water Naude | SJN3 | -31.62387 | 29.547416 | Port St Johns, South Africa | bitou bush | NA |
| RSA_STJ_BB_02 | V. R. Clark & T. Te Water Naude | SJN4 | -31.62387 | 29.547416 | Port St Johns, South Africa | bitou bush | NA |
| RSA_STJ_BB_03 | V. R. Clark & T. Te Water Naude | SJN5 | -31.62387 | 29.547416 | Port St Johns, South Africa | bitou bush | NA |
| RSA_STJ_BB_04 | V. R. Clark & T. Te Water Naude | SJN2 | -31.62387 | 29.547416 | Port St Johns, South Africa | bitou bush | NA |
| RSA_STL_BB_01 | V. R. Clark | SL1 | -28.3625 | 32.432496 | St Lucia, South Africa | bitou bush | VR Clark KZN4 (NU0092463) |
| RSA_STL_BB_02 | V. R. Clark | SL2 | -28.3625 | 32.432496 | St Lucia, South Africa | bitou bush | NA |
| RSA_STL_BB_03 | V. R. Clark | SL3 | -28.3625 | 32.432496 | St Lucia, South Africa | bitou bush | NA |
| RSA_STL_BB_04 | V. R. Clark | SL4 | -28.3625 | 32.432496 | St Lucia, South Africa | bitou bush | NA |
| RSA_TMO_BB_01 | V. R. Clark | TM1 | -29.22083 | 31.501111 | Tugela Mouth, South Africa | bitou bush | VR Clark KZN2 (NU0092462) |
| RSA_TMO_BB_02 | V. R. Clark | TM2 | -29.22083 | 31.501111 | Tugela Mouth, South Africa | bitou bush | NA |
| RSA_TMO_BB_03 | V. R. Clark | TM3 | -29.22083 | 31.501111 | Tugela Mouth, South Africa | bitou bush | NA |
| RSA_TMO_BB_04 | V. R. Clark | TM4 | -29.22083 | 31.501111 | Tugela Mouth, South Africa | bitou bush | NA |

**Table S2:** Number of reads retained per sample through processing steps of quality filtering, adapter removal and checking for the RAD enzyme recognition site (CGT), and the final mean Phred quality score for each sample of *Chrysanthemoides monilifera* (bitou bush and relatives) used in this study.

| **Sample** | **Total raw** r**eads** | **Low quality reads** | **Retained reads** | **Reads after adapter removal** | **Reads after RAD check** | **Final mean base phred score** |
| --- | --- | --- | --- | --- | --- | --- |
| WAU_KWI12_BB_249 | 10498902 | 108764 | 10390138 | 9963410 | 1247385 | 36 |
| WAU_KWI12_BB_416 | 11573510 | 120013 | 11453497 | 11357193 | 11067995 | 35 |
| WAU_KWI12_BB_626 | 9196013 | 95643 | 9100370 | 8882905 | 8634806 | 36 |
| EAU_NSW_WOL_BB_02 | 10057593 | 106075 | 9951518 | 9382798 | 9169493 | 37 |
| EAU_NSW_WOL_BB_03 | 8166801 | 82815 | 8083986 | 7295091 | 7100616 | 38 |
| WAU_KWI18_BB_01 | 7668083 | 77219 | 7590864 | 7536921 | 7262613 | 39 |
| EAU_NSW_DUN_BB_02 | 11634161 | 117876 | 11516285 | 11036469 | 10858797 | 38 |
| EAU_NSW_DUN_BB_03 | 8494999 | 86324 | 8408675 | 7895340 | 7756343 | 39 |
| EAU_NSW_ILU_BB_02 | 7634589 | 79114 | 7555475 | 6880159 | 5720302 | 39 |
| EAU_NSW_ILU_BB_03 | 11880312 | 124235 | 11756077 | 10792369 | 10582064 | 37 |
| EAU_NSW_MIN_BB_01 | 14323908 | 145120 | 14178788 | 13492945 | 7881633 | 37 |
| EAU_NSW_MIN_BB_02 | 7532872 | 76370 | 7456502 | 6852546 | 6718261 | 39 |
| WAU_ROL_BS_05 | 11956081 | 120039 | 11836042 | 11346868 | 6086798 | 39 |
| WAU_ROL_BS_06 | 15282156 | 153627 | 15128529 | 14778949 | 8788836 | 39 |
| WAU_KWI18_BB_15 | 7631035 | 77672 | 7553363 | 7446925 | 7150423 | 36 |
| WAU_KWI18_BB_11 | 6382473 | 59782 | 6322691 | 6305740 | 5967348 | 38 |
| WAU_KWI18_BB_17 | 4449650 | 43062 | 4406588 | 4371791 | 4090582 | 37 |
| WAU_KWI18_BB_18 | 2727466 | 25750 | 2701716 | 2689685 | 2603614 | 38 |
| WAU_KWI18_BB_02 | 6135980 | 58700 | 6077280 | 6060485 | 5954352 | 37 |
| WAU_KWI18_BB_03 | 4686904 | 45173 | 4641731 | 4633646 | 4474297 | 36 |
| WAU_KWI18_BB_04 | 10746785 | 103544 | 10643241 | 10614574 | 10401178 | 37 |
| WAU_KWI18_BB_05 | 7131820 | 68703 | 7063117 | 7027797 | 6894429 | 37 |
| WAU_KWI18_BB_07 | 6530764 | 62305 | 6468459 | 6455189 | 6252902 | 38 |
| WAU_KWI18_BB_08 | 5377746 | 50845 | 5326901 | 5316279 | 5010258 | 37 |
| WAU_KWI18_BB_10 | 6065447 | 59560 | 6005887 | 5984545 | 5811504 | 38 |
| WAU_KWI18_BB_16 | 7511703 | 76314 | 7435389 | 7310514 | 7146540 | 38 |
| EAU_NSW_WOL_BB_04 | 7150843 | 67585 | 7083258 | 7047133 | 6852980 | 37 |
| EAU_NSW_DUN_BB_01 | 4772730 | 46086 | 4726644 | 4685414 | 4547759 | 39 |
| EAU_NSW_ILU_BB_01 | 14268751 | 136009 | 14132742 | 14089670 | 5771405 | 39 |
| EAU_NSW_MIN_BB_03 | 9136863 | 87177 | 9049686 | 8953590 | 8674967 | 39 |
| EAU_NSW_LAP_BB_01 | 15503611 | 145376 | 15358235 | 15236480 | 7987821 | 39 |
| WAU_ROL_BS_01 | 7148384 | 69978 | 7078406 | 7054515 | 6763136 | 37 |
| WAU_KWI12_BB_107 | 6914330 | 68617 | 6845713 | 6774884 | 6621446 | 35 |
| WAU_KWI12_BB_150 | 9546269 | 90779 | 9455490 | 9406866 | 9209375 | 35 |
| WAU_KWI12_BB_226 | 6126033 | 57576 | 6068457 | 6024521 | 5894790 | 35 |
| WAU_KWI12_BB_410 | 8601814 | 84326 | 8517488 | 8477867 | 8235019 | 36 |
| WAU_KWI12_BB_415 | 11465964 | 109869 | 11356095 | 11325868 | 4734522 | 36 |
| WAU_KWI12_BB_512 | 6078502 | 58204 | 6020298 | 5952295 | 5824885 | 36 |
| WAU_KWI12_BB_625 | 10662105 | 97588 | 10564517 | 10537255 | 4090504 | 35 |
| WAU_KWI18_BB_19 | 9222266 | 78572 | 9143694 | 8934502 | 8660369 | 37 |
| WAU_KWI18_BB_20 | 6958719 | 60483 | 6898236 | 6124746 | 6057527 | 38 |
| EAU_NSW_LAP_BB_02 | 5894087 | 51900 | 5842187 | 5585621 | 5511447 | 38 |
| WAU_KWI18_BB_06 | 4497735 | 42304 | 4455431 | 4404579 | 4191232 | 37 |
| WAU_ROL_BS_04 | 4483421 | 38638 | 4444783 | 3801164 | 3603559 | 39 |
| WAU_KWI12_BB_348 | 6745894 | 56513 | 6689381 | 10468026 | 5868410 | 36 |
| WAU_KWI12_BB_565 | 6939263 | 58580 | 6880683 | 6807793 | 6322666 | 36 |
| WAU_KWI12_BB_621 | 5726417 | 47661 | 5678756 | 5479743 | 5294633 | 36 |
| EAU_VIC_ART_BS_04 | 6336145 | 56066 | 6280079 | 6162379 | 5996271 | 37 |
| EAU_VIC_ART_BS_05 | 7096970 | 62654 | 7034316 | 6896683 | 6693848 | 39 |
| EAU_QLD_HAR_BB_01 | 16522362 | 143118 | 16379244 | 16091767 | 15442640 | 36 |
| EAU_QLD_HAR_BB_02 | 7525259 | 65110 | 7460149 | 7262416 | 7130910 | 37 |
| WAU_KWI18_BB_09 | 7554900 | 78119 | 7476781 | 7433622 | 7022894 | 37 |
| EAU_QLD_HAR_BB_03 | 27127194 | 239989 | 26887205 | 26089488 | 22105561 | 38 |
| EAU_QLD_FRA_BB_01 | 7105393 | 62026 | 7043367 | 6938320 | 6801618 | 39 |
| EAU_QLD_FRA_BB_02 | 9371672 | 79481 | 9292191 | 9095387 | 8865538 | 38 |
| EAU_QLD_FRA_BB_03 | 3945152 | 34061 | 3911091 | 3839272 | 3747040 | 37 |
| EAU_NSW_NEW_BB_01 | 12974182 | 110885 | 12863297 | 12768101 | 8423482 | 37 |
| EAU_NSW_NEW_BB_02 | 6183501 | 49991 | 6133510 | 5996040 | 5853394 | 37 |
| EAU_NSW_NEW_BB_03 | 13212090 | 124056 | 13088034 | 12999948 | 5892022 | 40 |
| WAU_ROL_BS_02 | 5197671 | 50634 | 5147037 | 5091993 | 4986237 | 38 |
| WAU_ROL_BS_03 | 6552824 | 61028 | 6491796 | 6395634 | 6289740 | 38 |
| WAU_KWI18_BB_12 | 3220028 | 30216 | 3189812 | 3185615 | 3083964 | 39 |
| RSA_EBE_BB_02 | 9725002 | 79838 | 9645164 | 9614083 | 6836347 | 37 |
| RSA_EBE_BB_03 | 13097804 | 113556 | 12984248 | 12927213 | 12644234 | 37 |
| RSA_FAI_PISI_01 | 6489005 | 54647 | 6434358 | 6340839 | 6191037 | 39 |
| RSA_FAI_PISI_02 | 11783666 | 100099 | 11683567 | 11480105 | 11236937 | 39 |
| RSA_FAI_PISI_03 | 6848197 | 57701 | 6790496 | 6674971 | 6409750 | 41 |
| RSA_FAI_PISI_04 | 10074351 | 81943 | 9992408 | 9916182 | 4892538 | 40 |
| WAU_KWI18_BB_13 | 9164320 | 92777 | 9071543 | 8967988 | 8801415 | 40 |
| WAU_KWI18_BB_14 | 9333217 | 96013 | 9237204 | 9168417 | 9004559 | 37 |
| WAU_KWI12_BB_11 | 6415337 | 5632 | 6409705 | 6029627 | 5698051 | 36 |
| WAU_KWI12_BB_12 | 6688787 | 5931 | 6682856 | 6536377 | 6251017 | 36 |
| WAU_KWI12_BB_200 | 6435161 | 5840 | 6429321 | 6283357 | 5717659 | 34 |
| WAU_KWI12_BB_277 | 7208439 | 6396 | 7202043 | 6970924 | 6377103 | 35 |
| WAU_KWI12_BB_414 | 6133462 | 5485 | 6127977 | 6006356 | 5455024 | 33 |
| WAU_KWI12_BB_510 | 7038048 | 6160 | 7031888 | 6621917 | 6086635 | 34 |
| WAU_KWI12_BB_620 | 3737538 | 3335 | 3734203 | 3295929 | 1968458 | 34 |
| EAU_VIC_FLI_BS_01 | 6358626 | 5704 | 6352922 | 6316099 | 5533234 | 38 |
| EAU_VIC_FLI_BS_02 | 5371803 | 4657 | 5367146 | 5315032 | 4905957 | 38 |
| EAU_VIC_FLI_BS_03 | 4737801 | 4133 | 4733668 | 4698512 | 4412448 | 38 |
| EAU_VIC_ART_BS_01 | 6141123 | 5398 | 6135725 | 6030078 | 5482516 | 38 |
| EAU_VIC_ART_BS_02 | 5850071 | 5162 | 5844909 | 5803325 | 5396945 | 38 |
| EAU_VIC_ART_BS_03 | 6958750 | 6009 | 6952741 | 6909989 | 6087145 | 37 |
| RSA_DUR_BB_01 | 7887546 | 6571 | 7880975 | 7786222 | 5208445 | 39 |
| RSA_DUR_BB_02 | 7392863 | 6265 | 7386598 | 7248338 | 6514137 | 38 |
| RSA_DUR_BB_03 | 7027579 | 5990 | 7021589 | 6952448 | 6513953 | 35 |
| RSA_DUR_BB_04 | 8636081 | 7446 | 8628635 | 8454235 | 7603243 | 40 |
| RSA_DWE_BB_01 | 7397446 | 6123 | 7391323 | 7271599 | 6398643 | 37 |
| RSA_DWE_BB_02 | 7179920 | 6308 | 7173612 | 6989061 | 5160979 | 37 |
| RSA_DWE_BB_03 | 6923958 | 6117 | 6917841 | 6831299 | 4247905 | 36 |
| RSA_DWE_BB_04 | 5822284 | 5054 | 5817230 | 5693338 | 3704467 | 39 |
| RSA_EBE_BB_01 | 5508482 | 4739 | 5503743 | 5228745 | 3948635 | 41 |
| RSA_EBE_BB_04 | 5887845 | 5146 | 5882699 | 5827293 | 5198092 | 36 |
| RSA_ELD_BB_01 | 5221040 | 4442 | 5216598 | 5198705 | 3802554 | 37 |
| RSA_ELD_BB_02 | 6060130 | 5072 | 6055058 | 5987106 | 5387101 | 39 |
| RSA_ELD_BB_03 | 5594375 | 4676 | 5589699 | 5550031 | 4948935 | 34 |
| RSA_ELD_BB_04 | 5683740 | 4941 | 5678799 | 5628497 | 5295434 | 36 |
| RSA_HLU_BB_01 | 7623885 | 6343 | 7617542 | 7522840 | 6583046 | 37 |
| RSA_HLU_BB_02 | 7094094 | 6192 | 7087902 | 6987164 | 5486977 | 44 |
| RSA_HLU_BB_03 | 5580728 | 4756 | 5575972 | 5532498 | 2927860 | 39 |
| RSA_HOL_BB_02 | 13541320 | 11604 | 13529716 | 12996661 | 11751846 | 37 |
| RSA_HOL_BB_03 | 7682670 | 6665 | 7676005 | 7445716 | 6428032 | 40 |
| RSA_HOL_BB_04 | 9698286 | 8204 | 9690082 | 9381154 | 8403959 | 35 |
| EAU_NSW_LAP_BB_03 | 5115792 | 4585 | 5111207 | 5075108 | 3973652 | 36 |
| EAU_NSW_LAP_BB_04 | 7762960 | 6805 | 7756155 | 7629166 | 6858236 | 37 |
| EAU_NSW_KEM_BB_01 | 4746374 | 4209 | 4742165 | 4693687 | 4368003 | 35 |
| EAU_NSW_KEM_BB_02 | 5788637 | 5007 | 5783630 | 5722048 | 5190796 | 36 |
| EAU_NSW_KEM_BB_03 | 5537921 | 4789 | 5533132 | 5452335 | 4880467 | 35 |
| RSA_MZN_BB_01 | 6975334 | 5986 | 6969348 | 6885060 | 5977990 | 36 |
| RSA_MZN_BB_02 | 6437379 | 5464 | 6431915 | 6373256 | 5910509 | 35 |
| RSA_MZN_BB_03 | 6266569 | 5311 | 6261258 | 6158311 | 5277737 | 35 |
| RSA_MZN_BB_04 | 6730829 | 5625 | 6725204 | 6603376 | 5988602 | 36 |
| EAU_NSW_KEM_BB_04 | 7302309 | 6411 | 7295898 | 7240787 | 6362296 | 36 |
| EAU_NSW_KEM_BB_05 | 5618628 | 4904 | 5613724 | 5538169 | 4938513 | 38 |
| EAU_NSW_KEM_BB_06 | 6207629 | 5540 | 6202089 | 6108671 | 5312416 | 37 |
| EAU_VIC_ELT_BS_01 | 4780271 | 4255 | 4776016 | 4724460 | 4406792 | 38 |
| EAU_VIC_ELT_BS_02 | 5734855 | 5037 | 5729818 | 5664553 | 5144669 | 37 |
| EAU_VIC_FAP_BS_01 | 4880587 | 4309 | 4876278 | 4834306 | 4496575 | 38 |
| EAU_VIC_FAP_BS_02 | 5110373 | 4420 | 5105953 | 5075925 | 4577077 | 37 |
| EAU_VIC_FAP_BS_03 | 5467953 | 4863 | 5463090 | 5415663 | 4952274 | 38 |
| RSA_STJ_BB_04 | 7099045 | 6325 | 7092720 | 6887897 | 5330714 | 40 |
| RSA_STJ_BB_01 | 5867345 | 5031 | 5862314 | 5697865 | 5050160 | 48 |
| RSA_STJ_BB_02 | 5912572 | 5077 | 5907495 | 5814613 | 4874628 | 36 |
| RSA_STJ_BB_03 | 6834864 | 5950 | 6828914 | 6600789 | 5637818 | 39 |
| RSA_STL_BB_01 | 4989500 | 4263 | 4985237 | 4942901 | 4364091 | 36 |
| RSA_STL_BB_02 | 3684183 | 2952 | 3681231 | 3601174 | 2920827 | 37 |
| RSA_STL_BB_03 | 5872471 | 4904 | 5867567 | 5790401 | 4909759 | 35 |
| RSA_STL_BB_04 | 4077416 | 3446 | 4073970 | 3990030 | 3105087 | 35 |
| RSA_TMO_BB_01 | 5941256 | 5009 | 5936247 | 5898001 | 4029418 | 35 |
| RSA_TMO_BB_02 | 6286956 | 5259 | 6281697 | 6221145 | 5382143 | 36 |
| RSA_TMO_BB_03 | 5172920 | 4355 | 5168565 | 5140314 | 3613071 | 36 |
| RSA_TMO_BB_04 | 8244569 | 7051 | 8237518 | 8189718 | 5239658 | 37 |
| RSA_QMO_BB_01 | 7524990 | 6533 | 7518457 | 7432983 | 6412533 | 36 |
| RSA_QMO_BB_02 | 6037129 | 5240 | 6031889 | 5975219 | 3696603 | 35 |
| RSA_QMO_BB_03 | 6014195 | 5155 | 6009040 | 5985106 | 4107052 | 35 |
| RSA_QMO_BB_04 | 7207358 | 6123 | 7201235 | 7122536 | 5046418 | 37 |
| EAU_NSW_WOL_BB_06 | 5261491 | 4547 | 5256944 | 5197900 | 4669076 | 35 |
| EAU_NSW_WOL_BB_07 | 5013662 | 4438 | 5009224 | 4887858 | 4465997 | 36 |

**Table S3:** Parameters used for *de novo* assembly of RAD loci and SNP calling of ddRAD sequence data using stacks 2.1. Separate analyses were conducted for *Chrysanthemoides monilifera* ssp. *rotundata* (bitou bush) together with its close relative *C. monilifera* ssp. *pisifera* (pisifera), for *C. monilifera* ssp. *monilifera* (boneseed), and for all three subspecies together.

| **Group** | **Assembly** | **Parameter (-m, -M, -n)** | **High quality SNPs** | **Effective mean per sample coverage** |
| --- | --- | --- | --- | --- |
| Boneseed | Split | 2 | 2772 | 17.4 |
| Boneseed | Together | 3 | 2930 | 22.2 |
| Boneseed | Split | 3 | 2940 | 17.5 |
| Boneseed | Together | 4 | 3053 | 22.2 |
| Boneseed | Together | 5 | 3106 | 22.2 |
| Bitou-Pisifera | Split | 2 | 16827 | 22.9 |
| Bitou-Pisifera | Together | 3 | 16520 | 22.2 |
| Bitou-Pisifera | Split | 3 | 16623 | 22.9 |
| Bitou-Pisifera | Together | 4 | 16484 | 22.2 |
| Bitou-Pisifera | Together | 5 | 16178 | 22.2 |
| Bitou-Pisifera-Boneseed | Together | 3 | 16819 | 22.2 |
| Bitou-Pisifera-Boneseed | Together | 4 | 16668 | 22.2 |
| Bitou-Pisifera-Boneseed | Together | 5 | 16208 | 22.2 |

**Table S4.** Prior distributions of DIYABC parameters including effective population size (N_e_) and divergence time for the final analysis, used for modelling of introduction history of *Chrysanthemoides monilifera* ssp. *rotundata* (bitou bush) into Australia. Time is shown in generations (1 generation = 3 years). Divergence times were constrained using the condition: t_anc_ > t_invE_ > t_adm_ > t_invW_. The same parameters were used for all preliminary analyses; however for scenarios excluding the Western Australian population we set the condition: t_anc_ > t_adm_ > t_invE_.

| **Parameter** | **Description** | **Prior (uniform distribution)** |
| --- | --- | --- |
| EAU | N_e_ of eastern Australian population | 10-10,000 |
| NRN | N_e_ of native range north population (centred on Durban) | 10-10,000 |
| NRS | N_e_ of native range south population (centred on East Beach) | 10-10,000 |
| NRC | N_e_ of native range central population (centred on Port St. John) | 10-10,000 |
| WAU | N_e_ of Western Australian population | 10-10,000 |
| RSA_GHOST1_ | N_e_ of South African ghost population 1 | 10-10,000 |
| RSA_GHOST2_ | N_e_ of South African ghost population 2 | 10-10,000 |
| RSA | N_e_ of South African ancestral population | 10-10,000 |
| WAU0 | N_e_ of bottlenecked Western Australian population | 5-1,000 |
| EAU0 | N_e_ of bottlenecked eastern Australian population | 5-1,000 |
| t_anc_ | Divergence time from ancestral population | 10-10,000 |
| t_invE_ | Divergence time of invasive eastern Australian population | 38-45 |
| t_invW_ | Divergence time of invasive Western Australian population | 8-10 |
| t_adm_ | Admixture time for admixed populations | 9-44 |
| db1 | Bottleneck time for eastern Australian population | 1-5 |
| db2 | Bottleneck time for Western Australian population | 1-5 |
| ra | Admixture rate between admixed populations | 0.001-0.5 |

**Table S5**. Supporting analysis of the final scenario selection analysis, modelling a subset of scenarios for the introduction of *Chrysanthemoides monilifera* ssp. *rotundata* (bitou bush) into Western Australia, using a range of simulated input datasets (5k: 5,000 SNPs; 7k: 7,000 SNPs; 10k: 10,000 SNPs) with all summary statistics or a subset of 84 summary statistics, excluding the mean genetic diversity, Nei’s distance, *F_ST_*, and admixture statistic (84stats).

|  |  |  | **RF votes (of 1000)** | | | |  |
| --- | --- | --- | --- | --- | --- | --- | --- |
| **Source population** | **Admixed with** | **Bottleneck** | **5k** | **7k** | **10k** | **10k, 84 stats** | **Posterior probability (5k;7k;10k;84stats)** |
| GHOST1 | - | Y | 154 | 180 | 207 | 209 |  |
| GHOST2 | - | Y | 77 | 93 | 66 | 92 |  |
| NRN | NRS | Y | 193 | 136 | 89 | 94 |  |
| NRS | NRC | Y | 65 | 62 | 65 | 38 |  |
| EAU | GHOST1 | Y | 340 | 314 | 379 | 344 | (0.45, 0.46, 0.49, 0.45) |
| EAU | NRS | Y | 171 | 215 | 194 | 223 |  |

**Table S6.** *Ф*_ST_ matrix for the full dataset showing the levels of fine-scale population differentiation between the individual populations of *Chrysanthemoides monilifera* sampled across South Africa and Australia. Populations are *Chrysanthemoides monilifera* ssp. *rotundata* (bitou bush) unless specified. BS = *Chrysanthemoides monilifera* ssp. *monilifera* (boneseed); PISI = *Chrysanthemoides monilifera* ssp. *pisifera*

|  |  | Western Australia | | Eastern Australia | | | | | | | | | | | | | South Africa - East Beach group | | | | South Africa - Port St Johns group | | | | South Africa – Durban group | | | |
| --- | --- | --- | --- | --- | --- | --- | --- | --- | --- | --- | --- | --- | --- | --- | --- | --- | --- | --- | --- | --- | --- | --- | --- | --- | --- | --- | --- | --- |
|  |  | Kwinana (2018) | Roleystone (BS) | Dunbogan | Iluka | La Perouse | Minnie Water | Newcastle | Port Kembla | Wollongong | Fraser Island | Harvey Bay | Arthur's Seat (BS) | Eltham Aqueduct (BS) | Fairfield Park (BS) | Flinders coastline (BS) | East Beach | East London | Qholora Mouth | Fairewood (PISI) | Dwesa | Hluleka | Hole in the wall | Port St Johns | Durban | Mtunzini | St Lucia | Tugela Mouth |
| Western Australia | Kwinana (2012) | 0.006 | 0.794 | 0.187 | 0.183 | 0.203 | 0.179 | 0.183 | 0.180 | 0.152 | 0.171 | 0.183 | 0.766 | 0.781 | 0.787 | 0.768 | 0.319 | 0.313 | 0.346 | 0.532 | 0.361 | 0.376 | 0.395 | 0.364 | 0.390 | 0.404 | 0.419 | 0.409 |
|  | Kwinana (2018) | - | 0.799 | 0.199 | 0.197 | 0.219 | 0.192 | 0.192 | 0.192 | 0.166 | 0.186 | 0.198 | 0.771 | 0.787 | 0.792 | 0.775 | 0.336 | 0.332 | 0.365 | 0.540 | 0.376 | 0.392 | 0.412 | 0.379 | 0.407 | 0.420 | 0.436 | 0.426 |
|  | Roleystone (BS) | - | - | 0.796 | 0.801 | 0.783 | 0.810 | 0.787 | 0.760 | 0.774 | 0.818 | 0.819 | 0.062 | -0.130 | -0.086 | 0.100 | 0.784 | 0.794 | 0.807 | 0.762 | 0.791 | 0.805 | 0.832 | 0.793 | 0.813 | 0.811 | 0.829 | 0.816 |
| Eastern Australia | Dunbogan | - | - | - | -0.050 | -0.001 | -0.030 | -0.039 | -0.005 | -0.008 | 0.002 | 0.040 | 0.726 | 0.746 | 0.762 | 0.727 | 0.162 | 0.175 | 0.213 | 0.434 | 0.213 | 0.199 | 0.257 | 0.216 | 0.273 | 0.291 | 0.321 | 0.292 |
|  | Iluka | - | - | - | - | 0.009 | -0.024 | -0.033 | -0.014 | -0.014 | -0.011 | 0.032 | 0.729 | 0.749 | 0.766 | 0.729 | 0.160 | 0.171 | 0.210 | 0.433 | 0.211 | 0.196 | 0.259 | 0.215 | 0.275 | 0.291 | 0.318 | 0.291 |
|  | La Perouse | - | - | - | - | - | 0.034 | -0.018 | 0.004 | 0.027 | 0.034 | 0.079 | 0.719 | 0.738 | 0.752 | 0.719 | 0.167 | 0.165 | 0.207 | 0.435 | 0.212 | 0.202 | 0.255 | 0.219 | 0.266 | 0.281 | 0.307 | 0.282 |
|  | Minnie Water | - | - | - | - | - | - | -0.008 | 0.014 | 0.009 | 0.018 | 0.046 | 0.741 | 0.763 | 0.779 | 0.744 | 0.177 | 0.187 | 0.230 | 0.449 | 0.230 | 0.218 | 0.279 | 0.231 | 0.288 | 0.308 | 0.335 | 0.310 |
|  | Newcastle | - | - | - | - | - | - | - | -0.011 | -0.007 | 0.009 | 0.037 | 0.716 | 0.734 | 0.750 | 0.715 | 0.143 | 0.153 | 0.197 | 0.421 | 0.195 | 0.180 | 0.238 | 0.199 | 0.256 | 0.273 | 0.301 | 0.273 |
|  | Port Kembla | - | - | - | - | - | - | - | - | -0.004 | 0.027 | 0.041 | 0.703 | 0.720 | 0.732 | 0.703 | 0.164 | 0.161 | 0.200 | 0.422 | 0.210 | 0.205 | 0.248 | 0.216 | 0.259 | 0.274 | 0.295 | 0.275 |
|  | Wollongong | - | - | - | - | - | - | - | - | - | 0.030 | 0.035 | 0.716 | 0.735 | 0.746 | 0.716 | 0.173 | 0.176 | 0.214 | 0.429 | 0.221 | 0.213 | 0.260 | 0.224 | 0.273 | 0.291 | 0.312 | 0.293 |
|  | Fraser Island | - | - | - | - | - | - | - | - | - | - | 0.075 | 0.748 | 0.774 | 0.789 | 0.753 | 0.185 | 0.198 | 0.240 | 0.459 | 0.239 | 0.227 | 0.293 | 0.243 | 0.301 | 0.316 | 0.351 | 0.319 |
|  | Harvey Bay | - | - | - | - | - | - | - | - | - | - | - | 0.753 | 0.780 | 0.793 | 0.757 | 0.180 | 0.180 | 0.228 | 0.470 | 0.232 | 0.227 | 0.290 | 0.237 | 0.293 | 0.310 | 0.342 | 0.314 |
|  | Arthur's Seat (BS) | - | - | - | - | - | - | - | - | - | - | - | - | -0.136 | -0.090 | -0.066 | 0.716 | 0.727 | 0.739 | 0.668 | 0.722 | 0.725 | 0.759 | 0.724 | 0.748 | 0.746 | 0.757 | 0.746 |
|  | Eltham Aqueduct (BS) | - | - | - | - | - | - | - | - | - | - | - | - | - | -0.236 | -0.031 | 0.741 | 0.753 | 0.768 | 0.725 | 0.750 | 0.755 | 0.795 | 0.751 | 0.781 | 0.778 | 0.799 | 0.782 |
|  | Fairfield Park (BS) | - | - | - | - | - | - | - | - | - | - | - | - | - | - | 0.026 | 0.754 | 0.765 | 0.780 | 0.741 | 0.761 | 0.769 | 0.807 | 0.764 | 0.791 | 0.788 | 0.809 | 0.793 |
|  | Flinders coastline (BS) | - | - | - | - | - | - | - | - | - | - | - | - | - | - | - | 0.721 | 0.733 | 0.747 | 0.694 | 0.727 | 0.731 | 0.771 | 0.730 | 0.758 | 0.756 | 0.773 | 0.757 |
| South Africa - East Beach group | East Beach | - | - | - | - | - | - | - | - | - | - | - | - | - | - | - | - | 0.135 | 0.155 | 0.427 | 0.151 | 0.139 | 0.202 | 0.161 | 0.206 | 0.226 | 0.254 | 0.227 |
|  | East London | - | - | - | - | - | - | - | - | - | - | - | - | - | - | - | - | - | 0.124 | 0.442 | 0.151 | 0.148 | 0.209 | 0.168 | 0.221 | 0.240 | 0.268 | 0.240 |
|  | Qholora Mouth | - | - | - | - | - | - | - | - | - | - | - | - | - | - | - | - | - | - | 0.461 | 0.129 | 0.143 | 0.207 | 0.166 | 0.215 | 0.237 | 0.270 | 0.237 |
|  | Fairewood (PISI) | - | - | - | - | - | - | - | - | - | - | - | - | - | - | - | - | - | - | - | 0.434 | 0.423 | 0.475 | 0.429 | 0.468 | 0.471 | 0.483 | 0.475 |
| South Africa - Port St Johns group | Dwesa | - | - | - | - | - | - | - | - | - | - | - | - | - | - | - | - | - | - | - | - | 0.091 | 0.131 | 0.127 | 0.178 | 0.203 | 0.234 | 0.200 |
|  | Hluleka | - | - | - | - | - | - | - | - | - | - | - | - | - | - | - | - | - | - | - | - | - | 0.102 | 0.046 | 0.159 | 0.185 | 0.222 | 0.181 |
|  | Hole in the wall | - | - | - | - | - | - | - | - | - | - | - | - | - | - | - | - | - | - | - | - | - | - | 0.142 | 0.216 | 0.239 | 0.276 | 0.238 |
|  | Port St Johns | - | - | - | - | - | - | - | - | - | - | - | - | - | - | - | - | - | - | - | - | - | - | - | 0.179 | 0.200 | 0.232 | 0.201 |
| South Africa – Durban group | Durban | - | - | - | - | - | - | - | - | - | - | - | - | - | - | - | - | - | - | - | - | - | - | - | - | 0.151 | 0.221 | 0.147 |
|  | Mtunzini | - | - | - | - | - | - | - | - | - | - | - | - | - | - | - | - | - | - | - | - | - | - | - | - |  | 0.157 | 0.075 |
|  | St Lucia | - | - | - | - | - | - | - | - | - | - | - | - | - | - | - | - | - | - | - | - | - | - | - | - | - | - | 0.190 |

**Table S7.** Preliminary model choice analysis results for modelling of alternative scenarios for the *Chrysanthemoides monilifera* ssp. *rotundata* (bitou bush) introduction to eastern Australia. Models including a single isolated source populations and models including admixed source populations were analysed in independent tournaments.

| **Source population** | **Admixed with** | **Bottleneck** | **RF votes (of 1000)** | **Posterior probability** |
| --- | --- | --- | --- | --- |
| *Tournament 1 - Single origin* | | | | |
| NRN | - | Y | 46 | - |
| NRS | - | Y | 131 | - |
| NRC | - | Y | 32 | - |
| GHOST1 | - | Y | 263 | - |
| NRN | - | N | 57 | - |
| NRS | - | N | 106 | - |
| NRC | - | N | 70 | - |
| GHOST1 | - | N | 295 | 0.44 |
| *Tournament 2 - Admixed origin* | | | | |
| NRN | NRC | Y | 198 |  |
| NRN | NRC | Y | 213 |  |
| NRS | NRC | Y | 247 | 0.50 |
| NRN | NRS | N | 122 |  |
| NRN | NRC | N | 79 |  |
| NRS | NRC | N | 141 |  |

**Table S8.** Model choice analysis results for alternative scenarios for the introduction of *Chrysanthemoides monilifera* ssp. *rotundata* (bitou bush) to eastern Australia.

| **Source population** | **Admixed with** | **Bottleneck** | **RF votes** | **Posterior probability** |
| --- | --- | --- | --- | --- |
| NRS | - | Y | 121 |  |
| GHOST1 | - | Y | 283 | 0.52 |
| GHOST1 | - | N | 281 |  |
| NRN | NRS | Y | 120 |  |
| NRS | NRC | Y | 92 |  |
| NRN | NRC | Y | 103 |  |

**Table S9.** Preliminary model choice analysis results for the modelling of alternative scenarios of introduction history of *Chrysanthemoides monilifera* ssp. *rotundata* (bitou bush) into Western Australia. Models including an origin in South Africa (Out of RSA) and models including an origin in eastern Australia (Out of EAU) were analysed in independent tournaments.

| **Source population** | **Admixed with** | **Bottleneck** | **RF votes (of 1000)** | **Posterior probability** |
| --- | --- | --- | --- | --- |
| *Tournament 1 - Out of RSA* | | | | |
| GHOST1 | - | Y | 143 |  |
| GHOST2 | - | Y | 253 | 0.62 |
| NRS | - | Y | 64 |  |
| NRC | - | Y | 27 |  |
| NRN | - | Y | 44 |  |
| NRN | NRS | Y | 220 |  |
| NRN | NRC | Y | 101 |  |
| NRS | NRC | Y | 148 |  |
| *Tournament 2 - Out of EAU* | | | | |
| EAU | - | Y | 60 |  |
| EAU | GHOST1 | Y | 326 |  |
| EAU | NRN | Y | 122 |  |
| EAU | NRS | Y | 327 | 0.61 |
| EAU | NRC | Y | 165 |  |

**Table S10.** Supporting analysis of the final scenario selection analysis for the modelling of alternative scenarios of introduction history of *Chrysanthemoides monilifera* ssp. *rotundata* into Western Australia, using a set of 4,000 SNPs and 10,000 simulated datasets per scenario.

| **Source population** | **Admixed with** | **Bottleneck** | **RF votes (of 1000)** | **Posterior probability** |
| --- | --- | --- | --- | --- |
| GHOST1 | - | Y | 228 |  |
| GHOST2 | - | Y | 236 |  |
| NRN | NRS | Y | 110 |  |
| NRS | NRC | Y | 71 |  |
| EAU | GHOST1 | Y | 322 | 0.54 |
| EAU | NRS | Y | 33 |  |

**Table S11.** Posterior parameter distribution, with 5% and 95% quantiles, for the final scenario modelled with DIYABC for the introduction history of *Chrysanthemoides monilifera* ssp. *rotundata* in Australia.

| **Parameter** |  | **mean** | **median** | **5%** | **95%** |
| --- | --- | --- | --- | --- | --- |
| EAU | N_e_ of eastern Australian population | 8.77E+03 | 9.11E+03 | 6.49E+03 | 9.96E+03 |
| NRN | N_e_ of native range north (Durban) population | 2.98E+01 | 2.19E+01 | 1.39E+01 | 4.63E+01 |
| NRS | N_e_ of native range south (East Beach) population | 3.05E+02 | 2.05E+02 | 7.35E+01 | 7.37E+02 |
| NRC | N_e_ of native range central (Port St. John) population | 1.38E+02 | 8.67E+01 | 3.55E+01 | 2.90E+02 |
| WAU | N_e_ of Western Australian population | 5.39E+03 | 5.66E+03 | 4.51E+02 | 9.61E+03 |
| RSA_GHOST1_ | N_e_ of South African ghost population 1 | 6.10E+02 | 3.91E+02 | 1.39E+02 | 1.75E+03 |
| RSA | N_e_ of South African ancestral population | 9.98E+03 | 9.99E+03 | 9.95E+03 | 1.00E+04 |
| t_invW_ | Divergence time of Western Australian population | 9.47E+00 | 1.00E+01 | 8.00E+00 | 1.00E+01 |
| db2 | Bottleneck time for Western Australian population | 1.57E+00 | 1.00E+00 | 1.00E+00 | 5.00E+00 |
| WAU0 | N_e_ of bottlenecked Western Australian population | 8.77E+00 | 6.53E+00 | 5.08E+00 | 1.11E+01 |
| t_adm_ | Admixture time for RSA_GHOST1_ and EAU | 1.72E+01 | 1.57E+01 | 1.02E+01 | 2.94E+01 |
| ra | Admixture rate for RSA_GHOST1_ and EAU | 2.79E-01 | 2.91E-01 | 3.80E-02 | 4.82E-01 |
| t_invE_ | Divergence time of eastern Australian population | 3.95E+01 | 3.88E+01 | 3.80E+01 | 4.50E+01 |
| db1 | Bottleneck time for eastern Australian population | 4.39E+00 | 5.00E+00 | 1.00E+00 | 5.00E+00 |
| EAU0 | N_e_ of bottlenecked eastern Australian population | 8.13E+00 | 5.00E+00 | 5.00E+00 | 1.34E+01 |
| t_anc_ | Divergence time from ancestral population | 2.13E+02 | 2.01E+02 | 1.11E+02 | 3.17E+02 |

**Table S12.** DIYABC model selection results for the final scenario for the introduction history of *Chrysanthemoides monilifera* ssp. *rotundata* (bitou bush) in Australia using mean genetic diversity, Nei’s distance, and Fst. Significant deviations from the observed values are indicated using *.

| **Summary stastic** | **Observed value** | **Proportion simulated < observed** |
| --- | --- | --- |
| HMO_1_1 | 0.2496 | 0.999 (***) |
| HMO_1_2 | 0.2346 | 1 (***) |
| HMO_1_3 | 0.2466 | 0.9765 (*) |
| HMO_1_4 | 0.2564 | 1 (***) |
| HMO_1_5 | 0.1568 | 0.7375 |
| FMO_1_1&2 | 0.2759 | 0.4625 |
| FMO_1_1&3 | 0.1612 | 0.0715 |
| FMO_1_1&4 | 0.232 | 0.271 |
| FMO_1_1&5 | 0.0961 | 0.4285 |
| FMO_1_2&3 | 0.1729 | 0.002 (**) |
| FMO_1_2&4 | 0.13 | 0 (***) |
| FMO_1_2&5 | 0.3413 | 0.962 (*) |
| FMO_1_3&4 | 0.0934 | 0.005 (**) |
| FMO_1_3&5 | 0.2493 | 0.2175 |
| FMO_1_4&5 | 0.3096 | 0.7385 |
| NMO_1_1&2 | 0.1681 | 0.0635 |
| NMO_1_1&3 | 0.0885 | 0.0645 |
| NMO_1_1&4 | 0.1287 | 0.086 |
| NMO_1_1&5 | 0.0512 | 0.356 |
| NMO_1_2&3 | 0.1111 | 0.003 (**) |
| NMO_1_2&4 | 0.0868 | 0.001 (***) |
| NMO_1_2&5 | 0.2147 | 0.138 |
| NMO_1_3&4 | 0.0599 | 0.006 (**) |
| NMO_1_3&5 | 0.1328 | 0.136 |
| NMO_1_4&5 | 0.1764 | 0.163 |

*P<0.05

**P<0.01

***P<0.001

**Table S13** Model choice analysis results for the modelling of alternative scenarios of introduction history of *Chrysanthemoides monilifera* ssp. *rotundata* (bitou bush) into Australia using South African populations inferred from hierarchical fastStructure analysis. The best scenario for the origin of Eastern Australian samples from South Africa was used for the analysis of the origin of the Western Australian population. The analysis was repeated with a subset of four Eastern Australian and four Western Australian individuals (‘Downsampled’).

|  |  |  | **All samples** | | **Downsampled** | |
| --- | --- | --- | --- | --- | --- | --- |
| **Source population** | **Admixed with** | **Bottleneck** | **RF votes (of 1000)** | **Posterior probability** | **RF votes (of 1000)** | **Posterior probability** |
| *Origin of Eastern Australian bitou bush* | | | | | | |
| GHOST1 |  | Y | 230 | 0.5538 | 321 | 0.4835 |
| EBEACH |  | Y | 134 |  | 52 |  |
| ELDN |  | Y | 98 |  | 91 |  |
| STJOHN |  | Y | 53 |  | 49 |  |
| DUR |  | Y | 75 |  | 48 |  |
| DWESA |  | Y | 111 |  | 62 |  |
| HLU |  | Y | 41 |  | 76 |  |
| HOL |  | Y | 74 |  | 82 |  |
| TMTH |  | Y | 88 |  | 58 |  |
| QMTH |  | Y | 63 |  | 94 |  |
| STLUCIA |  | Y | 33 |  | 67 |  |
| *Origin of Western Australian bitou bush* | | | | | | |
| GHOST1 |  | Y | 114 |  | 84 |  |
| GHOST2 |  | Y | 258 | 0.4923 | 259 | 0.5379 |
| EBEACH |  | Y | 50 |  | 44 |  |
| ELDN |  | Y | 34 |  | 43 |  |
| STJOHN |  | Y | 47 |  | 68 |  |
| DUR |  | Y | 55 |  | 63 |  |
| DWESA |  | Y | 41 |  | 49 |  |
| HLU |  | Y | 56 |  | 51 |  |
| HOL |  | Y | 53 |  | 105 |  |
| TMTH |  | Y | 63 |  | 37 |  |
| QMTH |  | Y | 26 |  | 50 |  |
| STLUCIA |  | Y | 28 |  | 47 |  |
| EAU |  | Y | 41 |  | 25 |  |
| EAU | GHOST1 | Y | 134 |  | 75 |  |

**Table S14** Posterior parameter distribution, with 5% and 95% quantiles, for the final scenario modelled with DIYABC for the introduction history of *Chrysanthemoides monilifera* ssp. *rotundata* (bitou bush) in Australia using South African populations inferred from hierarchical fastStructure analysis.

| **Parameter** |  | **mean** | **median** | **5%** | **95%** |
| --- | --- | --- | --- | --- | --- |
| EASTAU | N_e_ of eastern Australian population | 9.81E+03 | 9.93E+03 | 9.29E+03 | 1.00E+04 |
| DURBAN | N_e_ of Durban population | 1.55E+01 | 1.22E+01 | 1.05E+01 | 1.92E+01 |
| DWESA | N_e_ of Dwesa population | 1.23E+01 | 1.11E+01 | 1.01E+01 | 1.53E+01 |
| EBEACH | N_e_ of East Beach population | 2.05E+01 | 1.61E+01 | 1.21E+01 | 3.21E+01 |
| ELDN | N_e_ of East London population | 3.19E+02 | 2.26E+02 | 8.96E+01 | 7.32E+02 |
| HLU | N_e_ of Hluleka population | 5.92E+01 | 3.60E+01 | 1.88E+01 | 1.28E+02 |
| HOLE | N_e_ of Hole in the Wall population | 2.96E+01 | 2.02E+01 | 1.33E+01 | 5.26E+01 |
| TMTH | N_e_ of Tungela Mouth population | 2.85E+01 | 2.05E+01 | 1.38E+01 | 4.90E+01 |
| QMTH | N_e_ of Qholora Mouth population | 5.62E+01 | 3.89E+01 | 2.03E+01 | 1.14E+02 |
| STJOHN | N_e_ of Port St. John population | 8.32E+01 | 5.76E+01 | 2.81E+01 | 1.66E+02 |
| STLUCIA | N_e_ of St. Lucia population | 6.02E+01 | 4.50E+01 | 2.44E+01 | 1.10E+02 |
| WESTAU | N_e_ of Western Australian population | 5.39E+03 | 5.54E+03 | 5.88E+02 | 9.63E+03 |
| RSA_GHOST1_ | N_e_ of South African ghost population 1 | 1.26E+01 | 1.11E+01 | 1.02E+01 | 1.41E+01 |
| RSA | N_e_ of South African ancestral population | 9.58E+03 | 9.73E+03 | 8.77E+03 | 9.97E+03 |
| RSA_GHOST2_ | N_e_ of South African ghost population 1 | 9.20E+01 | 8.34E+01 | 5.10E+01 | 1.39E+02 |
| t_invW_ | Divergence time of Western Australian population | 9.75E+00 | 1.00E+01 | 8.00E+00 | 1.00E+01 |
| db2 | Bottleneck time for Western Australian population | 4.12E+00 | 5.00E+00 | 1.00E+00 | 5.00E+00 |
| WESTAU0 | N_e_ of bottlenecked Western Australian population | 1.06E+02 | 2.85E+01 | 1.05E+01 | 3.46E+02 |
| t_invE_ | Divergence time of eastern Australian population | 4.49E+01 | 4.50E+01 | 4.47E+01 | 4.50E+01 |
| db1 | Bottleneck time for eastern Australian population | 1.96E+00 | 1.00E+00 | 1.00E+00 | 5.00E+00 |
| EASTAU0 | N_e_ of bottlenecked eastern Australian population | 9.34E+03 | 9.78E+03 | 7.06E+03 | 9.99E+03 |
| t_anc_ | Divergence time from ancestral population | 5.12E+01 | 5.00E+01 | 3.76E+01 | 6.67E+01 |

**Table S15** Posterior parameter distribution, with 5% and 95% quantiles, for the final scenario modelled with DIYABC for the introduction history of *Chrysanthemoides monilifera* ssp. *rotundata* (bitou bush) in Australia using downsampled Australian populations and South African populations inferred from hierarchical fastStructure analysis.

| **Parameter** |  | **mean** | **median** | **5%** | **95%** |
| --- | --- | --- | --- | --- | --- |
| EASTAU | N_e_ of eastern Australian population | 2.73E+02 | 6.98E+01 | 1.25E+01 | 1.13E+03 |
| DURBAN | N_e_ of Durban population | 2.98E+02 | 2.26E+02 | 9.71E+01 | 6.14E+02 |
| DWESA | N_e_ of Dwesa population | 5.71E+01 | 4.11E+01 | 2.16E+01 | 1.04E+02 |
| EBEACH | N_e_ of East Beach population | 1.69E+02 | 1.19E+02 | 5.09E+01 | 3.47E+02 |
| ELDN | N_e_ of East London population | 4.21E+02 | 3.20E+02 | 1.32E+02 | 9.52E+02 |
| HLU | N_e_ of Hluleka population | 2.49E+03 | 2.15E+03 | 8.84E+02 | 5.41E+03 |
| HOLE | N_e_ of Hole in the Wall population | 5.30E+01 | 3.35E+01 | 1.82E+01 | 1.02E+02 |
| TMTH | N_e_ of Tungela Mouth population | 1.99E+02 | 1.35E+02 | 5.69E+01 | 3.87E+02 |
| QMTH | N_e_ of Qholora Mouth population | 4.47E+01 | 3.12E+01 | 1.74E+01 | 7.71E+01 |
| STJOHN | N_e_ of Port St. John population | 1.70E+02 | 1.25E+02 | 5.67E+01 | 3.07E+02 |
| STLUCIA | N_e_ of St. Lucia population | 1.71E+02 | 1.39E+02 | 6.70E+01 | 3.01E+02 |
| WESTAU | N_e_ of Western Australian population | 9.97E+03 | 1.00E+04 | 9.90E+03 | 1.00E+04 |
| RSA_GHOST1_ | N_e_ of South African ghost population 1 | 3.84E+01 | 2.14E+01 | 1.38E+01 | 5.95E+01 |
| RSA | N_e_ of South African ancestral population | 9.99E+03 | 1.00E+04 | 9.97E+03 | 1.00E+04 |
| RSA_GHOST2_ | N_e_ of South African ghost population 1 | 5.69E+01 | 4.12E+01 | 2.25E+01 | 9.78E+01 |
| t_invW_ | Divergence time of Western Australian population | 8.47E+00 | 8.00E+00 | 8.00E+00 | 1.00E+01 |
| db2 | Bottleneck time for Western Australian population | 1.32E+00 | 1.00E+00 | 1.00E+00 | 4.55E+00 |
| WESTAU0 | N_e_ of bottlenecked Western Australian population | 5.81E+03 | 6.17E+03 | 8.28E+02 | 9.69E+03 |
| t_invE_ | Divergence time of eastern Australian population | 4.23E+01 | 4.29E+01 | 3.80E+01 | 4.50E+01 |
| db1 | Bottleneck time for eastern Australian population | 2.09E+00 | 1.09E+00 | 1.00E+00 | 5.00E+00 |
| EASTAU0 | N_e_ of bottlenecked eastern Australian population | 4.00E+03 | 3.58E+03 | 3.00E+02 | 9.04E+03 |
| t_anc_ | Divergence time from ancestral population | 1.06E+02 | 1.06E+02 | 7.75E+01 | 1.37E+02 |

**Table S16.** Inferred ploidy of *Chrysanthemoides monilifera* (bitou bush and related subspecies) samples used in this study, based on nQuire analysis of SNPs.

| **Sample ID** | **Ploidy** | **Delta likelihood** | **Total bases** | **Inference status** |
| --- | --- | --- | --- | --- |
| EAU_NSW_DUN_BB_01 | diploid | 753.397775 | 57996 | accepted |
| EAU_NSW_DUN_BB_02 | diploid | 1077.99336 | 77571 | accepted |
| EAU_NSW_DUN_BB_03 | diploid | 210.77917 | 72954 | accepted |
| EAU_NSW_ILUKA_BB_01 | diploid | 908.211959 | 48332 | accepted |
| EAU_NSW_ILUKA_BB_02 | diploid | 20528.62257 | 57680 | accepted |
| EAU_NSW_ILUKA_BB_03 | tetraploid | -362.608644 | 30019 | accepted |
| EAU_NSW_KEMBLA_BB_01 | diploid | 168.573872 | 63477 | accepted |
| EAU_NSW_KEMBLA_BB_02 | diploid | -1124.977278 | 65550 | accepted |
| EAU_NSW_KEMBLA_BB_03 | diploid | 733.910321 | 71993 | accepted |
| EAU_NSW_KEMBLA_BB_04 | diploid | 868.038598 | 77156 | accepted |
| EAU_NSW_KEMBLA_BB_05 | diploid | 1202.894297 | 66103 | accepted |
| EAU_NSW_KEMBLA_BB_06 | diploid | 829.818828 | 63214 | accepted |
| EAU_NSW_LAPER_BB_01 | diploid | 1602.908565 | 72218 | accepted |
| EAU_NSW_LAPER_BB_02 | diploid | 36149.12786 | 65562 | accepted |
| EAU_NSW_LAPER_BB_03 | diploid | 994.110871 | 52169 | accepted |
| EAU_NSW_LAPER_BB_04 | diploid | 1331.665413 | 63507 | accepted |
| EAU_NSW_MINNIE_BB_01 | diploid | 1492.080545 | 73321 | accepted |
| EAU_NSW_MINNIE_BB_02 | diploid | 945.10396 | 59398 | accepted |
| EAU_NSW_MINNIE_BB_03 | diploid | 295.559198 | 62230 | accepted |
| EAU_NSW_NEW_BB_01 | diploid | 2289.509579 | 78561 | accepted |
| EAU_NSW_NEW_BB_02 | diploid | 734.487265 | 78855 | accepted |
| EAU_NSW_NEW_BB_03 | diploid | 577.667997 | 50488 | accepted |
| EAU_NSW_WOL_BB_01 | triploid | 387.611841 | 5483 | ambiguous |
| EAU_NSW_WOL_BB_02 | diploid | 44302.5606 | 78307 | accepted |
| EAU_NSW_WOL_BB_03 | diploid | 976.495872 | 63697 | accepted |
| EAU_NSW_WOL_BB_04 | diploid | 3256.262589 | 52484 | accepted |
| EAU_NSW_WOL_BB_05 | triploid | 491.586692 | 9922 | ambiguous |
| EAU_NSW_WOL_BB_06 | diploid | 689.01023 | 62362 | accepted |
| EAU_NSW_WOL_BB_07 | diploid | 467.252444 | 51679 | accepted |
| EAU_QLD_FRASER_BB_01 | diploid | 280.326719 | 59624 | accepted |
| EAU_QLD_FRASER_BB_02 | diploid | 7.312815 | 60493 | accepted |
| EAU_QLD_FRASER_BB_03 | diploid | 796.538643 | 49086 | accepted |
| EAU_QLD_HARVEY_BB_01 | diploid | 2128.485709 | 82402 | accepted |
| EAU_QLD_HARVEY_BB_02 | diploid | 26051.13962 | 47376 | accepted |
| EAU_QLD_HARVEY_BB_03 | diploid | 7164.550716 | 32948 | accepted |
| EAU_VIC_ARTH_BS_01 | tetraploid | 1332.006249 | 15930 | ambiguous |
| EAU_VIC_ARTH_BS_02 | diploid | 21423.34803 | 12180 | ambiguous |
| EAU_VIC_ARTH_BS_03 | diploid | 591.745911 | 16335 | ambiguous |
| EAU_VIC_ARTH_BS_04 | tetraploid | 1375.62166 | 27033 | ambiguous |
| EAU_VIC_ARTH_BS_05 | diploid | 2021.152756 | 27035 | ambiguous |
| EAU_VIC_ARTH_BS_06 | triploid | 241.869017 | 5559 | ambiguous |
| EAU_VIC_ELTH_BS_01 | diploid | 587.418816 | 15397 | ambiguous |
| EAU_VIC_ELTH_BS_02 | diploid | 928.893781 | 19257 | ambiguous |
| EAU_VIC_FAIR_BS_01 | diploid | 828.75246 | 14548 | ambiguous |
| EAU_VIC_FAIR_BS_02 | diploid | 1006.692373 | 13655 | ambiguous |
| EAU_VIC_FAIR_BS_03 | diploid | 380.328855 | 16301 | ambiguous |
| EAU_VIC_FLIN_BS_01 | diploid | 1246.552156 | 16340 | ambiguous |
| EAU_VIC_FLIN_BS_02 | diploid | 961.079369 | 17868 | ambiguous |
| EAU_VIC_FLIN_BS_03 | diploid | 967.122969 | 21160 | ambiguous |
| RSA_DUR_BB_01 | diploid | 33686.14173 | 54196 | accepted |
| RSA_DUR_BB_02 | diploid | 1553.199347 | 66257 | accepted |
| RSA_DUR_BB_03 | diploid | 1277.332835 | 70940 | accepted |
| RSA_DUR_BB_04 | diploid | -2380.718133 | 79192 | accepted |
| RSA_DWESA_BB_01 | diploid | 339.604732 | 69443 | accepted |
| RSA_DWESA_BB_02 | diploid | 787.110232 | 61878 | accepted |
| RSA_DWESA_BB_03 | diploid | -1280.514478 | 54815 | accepted |
| RSA_DWESA_BB_04 | diploid | 1089.355524 | 38584 | accepted |
| RSA_EBEACH_BB_01 | diploid | 791.18595 | 61529 | accepted |
| RSA_EBEACH_BB_02 | diploid | 1506.430623 | 119068 | accepted |
| RSA_EBEACH_BB_03 | diploid | 3165.480377 | 150796 | accepted |
| RSA_EBEACH_BB_04 | diploid | 298.372393 | 76708 | accepted |
| RSA_ELDN_BB_01 | diploid | 2150.592622 | 50888 | accepted |
| RSA_ELDN_BB_02 | diploid | 1824.268543 | 78230 | accepted |
| RSA_ELDN_BB_03 | diploid | 868.840737 | 66007 | accepted |
| RSA_ELDN_BB_04 | diploid | 1553.703497 | 67957 | accepted |
| RSA_FAIRE_PISI_01 | diploid | 1125.905491 | 112317 | accepted |
| RSA_FAIRE_PISI_02 | diploid | 58368.38162 | 142031 | accepted |
| RSA_FAIRE_PISI_03 | diploid | 870.022172 | 73447 | accepted |
| RSA_FAIRE_PISI_04 | diploid | 950.395661 | 63777 | accepted |
| RSA_HLU_BB_01 | diploid | 958.523076 | 78066 | accepted |
| RSA_HLU_BB_02 | diploid | 1368.130056 | 45149 | accepted |
| RSA_HLU_BB_03 | diploid | 842.653058 | 34418 | accepted |
| RSA_HOLE_BB_01 | tetraploid | -72.803501 | 1606 | ambiguous |
| RSA_HOLE_BB_02 | diploid | 945.446091 | 98996 | accepted |
| RSA_HOLE_BB_03 | diploid | 1348.199377 | 56791 | accepted |
| RSA_HOLE_BB_04 | diploid | 699.412684 | 95878 | accepted |
| RSA_MZN_BB_01 | diploid | 976.828035 | 70070 | accepted |
| RSA_MZN_BB_02 | diploid | 107.690597 | 70823 | accepted |
| RSA_MZN_BB_03 | diploid | 141.049694 | 65019 | accepted |
| RSA_MZN_BB_04 | diploid | 38049.35131 | 66023 | accepted |
| RSA_QMTH_BB_01 | diploid | 832.68001 | 77077 | accepted |
| RSA_QMTH_BB_02 | diploid | 425.144564 | 42929 | accepted |
| RSA_QMTH_BB_03 | diploid | 32486.56751 | 38763 | accepted |
| RSA_QMTH_BB_04 | diploid | 1034.565064 | 56472 | accepted |
| RSA_STJOHN_BB_01 | triploid | 3933.360385 | 49350 | accepted |
| RSA_STJOHN_BB_02 | diploid | 1383.777114 | 65058 | accepted |
| RSA_STJOHN_BB_03 | diploid | 987.857577 | 63131 | accepted |
| RSA_STJOHN_BB_04 | diploid | 1192.900993 | 51827 | accepted |
| RSA_STLUC_BB_01 | diploid | -1424.170611 | 60914 | accepted |
| RSA_STLUC_BB_02 | diploid | 689.959105 | 40393 | accepted |
| RSA_STLUC_BB_03 | diploid | 48871.21978 | 70443 | accepted |
| RSA_STLUC_BB_04 | diploid | 905.579096 | 42519 | accepted |
| RSA_TMTH_BB_01 | diploid | 951.227202 | 48362 | accepted |
| RSA_TMTH_BB_02 | diploid | 1018.178738 | 60558 | accepted |
| RSA_TMTH_BB_03 | diploid | 766.52014 | 44386 | accepted |
| RSA_TMTH_BB_04 | triploid | 872.234856 | 418210 | accepted |
| WAU_KWIN12_BB_107 | diploid | -346.445961 | 65980 | accepted |
| WAU_KWIN12_BB_11 | diploid | 5146.824039 | 52408 | accepted |
| WAU_KWIN12_BB_12 | diploid | 2239.363525 | 65579 | accepted |
| WAU_KWIN12_BB_150 | diploid | 12890.02064 | 63340 | accepted |
| WAU_KWIN12_BB_200 | diploid | 802.789113 | 60008 | accepted |
| WAU_KWIN12_BB_226 | diploid | 1383.614378 | 45490 | accepted |
| WAU_KWIN12_BB_249 | diploid | 410.334889 | 15091 | ambiguous |
| WAU_KWIN12_BB_277 | diploid | 1068.307149 | 66089 | accepted |
| WAU_KWIN12_BB_348 | diploid | 1056.442672 | 54466 | accepted |
| WAU_KWIN12_BB_410 | diploid | 1286.785015 | 58825 | accepted |
| WAU_KWIN12_BB_414 | diploid | 35291.94135 | 60862 | accepted |
| WAU_KWIN12_BB_415 | diploid | 453.373263 | 47208 | accepted |
| WAU_KWIN12_BB_416 | diploid | 2301.754543 | 68204 | accepted |
| WAU_KWIN12_BB_510 | diploid | 117648.9681 | 65118 | accepted |
| WAU_KWIN12_BB_511 | triploid | 397.253724 | 6008 | ambiguous |
| WAU_KWIN12_BB_512 | diploid | 532.879691 | 53315 | accepted |
| WAU_KWIN12_BB_565 | diploid | 826.351329 | 52766 | accepted |
| WAU_KWIN12_BB_620 | tetraploid | 914.318845 | 23643 | ambiguous |
| WAU_KWIN12_BB_621 | diploid | 42026.74502 | 59383 | accepted |
| WAU_KWIN12_BB_625 | diploid | -496.396456 | 53505 | accepted |
| WAU_KWIN12_BB_626 | diploid | 4.457859 | 65860 | accepted |
| WAU_KWIN18_BB_01 | diploid | 1393.476108 | 66412 | accepted |
| WAU_KWIN18_BB_02 | diploid | 240.999378 | 48728 | accepted |
| WAU_KWIN18_BB_03 | diploid | 35097.49884 | 60967 | accepted |
| WAU_KWIN18_BB_04 | diploid | 2170.434383 | 66055 | accepted |
| WAU_KWIN18_BB_05 | diploid | 928.835965 | 57867 | accepted |
| WAU_KWIN18_BB_06 | diploid | 596.513609 | 53023 | accepted |
| WAU_KWIN18_BB_07 | diploid | 545.084056 | 44335 | accepted |
| WAU_KWIN18_BB_08 | diploid | 575.070678 | 47709 | accepted |
| WAU_KWIN18_BB_09 | diploid | 684.212631 | 51905 | accepted |
| WAU_KWIN18_BB_10 | diploid | 505.130607 | 50987 | accepted |
| WAU_KWIN18_BB_11 | diploid | 644.15904 | 43709 | accepted |
| WAU_KWIN18_BB_12 | diploid | 759.774263 | 35172 | accepted |
| WAU_KWIN18_BB_13 | diploid | 4.249501 | 73892 | accepted |
| WAU_KWIN18_BB_14 | diploid | 1041.210213 | 69573 | accepted |
| WAU_KWIN18_BB_15 | diploid | 1218.789745 | 47794 | accepted |
| WAU_KWIN18_BB_16 | diploid | 37162.83697 | 60397 | accepted |
| WAU_KWIN18_BB_17 | diploid | 741.464486 | 46203 | accepted |
| WAU_KWIN18_BB_18 | diploid | 205.745524 | 16940 | ambiguous |
| WAU_KWIN18_BB_19 | diploid | 55314.1277 | 49513 | accepted |
| WAU_KWIN18_BB_20 | diploid | 786.056451 | 70881 | accepted |
| WAU_ROLEY_BS_01 | diploid | 1069.513946 | 17493 | ambiguous |
| WAU_ROLEY_BS_02 | diploid | 1367.376466 | 27710 | ambiguous |
| WAU_ROLEY_BS_03 | diploid | 2195.08142 | 19668 | ambiguous |
| WAU_ROLEY_BS_04 | diploid | -368.564716 | 19105 | ambiguous |
| WAU_ROLEY_BS_05 | diploid | 1103.228671 | 23411 | ambiguous |
| WAU_ROLEY_BS_06 | diploid | 1687.505919 | 19064 | ambiguous |

**Table S17.** Individual measurements of nuclear DNA content, genome size, coefficient of variance (CV) and ploidy for *Chrysanthemoides monilifera* ssp. *rotundata* (bitou bush) using *Solanum lycopersicum* (tomato) cultivar ‘Stupické polní rané’ as a reference standard.

| **Species** | **Nuclear DNA content 2C (pg)** | **Genome size 1C  (Mbp)** | **CV  (%)** | **DNA ploidy** |
| --- | --- | --- | --- | --- |
| Bitou bush | 3.12 | 1524 | 3.52 | Diploid |
| Bitou bush | 3.09 | 1509 | 3.59 | Diploid |
| Bitou bush | 3.09 | 1509 | 3.93 | Diploid |
| Bitou bush | 3.12 | 1527 | 4.42 | Diploid |
| Bitou bush | 3.12 | 1526 | 4.68 | Diploid |
| Mean | 3.11 | 1519 | 4.03 | - |
| SE (±) | 0.01 | 4 | 0.23 | - |

**Table S18.** Literature records of chromosome counts for *Chrysanthemoides monilifera* (bitou bush and relatives) including intra-specific taxa**.**

World chromosome counts for *Chrysanthemoides* are summarised at <http://ccdb.tau.ac.il/Angiosperms/Compositae/Chrysanthemoides/> (Rice *et al.* 2015) and are listed in the Table below. This was verified in the Index to chromosome numbers in the Asteraceae (http://www.lib.kobe-u.ac.jp/infolib/meta_pub/G0000003asteraceae_e).

| **Putative *Chrysanthemoides* taxa**  **(number of plants sampled)** | **n** | **2n** | **Source** | **Literature source** | **Comment and/or quote from source** |
| --- | --- | --- | --- | --- | --- |
| Boneseed  (possibly one plant, method not clear, could be root tips from seedlings) |  | 20 | The author’s garden, Sweden | Norlindh 1963 | Norlindh states “The south-western Cape race, *C. monilifera s. str.* Characterized, *inter alia*, by globose or subglobose putamina, has been cultivated by me for more than thirty years. During this time one specimen grew to a small tree. The chromosome number of this taxon was counted as 2n = 20”. Norlindh made the original descriptions of the subspecies. The region of origin and shape of the putamina also confirms the identification as s*. str*., i.e. *C. m. monilifera*. |
| *C. monilifera* ssp. ?  (two seedlings) |  | 18, 36 | Botanical gardens in Pretoria or Cape Town | Riley & Hoff 1961 | “The somatic chromosome number is 18 (Fig. 30) in one seedling and 36 (Fig. 31) in another.” The identification given is *C. m. rotundata*. The Methods state that seeds [for a range of plants studied] were obtained from Pretoria and/or Kirstenbosch, both of which are outside the native range of *C. m rotundata*. The identification should be regarded as tentative as there does not appear to be a voucher specimen to check. |
| *C. monilifera* ssp. ?  (pollen) | 10 |  | Botanical garden California ex South Africa | Strother 1983 | Strother (1983) states “n = 10, late first anaphase. Grown in Berkeley; U.C.B.G. 71.479; origin: S. Africa, 14 mi W of Kareedouw in Langkloof”. The subspecies is uncertain. The location in South Africa is too far east to be *C. m. monilifera* and the ray floret counts (10-11) (illustration in Strother 2017) too high also to be that subspecies (usually nearer to 7) |
| Boneseed (one plant, pollen) | 10 |  | Field collected in Victoria | Turner 1970 | The sample was collected from 7 mile. S of Mildura, Victoria. The voucher specimen (T5197) is identified as *C. m. monilifera* in <https://www.ala.org.au/>. Other specimens near Mildura are all the same subspecies. |

**
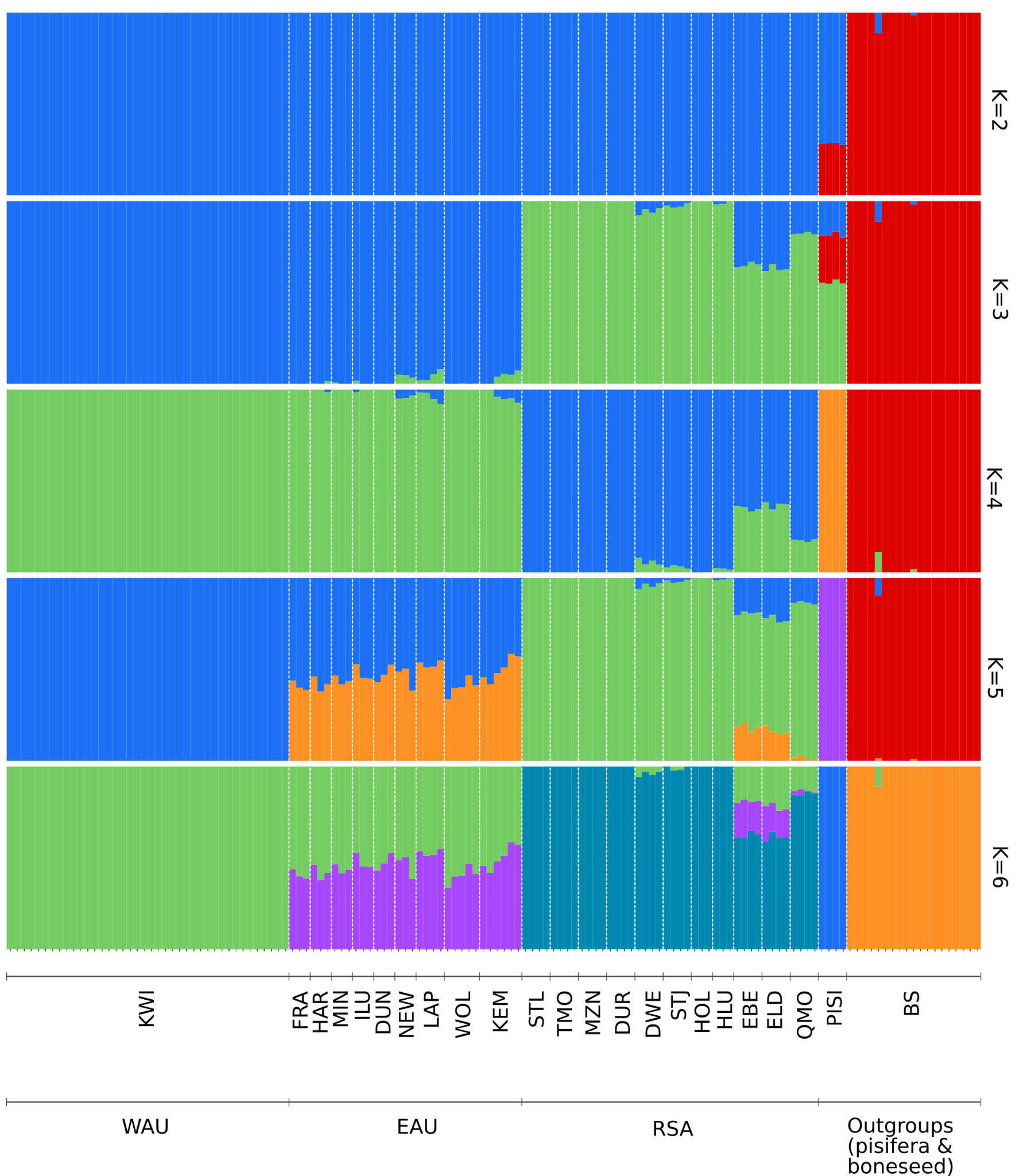
**

**Figure S1:** faststructure analysis of three subspecies of *Chrysanthemoides monilifera* showing the assignment probabilities of individuals to genetic clusters from *K* = 2 to *K* = 6. WAU = Western Australia; EAU = eastern Australia; RSA = South Africa; PISI = subspecies *pisifera*; BS = subspecies *monilifera* (boneseed). Where unspecified samples are of subspecies *rotundata* (bitou bush).

**
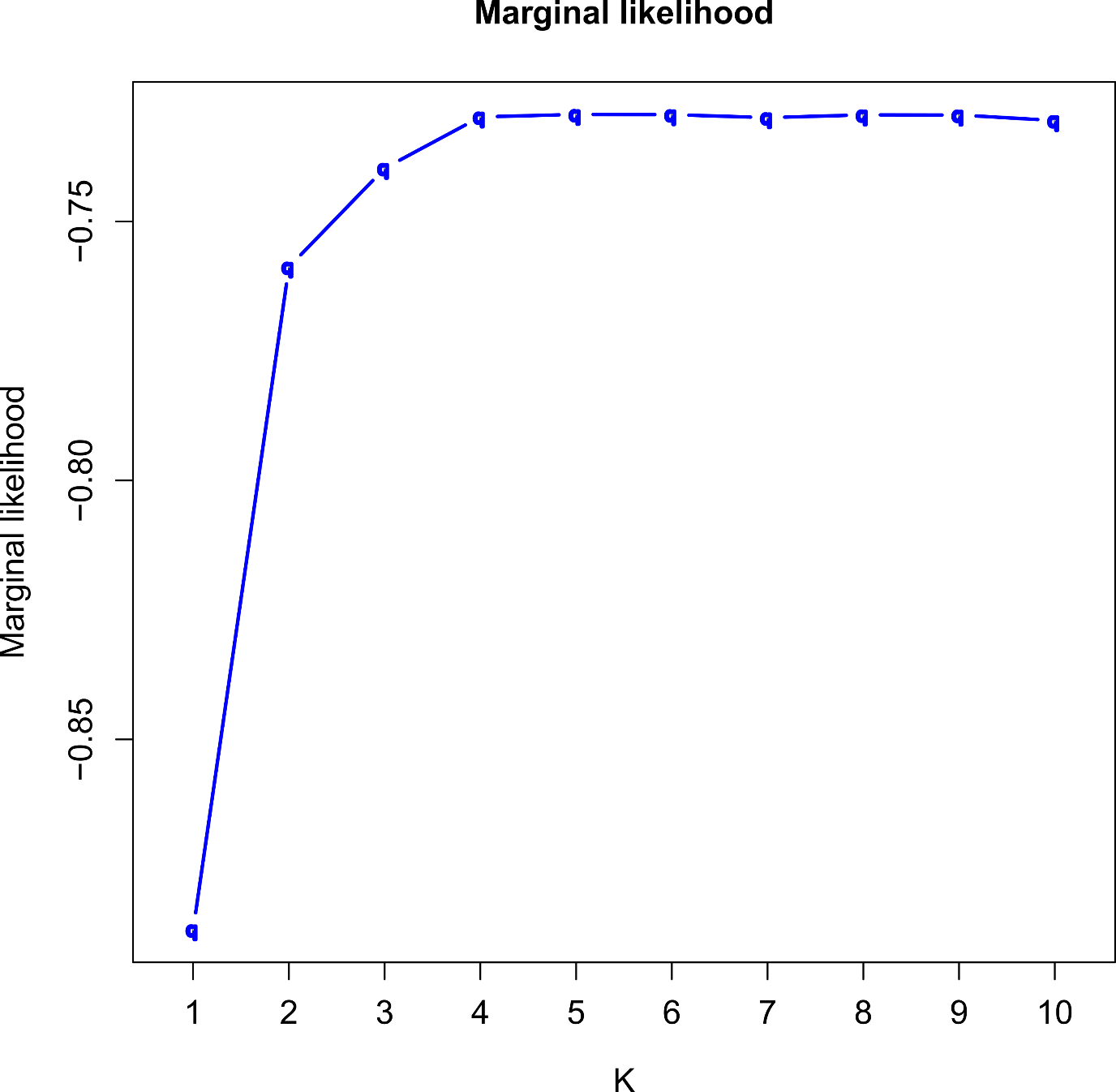
 Figure S2** Marginal likelihood of the faststructure analysis of the core dataset of *Chrysanthemoides monilifera* ssp. *rotundata* (bitou bush), excluding *C. monilifera* ssp. *monilifera* (boneseed) and *C. monilifera* ssp. *pisifera* (pisifera) individuals, across 1-10 genetic clusters (*K*). Based on faststructure *chooseK* and the Puchmaille estimator, the optimal value for *K* = 5.


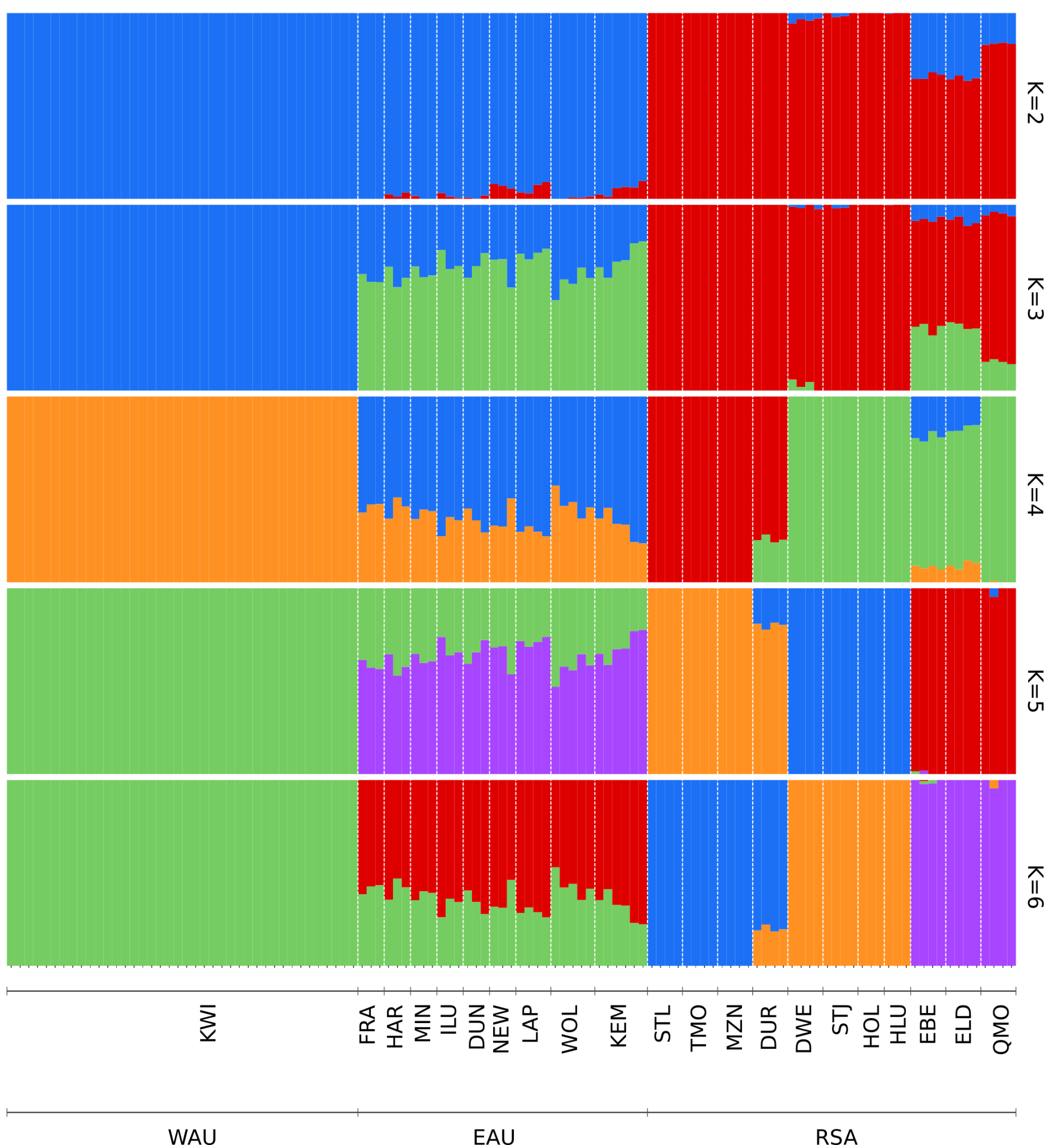


**Figure S3**  faststructure analysis showing the assignment probabilities of *Chrysanthemoides monilifera* ssp. *rotundata* (bitou bush) individuals to genetic clusters across different *K*-values, with *K* = 5 being optimal. WAU = Western Australia; EAU = eastern Australia; RSA = South Africa.

**
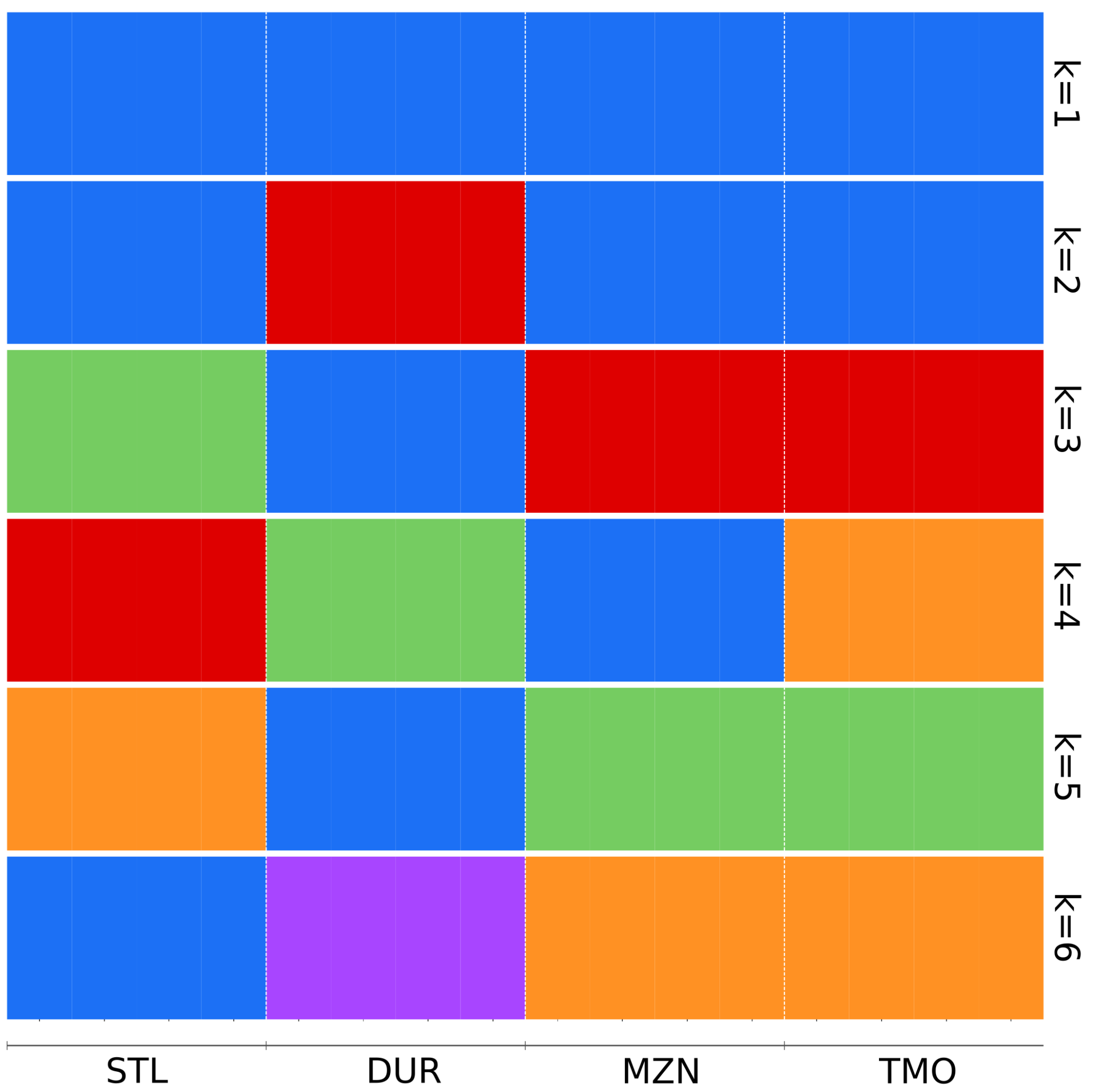
**

**Figure S4** faststructure analysis showing the assignment probabilities of *Chrysanthemoides monilifera* ssp. *rotundata* (bitou bush) individuals from the South African St. Lucia cluster across different *K*-values, with *K* = 3 being optimal. Note that ancestry contributions for some subpopulations may be so low that there are fewer than K colours.

**
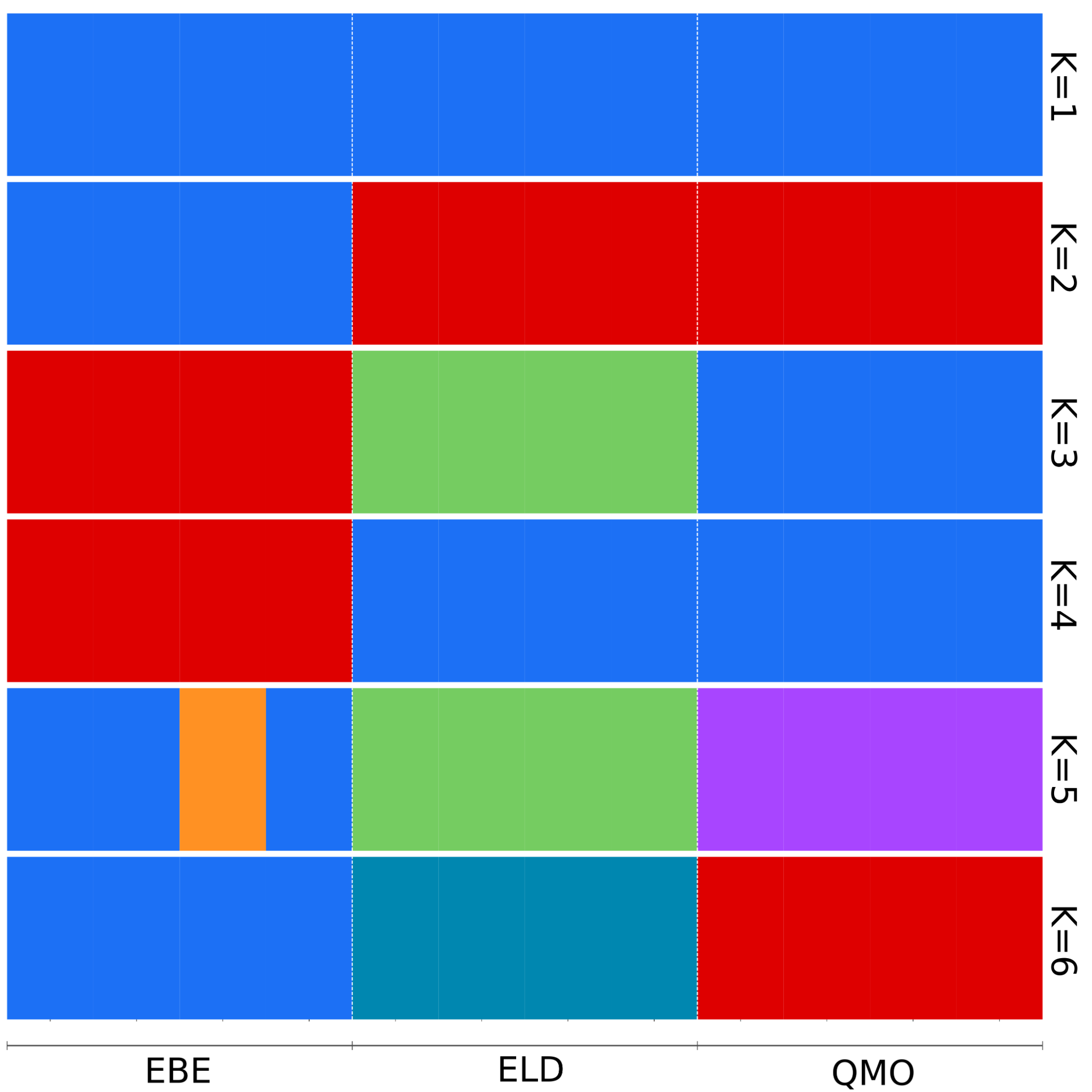
**

**Figure S5** faststructure analysis showing the assignment probabilities of *Chrysanthemoides monilifera* ssp. *rotundata* (bitou bush) individuals from the South African East Beach cluster across different *K*-values, with *K* = 3 being optimal. Note that ancestry contributions for some subpopulations may be so low that there are fewer than K colours.

**
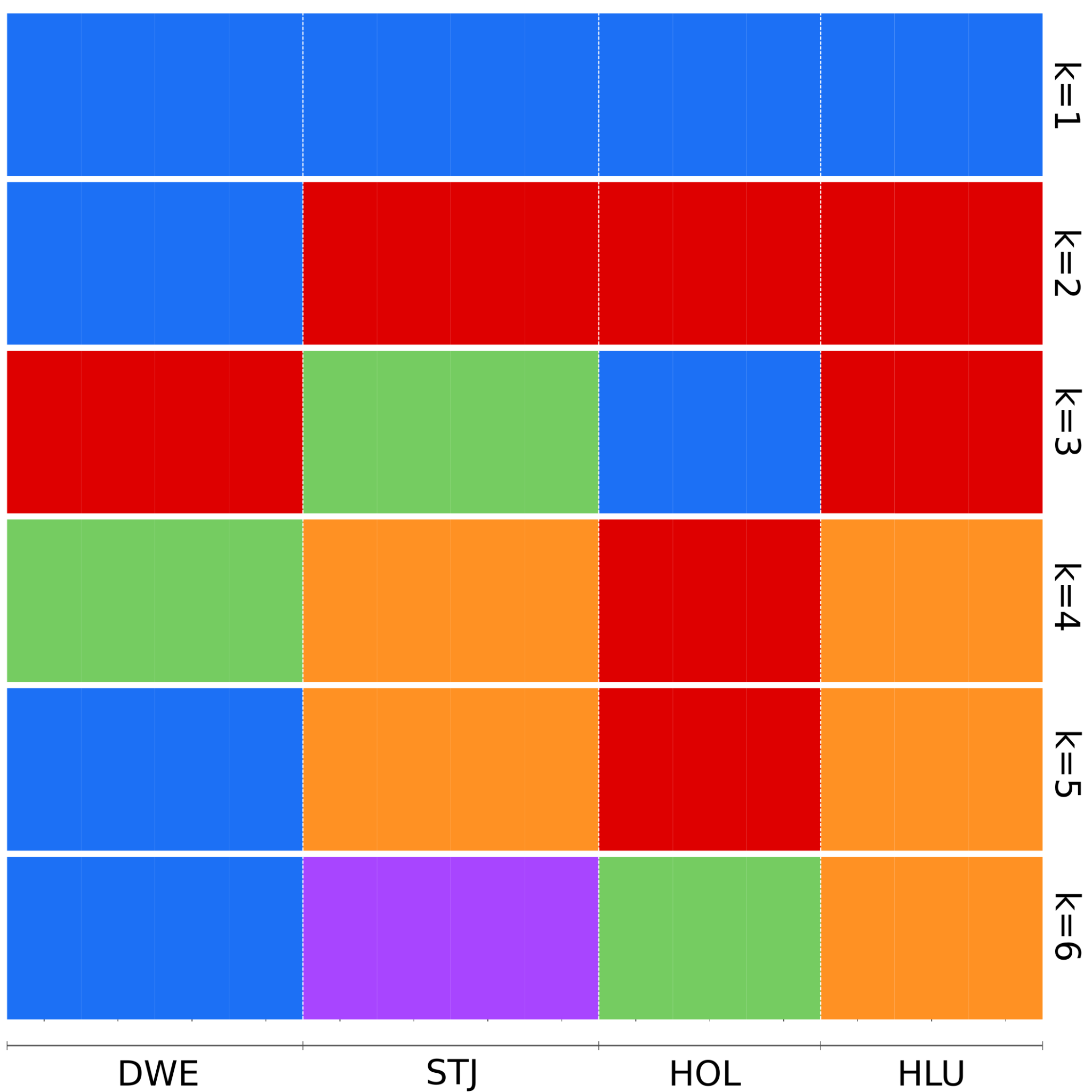
**

**Figure S6** faststructure analysis showing the assignment probabilities of *Chrysanthemoides monilifera* ssp. *rotundata* (bitou bush) individuals from the South African St. John cluster across different *K*-values, with *K* = 4 being optimal. Note that ancestry contributions for some subpopulations may be so low that there are fewer than K colours.

**
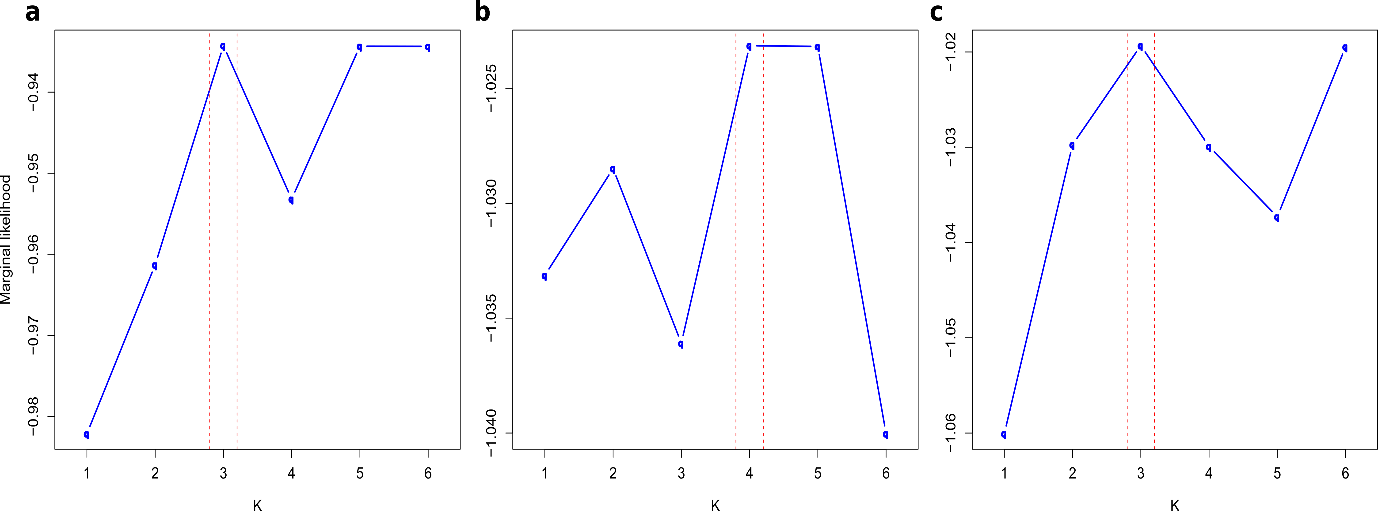
**

**Figure S7** Marginal likelihood of the faststructure analysis of the three South African clusters (**a:** Native Range North, **b:** Native Range Central, **c:** Native Range South) of *Chrysanthemoides monilifera* ssp. *rotundata* (bitou bush) across 1-6 genetic clusters (*K*).

**
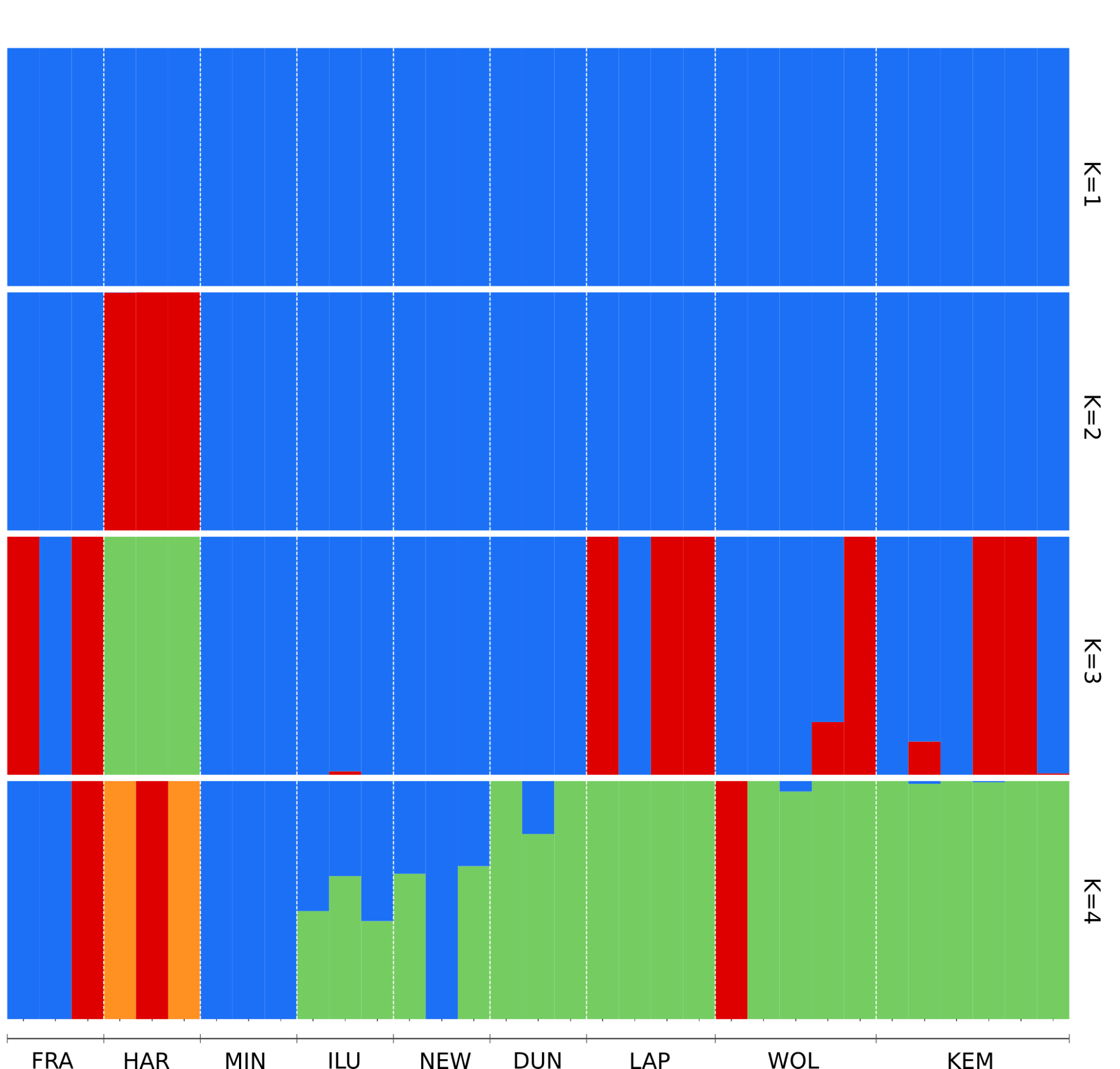
Figure S8** faststructure analysis showing the assignment probabilities of *Chrysanthemoides monilifera* ssp. *rotundata* (bitou bush) individuals from eastern Australia to genetic clusters across different *K*-values, with *K* = 1 being optimal.


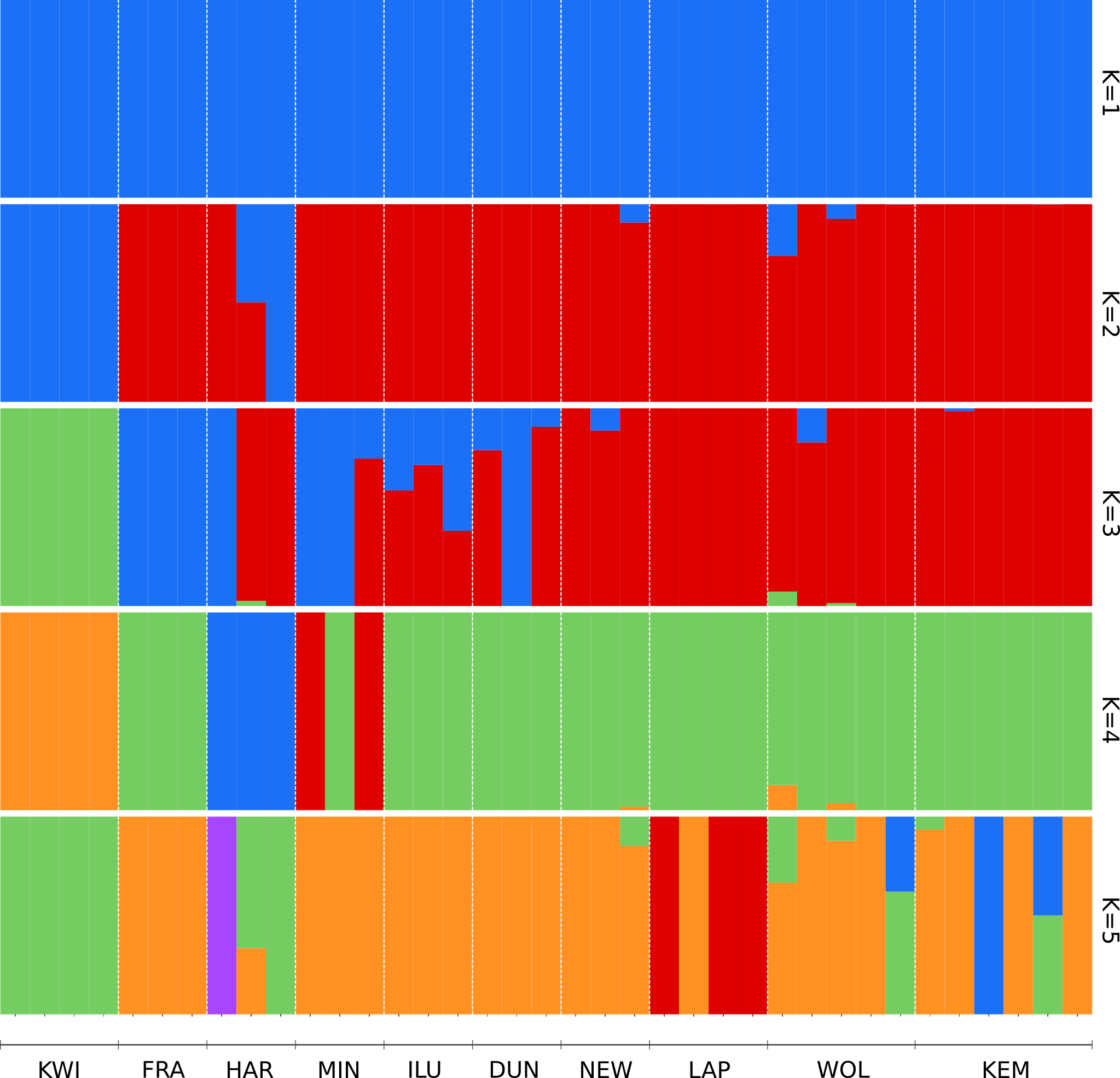


**Figure S9** faststructure analysis showing the assignment probabilities of *Chrysanthemoides monilifera* ssp. *rotundata* (bitou bush) individuals from Australia to genetic clusters across different *K*-values, with *K* = 2 being optimal.


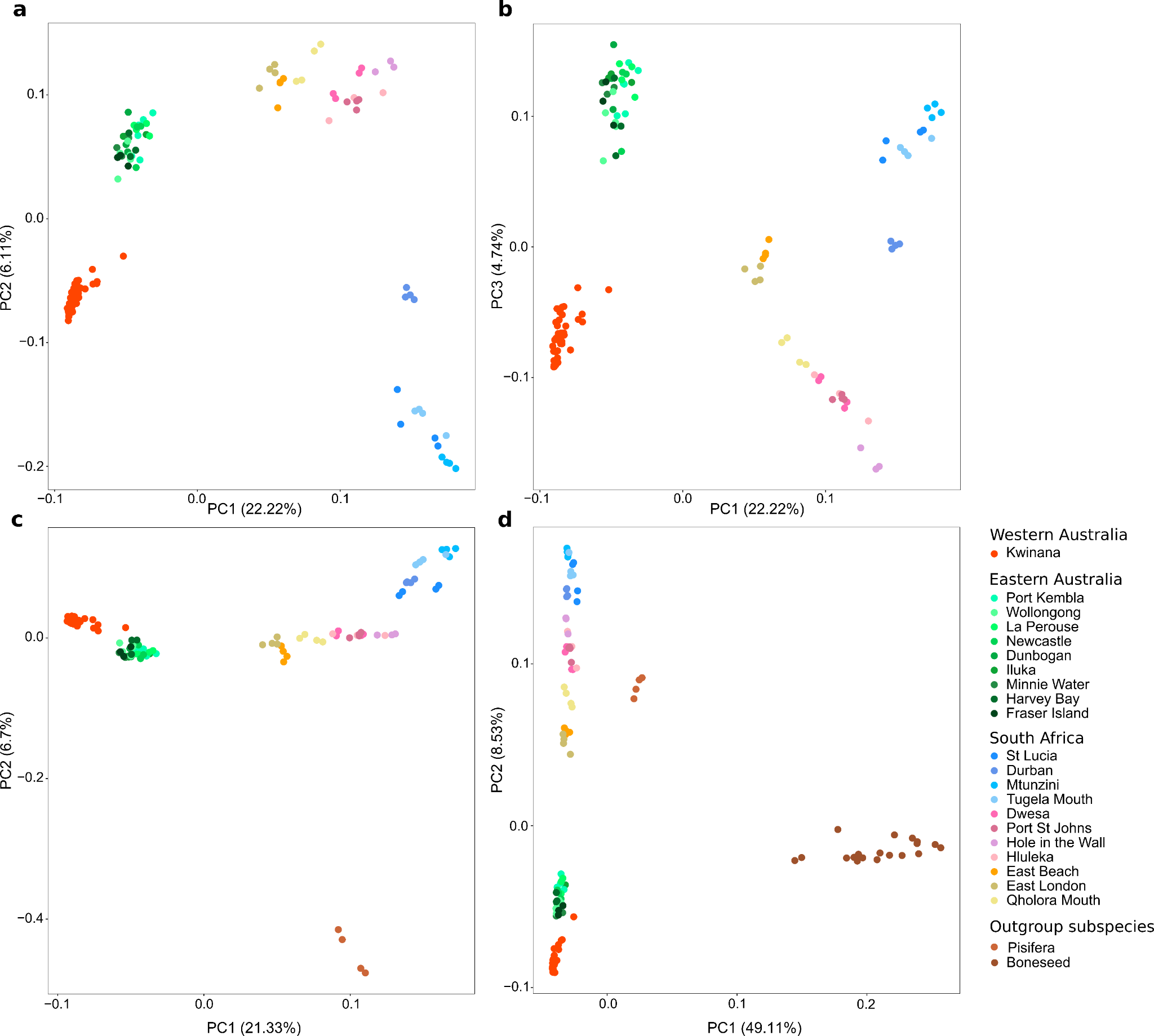


**Figure S10.** SNP-based principal component analysis (PCA) of bitou bush. The percentage of variance explained is shown on each axis. **a**: First and second principal components for the core bitou bush sample set, **b**: First and third principal components for the core bitou bush sample set, **c**: First and second principal components for the bitou-pisifera sample set, **d**: First and second principal components for the bitou-pisifera-boneseed sample set.

**
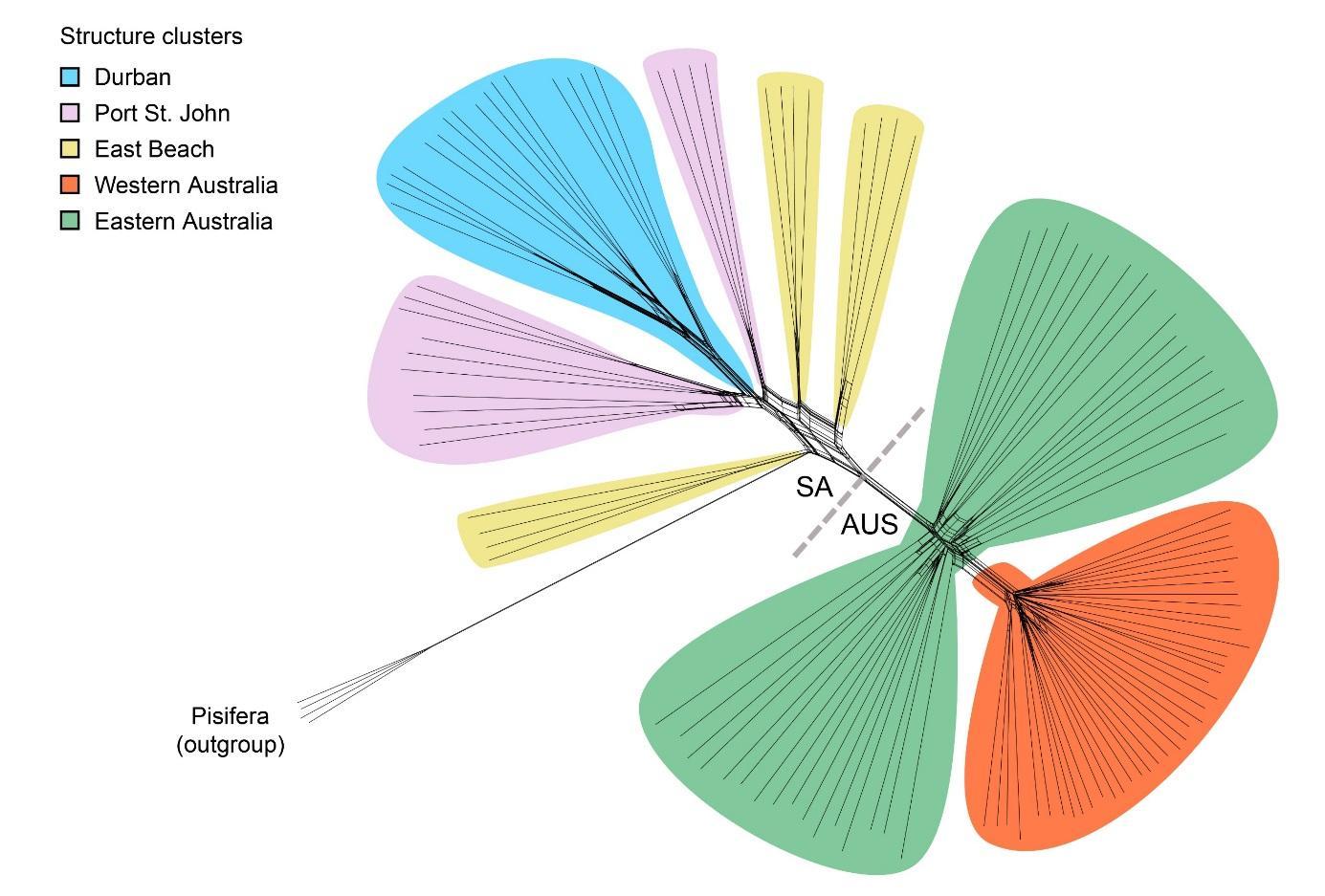
**

**Figure S11:** Neighbour-net network analysis of *Chrysanthemoides monilifera* ssp. *rotundata* (bitou bush) based on genetic distances. Genetic clusters are designated by colour, with broader geographic clusters indicated as South Africa (SA; left of dotted line) or Australia (AUS; right of dotted line) or the outgroup

**
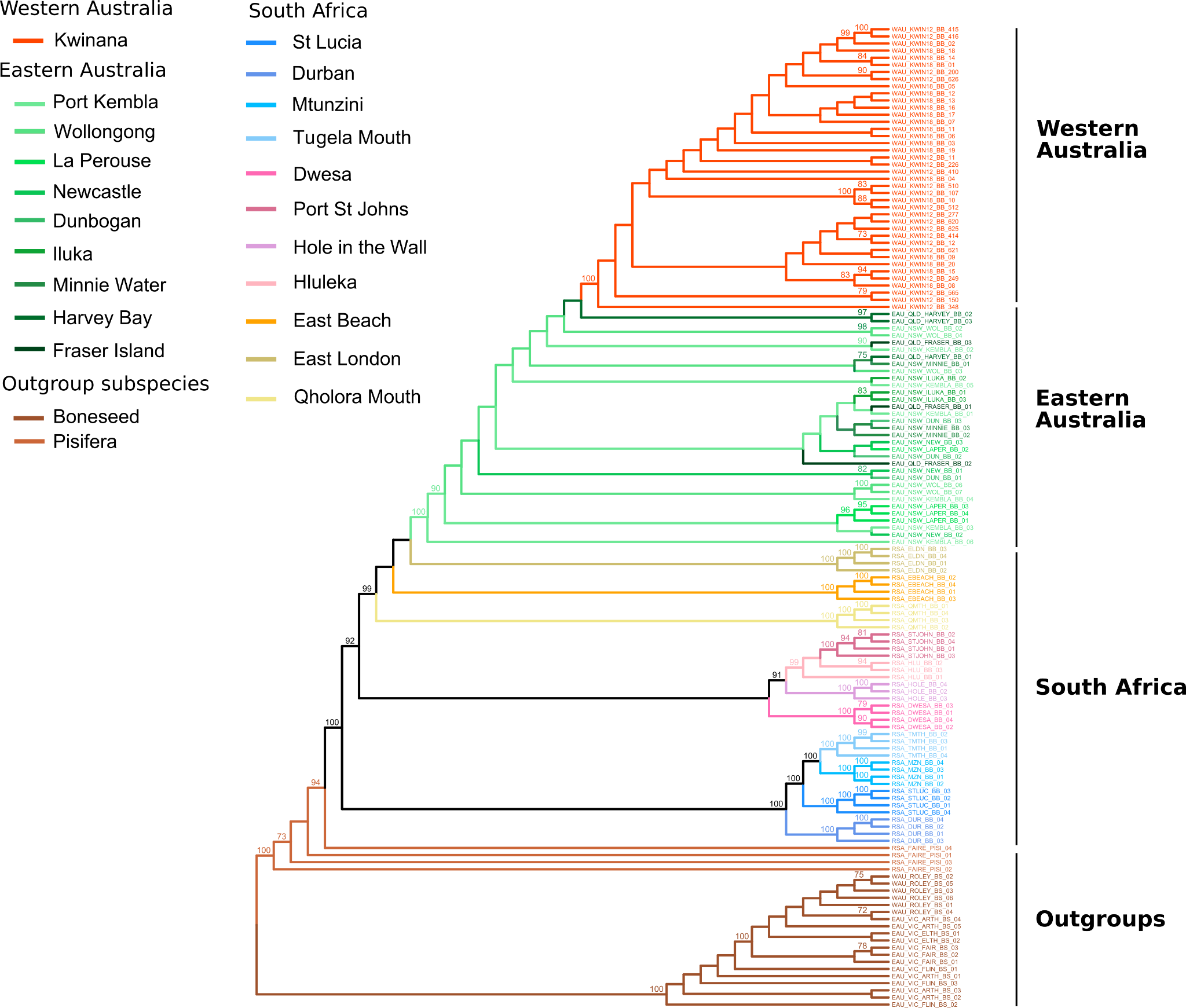
**

**Figure S12** Cladogram of the full dataset of *Chrysanthemoides monilifera* from South Africa and Australia showing the genetic relationship between all sampled individuals of *C. monilifera* ssp. *rotundata* (bitou bush), *C. monilifera* ssp. *monilifera* (boneseed) and *C. monilifera* ssp. *pisifera* (pisifera). Nodal number denote bootstrap values, with values >70 indicating strong support for the cluster.

**
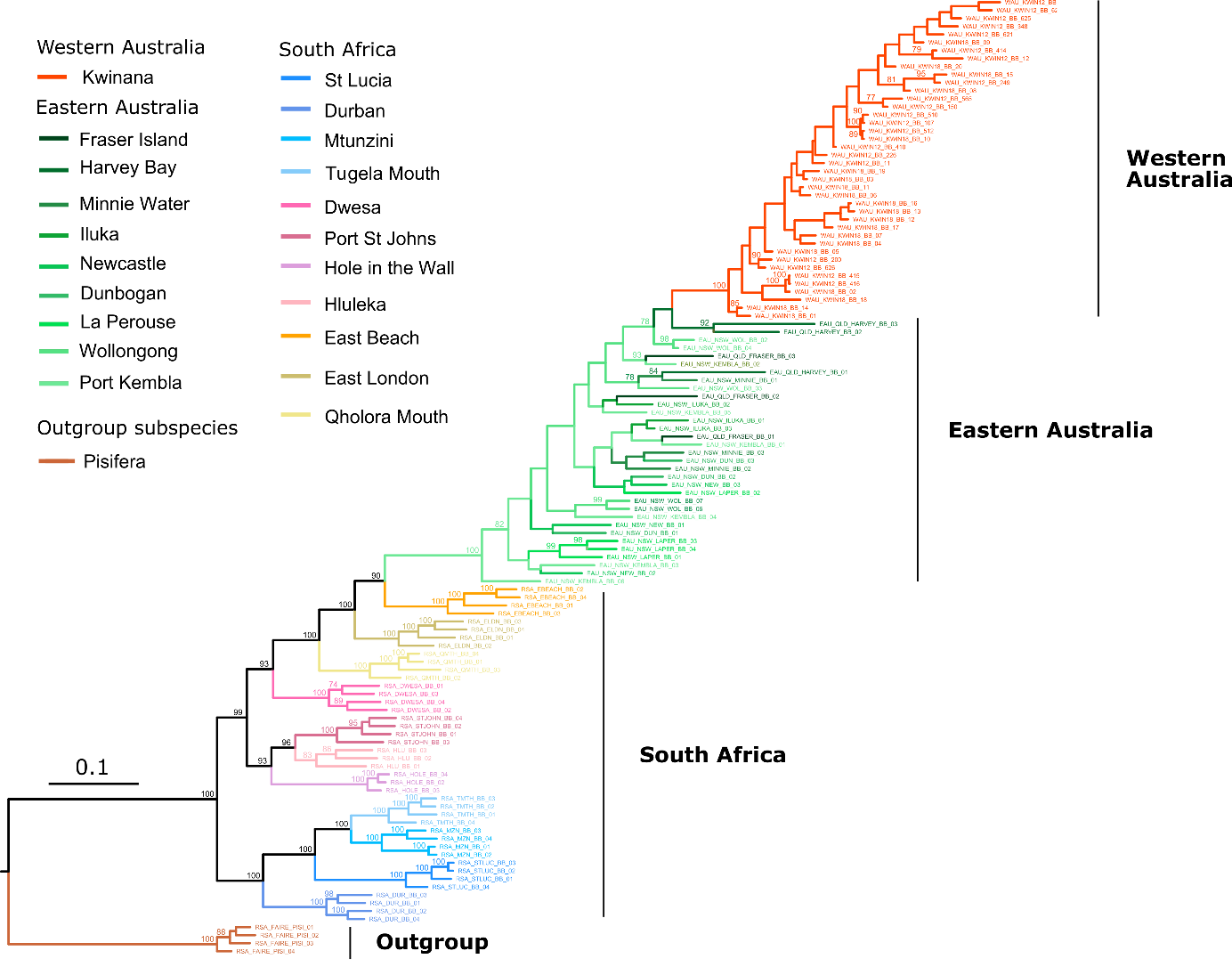
**

**Figure S13** Maximum likelihood phylogeny of *Chrysanthemoides monilifera* ssp. *rotundata* (bitou bush) from South Africa and Australia showing the genetic relationship between all sampled individuals of bitou bush. Branch lengths are scaled to genetic distance. The outgroup is *Chrysanthemoides monilifera* ssp. *pisifera.* Nodal number denote bootstrap values, with values >70 indicating strong support for the cluster.

**
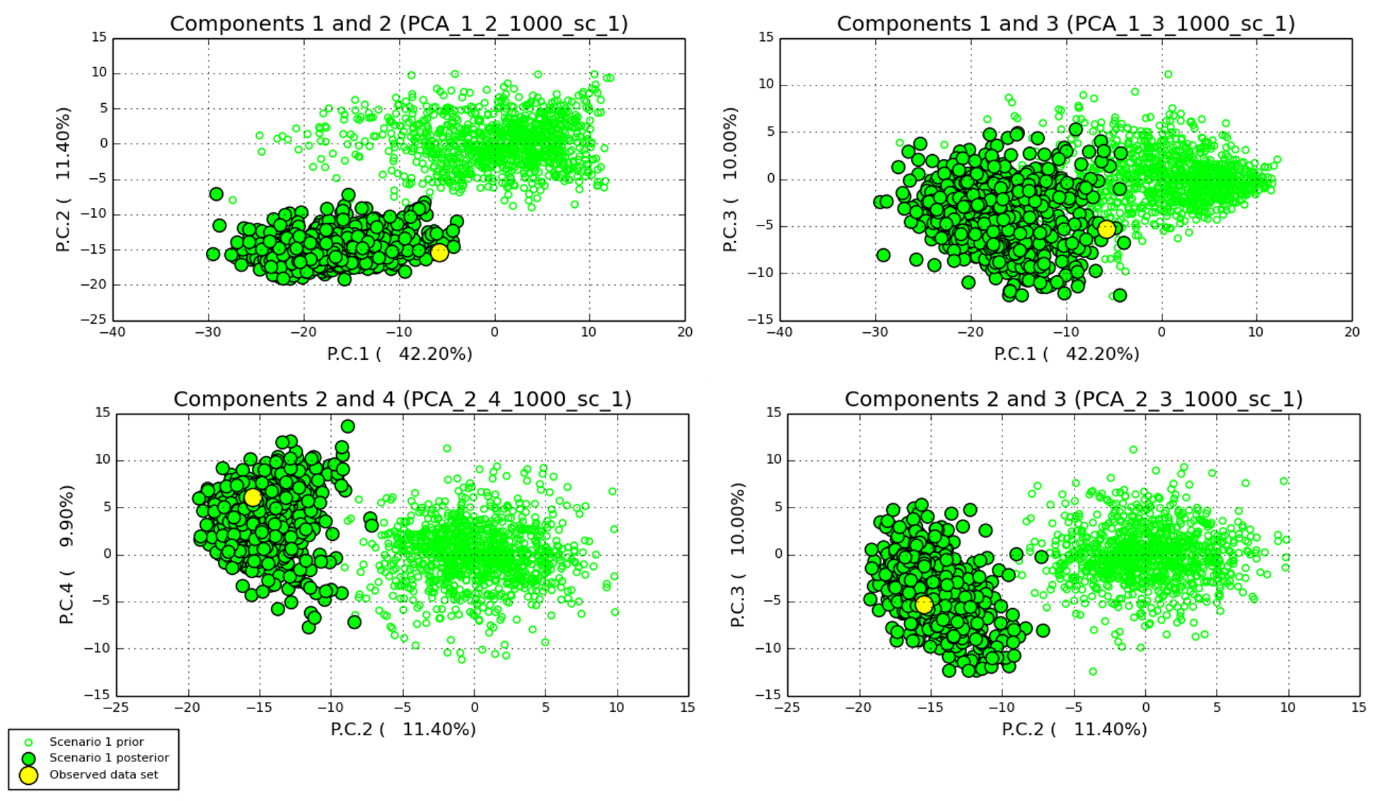
 Figure S14.** Principal component analysis for DIYABC model checking of the final scenario for the introduction history of *Chrysanthemoides monilifera* ssp. *rotundata* (bitou bush) in Western Australia. Hollow green dots represent the prior distribution of models, filled green dots represent the posterior distribution, and yellow dots represent the observed data set.



*
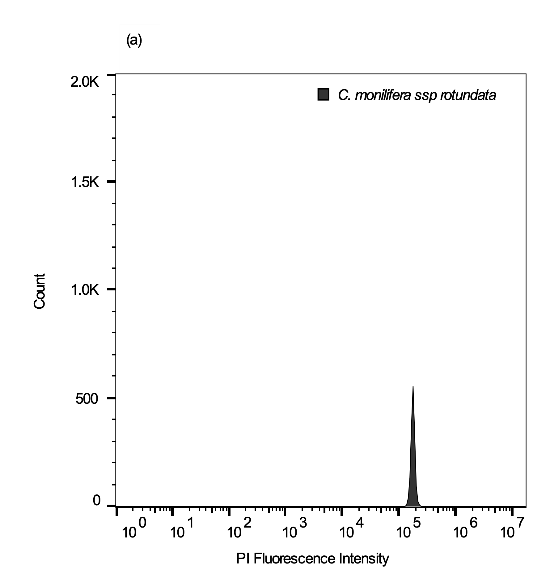
*

*
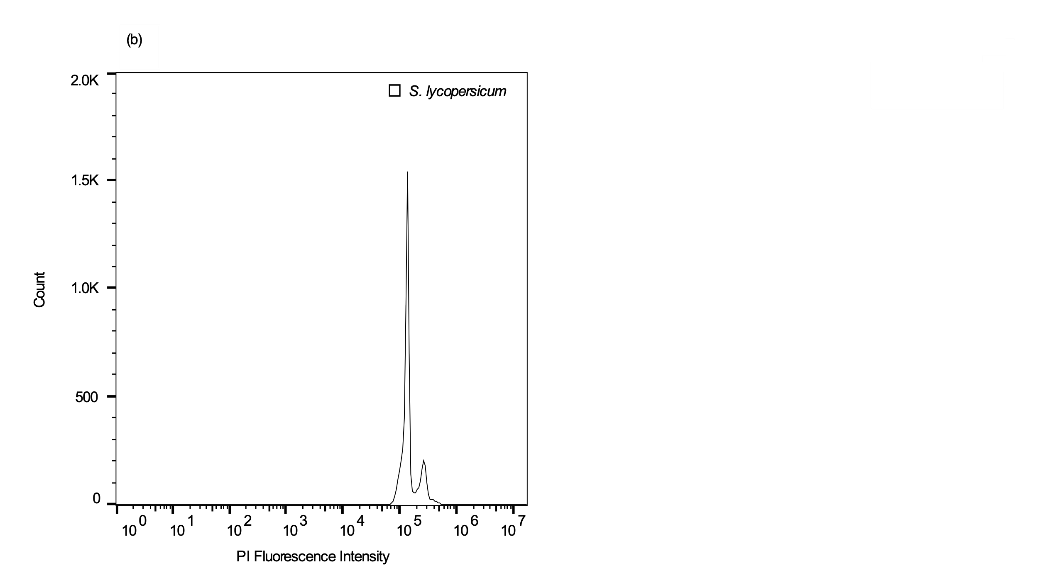
*

*
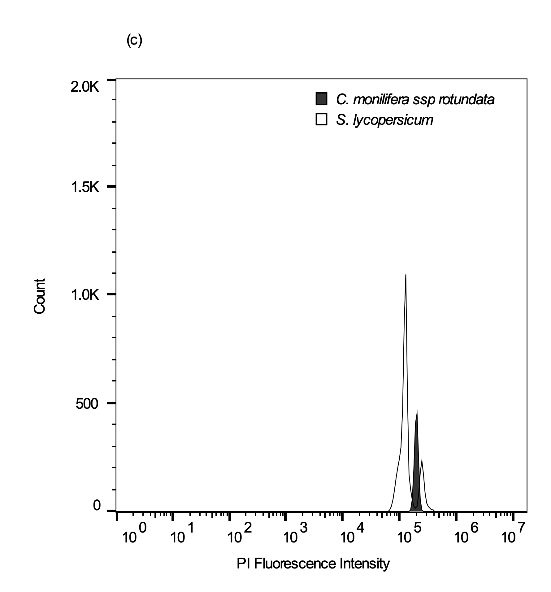
*

**Figure S15.** Smoothed fluorescence histograms showing the estimation of nuclear DNA content (2C) and ploidy for *Chrysanthemoides monilifera* ssp. *rotundata* (bitou bush) using *Solanum lycopersicum* (tomato) cultivar ‘Stupické polní rané’ as a reference standard. **A.** bitou bush. **B.** tomato cv ‘Stupické polní rané’. **C.** bitou bush and tomato cv ‘Stupické polní rané’

**SUPPLEMENTARY METHODS**

**Outlier analysis**

To investigate loci potentially under selection, we used bayescan2.1 (Foll and Gaggiotti, 2008) and bayenv2 (Coop et al., 2010; Gunther and Coop, 2013) to detect markers with high allelic variation between populations. Bayescan identifies loci under selection by using logistic regression to distinguish between population-specific and locus-specific Fst. Bayenv calculates a covariance matrix of allele frequency between populations. For each locus, bayenv reports the X^T^X statistic, which is a measure of population differentiation controlled for demography using this covariance matrix. The VCF file with all 16,827 bitou SNPs was converted to the correct input formats using PGDSpider 2.1.1.5 (Lischer and Excoffier, 2012). For the bayescan analysis, we used default parameters with prior odds for the neutral model of 100 (-pr_odds 100). Three replicate runs were carried out and convergence was assessed using the R package coda (Plummer et al., 2006) by applying the Geweke diagnostic test. Bayescan loci with a q‐value below 0.05 were retained. For the bayenv analysis, five replicate covariance matrices were generated from the full bitou bush dataset of 16,827 SNPs, using 200,000 iterations. X^T^X values above the 0.99 quantile threshold were retained.

**SUPPLEMENTARY RESULTS**

**Outlier analysis**

We detected 110 SNPs (0.07%) deviating from neutral expectations using bayescan and 169 SNPs (1.0%) using bayenv, with 21 SNPs detected by both methods (Supplementary file “outlier_loci.hmp.txt”). Visual inspection of these 21 SNPs using TASSEL 5 (Bradbury et al., 2007) showed that differentiation of all SNPs occurred between Australian and South African populations, with a subpopulation of the EBEACH population sharing some SNPs with the Australian populations.

**Supplementary References**

Bradbury PJ, Zhang Z, Kroon DE, Casstevens TM, Ramdoss Y and Buckler ES (2007) TASSEL: software for association mapping of complex traits in diverse samples. *Bioinformatics* **23**, 2633-2635. doi:10.1093/bioinformatics/btm308

Coop G, Witonsky D, Di Rienzo A and Pritchard, JK (2010) Using Environmental Correlations to Identify Loci Underlying Local Adaptation. *Genetics* **185**, 1411-1423. doi:10.1534/genetics.110.114819

Foll M and Gaggiotti O (2008) A Genome-Scan Method to Identify Selected Loci Appropriate for Both Dominant and Codominant Markers: A Bayesian Perspective. *Genetics* **180**, 977-993. doi:10.1534/genetics.108.092221

Gunther T and Coop G (2013) Robust Identification of Local Adaptation from Allele Frequencies. *Genetics* **195**, 205-+. doi:10.1534/genetics.113.152462

Lischer HEL and Excoffier L. (2012) PGDSpider: an automated data conversion tool for connecting population genetics and genomics programs. *Bioinformatics* **28**, 298-299. doi:10.1093/bioinformatics/btr642

Norlindh T (1963) Chromosome numbers in the Calenduleae 1. With discussions on relationships, hybridization and phytogeography. *Botaniska Notiser* **116**:193-209.

Plummer M, Best N, Cowles K and Vines K. (2006) CODA: convergence diagnosis and output analysis for MCMC. *R news* **6**, 7-11. [https://CRAN.R-project.org/package=coda](https://cran.r-project.org/package=coda)

Rice A, Glick L, Abadi S, Einhorn M, Kopelman NM, Salman-Minkov A, Mayzel J, Chay O, Mayrose I (2015) The Chromosome Counts Database (CCDB) – a community resource of plant chromosome numbers. *New Phytologist* **206**:19-26. doi:10.1111/nph.13191

Riley HP, Hoff VJ (1961) Chromosome studies in some South African Dicotyledons. *Canadian Journal of Genetics and Cytology* **3**:260-271. doi:10.1139/g61-029

Strother JL (1983) More chromosome studies in Compositae. *American Journal of Botany* **70**:1217-1224. doi:10.2307/2443291

Strother JL (2017) Chrysanthemoides. *Flora of North America* [http://www.efloras.org/florataxon.aspx?flora_id=1&taxon_id=106955], Accessed 16/3/2017.

Turner BL (1970) Chromosome numbers in the Compositae. XII. Australian species *American Journal of Botany* **57**:382-389. doi:10.2307/2440865
